# Media choice and audience perceptions: Evidence from visual framing of immigration in news stories

**DOI:** 10.1371/journal.pone.0331219

**Published:** 2025-09-15

**Authors:** Olga Gasparyan, Elena Sirotkina

**Affiliations:** 1 Department of Political Science, Florida State University, Tallahassee, Florida, United States of America; 2 Center for Data Science, New York University, New York, New York, United States of America; Thammasat University Institute of East Asian Studies, THAILAND

## Abstract

Where does visual media bias come from, and how is it reinforced? This study investigates the often overlooked interplay between the visual frames chosen by media outlets for politically charged news stories and how these frames are perceived by their audiences. Using computer vision tools and qualitative content analysis, we analyzed over 2,000 images from 393 media outlets on X. Our findings reveal that U.S. media outlets across the political spectrum consistently emphasize visual narratives that align with their ideological stances while minimizing opposing viewpoints. Their partisan audiences assign identity-driven interpretations to identical visuals, turning them into instruments of antagonistic narratives even without any textual or source cues. This reveals a critical implication: the perceived bias is not merely a product of the media’s framing choices, but also a reflection of how audiences project their ideological filters onto these frames. This study helps us understand how the interplay between media frame curation and partisan interpretations reinforces and perpetuates existing divides.

## Introduction

Media outlets play a crucial role in influencing public perception of politically divisive topics by aligning news stories with the political leanings and demands of their target viewers [1–5] or by actively shaping public perceptions through the emphasis or suppression of specific narratives [6,7]. Despite the significant impact of these strategies, research often treats media framing and public reactions as isolated phenomena, focusing on either media presentation choices or audience responses, rarely examining their interplay [8–12]. However, understanding this interplay is crucial as it demonstrates how media can effectively respond to audience expectations, tailoring content to meet public informational and ideological needs, or subtly shift societal norms on politically sensitive issues, especially within ideologically homogeneous groups or “media bubbles.”

Even less is known about how media outlets choose the visuals that accompany news stories. Visual frames—defined as the main visual representations of a story with a particular focus (see e.g., [13])—are more ambiguous and less monosemantic than textual messages [14]. Yet they are no less powerful in subtly shaping information processing, priming attitudes, and directing attention [13,15–17]. This subtle influence is amplified by the repetitive use of specific image motifs, a technique known as *strategic imagery*, which can normalize particular visual representations to the extent that they appear natural and objective [18]. As a result, over time, these representations may be perceived as the most accurate or even the only accurate portrayal of certain events simply because they are the most frequent visual representations that people see [19].

Our largely exploratory study aims to address these two gaps. First, we analyze a critical yet understudied aspect of news production—the images accompanying news stories in social media—to identify which visual frames media outlets with different ideological perspectives use more or less frequently and whether these patterns remain consistent over time. Second, we examine how audiences perceive these visuals and whether they recognize when certain visual frames are repeatedly emphasized or omitted by their preferred media outlets, even without source attribution or textual cues.

Our empirical analysis is based on tweets shared by 393 U.S. media outlets on their official X (formerly known as Twitter) accounts from 2017 to 2021, focusing specifically on the immigration stories about caravans from Central America. We then extract illustrations that accompany these tweets. Employing computer vision tools and qualitative content analysis, we uncover a distinct mirrored pattern in the portrayal of immigration stories by left-leaning and right-leaning media outlets. Left-leaning media outlets most often depict immigrants using images of ‘women and children,’ a frame that right-leaning media use the least. Conversely, right-leaning outlets predominantly portray immigrants as ‘crowds’ or involved in ‘violations,’ visual themes that are rarely used by left-leaning media. These findings point at a deliberate, non-random selection of visual frames—visual media bias [20,21]—that reveals consistent variations in how the same issue is visually portrayed across time and different media outlets.

When we showed these images to Democrats and Republicans, we found some asymmetry between partisan heuristics and media practice, but a clear alignment in how they interpreted visual media bias: 1) Democrats apply a tiered credibility rule: human-interest images (women and children) outrank everything, violence and state-force images sit at the bottom, and all other frames cluster in the middle. Republicans collapse content into identity, rating images of their own politicians as most accurate, with no significant differences across other frames. 2) Tone perceptions largely mirror these credibility patterns: Democrats read humanitarian scenes as the most positive and violations or LEA authorities as the most negative; Republicans read Republican-politician and military frames as most positive and Democratic-politician plus violations as most negative.

The shared assumption that humanistic representations—such as all close-ups of immigrants—*uniformly* align with liberal ideologies leads viewers from both flanks to believe that liberal media disproportionately favor these symbols. So ‘migrants in camps’ becomes shorthand for “liberal bias” not because outlets consistently deploy it, but because audiences *assume* progressives will prioritize empathy narratives. Conversely, images of police or Republican politicians get reflexively labeled “conservative” and hence are less likely to be associated with liberal media X (Twitter) posts–even when their actual appearance in liberal media outlets is more balanced. This raises important questions about the extent to which media consumers’ biases influence their interpretation of media content, rather than the content itself.

To summarize, the major take-home message from our study is that audience perceptions of visual media bias online reflect ideological alignment more than the structure of actual visual media bias. While amplified visuals may shape audience response over time, interpretations also follow partisan stereotypes of what liberal visual bias *most likely* looks like and shared understandings of what a liberal perspective is. This has implications for how bias is not only existing but also imagined—anchored in collective expectations that sustain polarization even when media content is more varied.

While much better studied textual bias operates through explicit lexical cues that can be captured (e.g., word choice, argument structure; [22]), visual bias works by associating specific imagery with ideological archetypes. Both sides may recognize ideological leaning in the visual frames but interpret them through opposing ideological lenses—liberals may see “resilience” in a crowd of a migrant caravan, while conservatives may perceive it as a “lawless mob.” Thus, the real impact of visual media bias might not lie in changing minds but in solidifying preconceived notions, thereby perpetuating polarization. The question remains whether this selective framing genuinely influences how people understand issues or simply reinforces what they already believe.

## Theory of visual framing

Despite decades of research on framing and framing effects, the communication literature remains divided over precise definitions [23,24]. Rooted in cognitive psychology and sociology, the framing literature, by and large, identifies two primary mechanisms: *equivalence framing*, which presents logically equivalent information in different ways (e.g., “95% survival rate” vs. “5% mortality rate”), and *emphasis framing*, which highlights certain attributes of an issue while downplaying others (e.g., framing poverty as a systemic failure vs. individual responsibility) [23,25,26].

These distinctions reflect framing’s dual role as both a cognitive shortcut–simplifying complex issues by making certain ideas more salient [27]–and a cultural tool that constructs shared meaning and resonates within particular social groups [28]. For instance, Gentzkow and Shapiro [29,30] demonstrated how partisan media outlets systematically use lexical choices to reinforce ideological narratives among intended groups. Iyengar [31] found that television often frames issues using either *episodic framing* (e.g., concrete, personal stories) or *thematic framing* (e.g., general trends and statistics), but episodic framing dominates. Therefore, TV news tends to select stories by zooming in on individual experiences, thereby evoking sympathy. This selection (choosing what to include/exclude) and salience (emphasizing specific elements) of information primes audiences toward predetermined interpretations [32].

The study of framing, though, has historically prioritized textual analysis, partly due to the methodological ease of dissecting written content with automated tools like topic modeling [33] and sentiment analysis [34]. While equivalence and emphasis framing in textual contexts rely more on established elements like words, syntax, and rhetorical devices, visual framing, by contrast, requires researchers to navigate subjective interpretations of images: unlike text, visual framing lacks standardized units of analysis. Researchers must decide whether to code images based on objects (e.g., flags), compositional elements (e.g., camera angles), or symbolic metaphors (e.g., light/dark contrasts), all of which are inherently subjective [35] and play a decisive role in agenda-setting [36]. This ambiguity reflects a broader epistemological tension: while textual framing assumes denotative stability (e.g., the word “crisis” has shared semantic boundaries), visual framing operates through contextual polysemy, where a visual interpretation is fluid and context-dependent [20].

This contextual polysemy largely helps visuals shape news circulation on social media. Protest images, for example, can amplify reach when they provoke emotion, often serving as catalysts for collective action [37,38]. Yet their effects are not uniformly mobilizing. Visuals that portray social problems as fixed or hopeless can suppress political motivation rather than spark it [39]. This variation cannot be reduced to the topic alone [40]. A frame’s impact hinges on multiple individual perceiver-related judgments, including prior experience [41], one’s position about the topic [42], and how the image aligns with her individual sense of what warrants certain reactions [43].

State actors exploit this dynamic. Autocratic governments can adopt informal, personal imagery (e.g., “attractive faces of ordinary people”) to improve trust in the government [44]. Democratic politicians follow similar logic: they occasionally adjust visual self-presentation on social media to reflect values they seek to project (e.g., presenting themselves as caring parents or patriots) [45]. In most cases, visual framing increases online engagement, so even simple actions like liking or retweeting can expand a story’s reach, reinforce its framing, and boost both media coverage and virality [46]. This raises a news story’s visibility and perceived relevance.

Indeed, the distinct viral power of visual framing–relative to its textual counterpart–lies in fundamental differences in cognitive processing and emotional resonance that are always a part of a visual “decoding.” While textual frames operate more through deliberate linguistic choices to prime partisan reactions [47], visual frames bypass analytical processing by leveraging pre-attentive neural pathways. This means that the human brain decodes images much faster than text and retains visual information much longer in memory [48]. This helps visuals to anchor emotional responses way before audiences engage with accompanying text [49]. For instance, when a vivid image of crowded borders is presented alongside an immigration policy news article or tweet, it triggers perceptions of threat or crisis in a manner that operates independently of the specific textual content, as some previous research shows [50].

This understanding of how visual framing influences responses is critical for quantifying the cumulative, long-term effects of media bias, including in algorithmically driven social media ecosystems [51,52]. In politics, large-scale studies of partisan “slant” in newspapers have quantified ideological bias in reporting [53], exposing how systematic media bias shapes public opinion and defines voting outcomes while describing the same policies [54].

Social dynamics intensify the reinforcement of ideological narratives, but through distinct mechanisms: most platforms *prioritize* audience engagement. Platforms optimize for shares and likes, which pushes algorithms to amplify content that aligns with users’ existing views, reinforcing echo chambers [55]. Within these spaces, political content concentrates in emotionally aligned clusters, which amplifies polarization and reduces cross-cutting diffusion [56,57]. Yet, some findings challenge the assumption that online platforms are the primary drivers of polarization. Internet users may be less polarized than those who consume news offline [58], and exposure to diverse content online may even temper polarization at the mass level [59]. The evidence does not point in a single direction. While social media creates conditions for ideological reinforcement, its broader effects remain contingent.

But the reinforcement loop depends as well on how audiences perceive the visuals being presented to them: whether they trust the story being told [60,61], interpret the intent behind the imagery [62], and whether what and how is portrayed resonates with their stereotypes [63,64]. Since images are often interpreted as direct evidence of reality due to their perceived indexicality—the idea that photographs mechanically “capture” truth [65]–audiences frequently judge them as credible without requiring corroboration from accompanying text. This credibility, however, hinges on whether viewers accept the image’s internal narrative logic: whether it “makes sense” as a story. When people find an image trustworthy–that is, they believe it accurately depicts an event–they are more likely to trust and return to the news source, as visuals satisfy a “seeing is believing” heuristic [66].

People must also like and agree with how the events are portrayed to buy in and return [67]. Audiences evaluate perceived visual valence (e.g., positive, neutral, or negative) as well based on whether it aligns with their ideological identity [68]. For instance, sympathetic portrayals of subjects (e.g., images emphasizing suffering or destruction through human-interest framing) shape viewers’ perceptions in a way that might reinforce their support for certain policies [41]. Conversely, portrayals exploiting more negative stereotypes (e.g., crowded border scenes) align with conservative audiences’ concerns about security and cultural preservation, which might add up to validating restrictive policy preferences [69]. Similarly, contradictions emerge when visuals challenge stereotypes. For example, Dasgupta and Asgari [70] found that counter-stereotypical imagery (e.g., Muslim women in STEM roles) reduced implicit prejudice among moderate audiences but had little effect on people’s explicit attitudes.

All of this suggests that to maximize engagement, audiences who expect certain topics to be portrayed in a specific way (e.g., reinforcing preexisting beliefs) should be more likely to remain loyal to media that aligns with their expectations—effectively incentivizing outlets to supply ideologically congruent portrayals. Media likely anticipate or shape audience preferences, prioritizing content that aligns more with existing beliefs to boost engagement. This should create a cycle: viewers consume validating content, social media algorithms supply more of it, and perspectives narrow.

Here we examine exactly this (mis)alignment: which visual frames ideologically distinct media outlets select for politically polarizing issues—using immigration as a running example—and how their audiences interpret these visuals. Specifically, we investigate whether audiences interpret visuals in systematically different ways, whether social media framing aligns with these interpretations, and whether audiences recognize when media favor one visual perspective over another. By analyzing these dynamics, we aim to clarify how and whether visual framing responds to public perception and reinforces ideological divides.

## Materials and methods

### Context and data

To examine media visual choices and audience reactions, we focus on immigration–a highly polarizing issue where Democrats and Republicans typically take opposing positions [71] and limit the scope of our study to events related to the caravans of migrants (moving from Central America to the US-Mexican border) that media outlets covered and featured in their X (formerly known as Twitter) feeds.

First, limiting the scope of the study to a narrower topic allows us to focus on the differences between outlets as a function of their slant rather than on other contextual factors. Further, it was a prominent topic in the discussions of immigration policies in the U.S. and a salient voting issue during the midterm election campaign of 2018, increasing the relevance of the event for the study of political polarization.

We selected X platform (formerly known as Twitter) as our main source of media data since prior to the Twitter acquisition by Elon Musk, the platform dominated political news online consumption [72]. Additionally, we benefited from the way the Twitter feed presents news stories: titles and attached illustrations play a crucial role in the perception of news posts (as discussed by [73]). The previously existing restrictions on word count encouraged news writers to carefully choose their words, particularly in news tweets [74], and to select accompanying images thoughtfully because images are what people almost always look at first due to how human attention is organized that prompts them to “read” the news illustration before anything else [75].

Media outlets strategically prioritize visual content on Twitter, where each post often features only one image, making that singular visual critical for driving engagement (e.g., clicks, likes, retweets). This suggests a deliberate effort to amplify interaction with their posts, as the platform’s mostly single-image format demands visuals that instantly capture attention. While the reuse of identical images in both tweets and online articles may signal cross-platform consistency in strategy, it could also reflect practical constraints, such as resource efficiency (e.g., repurposing a single high-impact image across channels), rather than purely engagement-driven motives.

That being said, Twitter does not replace print or web versions of the media but serves as a lens into the types of content outlets prioritize for maximizing visibility in a crowded, fast-paced environment. Moreover, the competitive pressure to stand out on a platform dominated by mostly single-image posts may push outlets to adopt similar attention-grabbing tactics, fostering uniformity in visual strategies.

For our main analysis, we examined tweets containing the term “migrant caravan” from 393 U.S-based media outlets between December 2017 and October 2021, focusing on a subset of slightly over 5,501 tweets and their attached images (total of 2006 images, where most tweets had only one or no images and in several rare occasions a tweet had 3-4 pictures attached to the post). For additional analysis, we used an alternative approach by querying tweets with the words “migrant,” “caravan,” “migrants,” and “caravans,” resulting in 14,594 tweets and 5,149 accompanying images (see robustness checks in S5 Appendix). All data used in this study were collected from publicly accessible accounts on the X platform (formerly known as Twitter) and analyzed in accordance with X’s terms and conditions. Specifically, both the acquisition of image data and its use in subsequent empirical analysis complied with X’s Terms of Service at the time of access.

Although the keyword “migrant caravan” might initially appear aligned with the Republican agenda, our exploratory analysis shows it is discussed with almost equal frequency by both liberal and conservative media outlets (refer to S1 Fig). This balanced coverage makes “migrant caravan” a well-fitting keyword, as it is specific enough to focus on a particular event and allows us to study the associated visual bias in coverage. Moreover, our dataset of 5,501 tweets, with 2,006 images total attached, provides a solid foundation for analyzing visual framings across ideological spectrums.

We assessed the ideological positions of the news outlets in our sample using the media bias rankings provided by the AllSides [76], which categorizes outlets into five groups ranging from Left to Right. Out of the 393 U.S.-based media outlets in our sample, 62 are classified as “left,” 88 as “lean left,” 159 as “center,” 43 as “lean right,” and 41 as “right.” (See S1 Appendix for a detailed description of the outlets selection process, a complete list of the outlets with their ideological categorization according to AllSides, and distribution of pulled images across outlets.) To demonstrate the robustness of our findings, we conducted additional analyses in S5 Appendix, employing different measures of media outlet ideology.

To identify how the audiences of left- and right-leaning media outlets perceive these visual frames, we surveyed U.S.-based respondents on a crowd-sourcing platform, Lucid Theorem. We sampled 3,000 participants (power calculations are provided in S8 Appendix).

We embedded two automated attention checks (a 3-item list selection and a single-correct-answer multiple-choice question). To avoid excluding participants for a one-off error, we removed only those who both (a) scored fewer than 3 points on the list selection task and (b) answered the multiple-choice check incorrectly. In other words, only failure of both checks—rather than an isolated error on one—led to exclusion, thereby targeting respondents who consistently failed to attend to our embedded tasks. We then manually reviewed responses to the open-ended question (Attention Check I; see S18 Appendix): we excluded any submission that was off-topic (e.g., responses that did not address the question prompt), unintelligible (e.g., including random characters and nonsensical word sequences), or lacking minimal substantive content. Filtering ensures that participants engage meaningfully with visual tasks, as even small lapses in attention can distort results (e.g., misinterpreting images or missing key details). This aligns with best practices in behavioral research, which recommend attention checks for studies relying on subjective evaluations [77,78]. The attention checks required minimal effort—simply reading a question and selecting the instructed items from a list or recalling what was asked immediately beforehand. Failure to identify these straightforward items correctly was taken as evidence of very low engagement. In practice, any respondent exhibiting even minimal attention would pass.

Our final sample comprised 2,089 participants (see the full description of the sample of recruited participants and a comparison of gender, age, and ethnicity sample and population weights in S6 Appendix). The recruitment has been conducted from April 6, 2022, to April 30, 2022. We acquired informed written consent from the participants of the survey, providing them with a clear explanation of the risks, benefits, and purpose of the research study they agreed to participate. The informed consent form was prepared according to the Rice University templates and received approval during the exempt review conducted by Rice University’s Institutional Review Board with the IRB Exempt IRB-FY2022-202. The information obtained was recorded by the investigators in such a manner that the identity of the human subjects cannot be linked to the subjects of the study. Each participant has agreed on the monetary compensation provided through the Lucid Theorem recruitment platform. All the participants who completed the survey received the same compensation. The survey did not exceed 15 minutes, and the compensation was a fixed payment set by the Lucid Theorem Platform to $1. Participants were presented with the following prompt before being asked to evaluate the images: “We will now show you a series of images used in different news stories about immigration. We ask you to answer a few questions regarding your perceptions of each of those photos.” Then we asked each respondent to evaluate 8 randomly drawn images from the pool of 356 images. We pre-selected these 356 images as the most representative of each analytical cluster that we describe below (each cluster contained about the same number of images).

We asked participants to evaluate each image on three dimensions:

- **Accuracy:** *“Do you think this image is a faulty or accurate representation of the story that actually occurred?”* Responses were recorded on a 7-point scale ranging from 1 (faulty) to 7 (accurate).
- **Attitude:** *“Do you think this image portrays people/objects more in a negative light or a positive light?”* The scale ranged from 1 (very negative) to 7 (very positive).
- **Ideology:** *“Do you think this image comes from a liberal or conservative media outlet?”* Participants responded on a scale from 1 (liberal) to 7 (conservative). For the purposes of the empirical analysis, we recode this question into a binary variable with 0 - right-wing outlet guess (scored from 5 to 7 on a 7-point scale in the question ‘Do you think that this image is from a liberal or conservative media outlet? ‘1’ stands for liberal, ‘4’ is moderate, and ‘7’ stands for conservative.’), and 1 - left-wing outlet guess (scored from 1 to 3 on a 7-point scale ‘Do you think that this image is from a liberal or conservative media outlet? ‘1’ stands for liberal, ‘4’ is moderate, and ‘7’ stands for conservative.’) and excluding all moderate outlet guesses (that got 4 in the question on a 7-point scale ‘Do you think that this image is from a liberal or conservative media outlet? ‘1’ stands for liberal, ‘4’ is moderate, and ‘7’ stands for conservative.’

We focus on these variables because they capture distinct facets of how visual content shapes audience perceptions and sustains media engagement, as discussed in the theoretical part. **Accuracy** reflects whether viewers accept an image’s internal narrative as credible, a prerequisite for trusting the broader media source given the indexicality attributed to photographs [65,66]. **Attitude** measures perceived emotional valence, capturing whether images align with stereotypical portrayals of immigration in ways that resonate with partisan groups differently (see e.g., [70]). **Ideology** captures whether audiences associate certain visual frames with partisan media outlets (which speaks about their perception of visual media bias), so that we can further compare their perceptions with actual reporting patterns.

To ensure that any observed effects were not confounded by an uneven distribution of participants’ party affiliations across image clusters, we conducted a manipulation check. Specifically, we performed a $\chi^{2}$ test to assess whether the assignment of image clusters was independent of participants’ party affiliations. The test yielded a p-value of 0.3414 ($\chi_{stat}^{2}=9.0106$, df = 8), leading us to fail to reject the null hypothesis. This indicates no statistically significant association between image cluster assignment and party affiliation. To further confirm the quality of randomization, S11 Table presents results from a multinomial regression model, which also finds no evidence that treatment assignment is related to individual respondent characteristics.

## Analysis and results

### How media visually frame

#### a. Defining visual clusters.

We begin by extracting visual features with ResNet-50 [79] for *feature extraction*, converting each image into an array that captures shape, color, texture and framing cues. Next, we apply *K-means clustering* to these vectors—selecting *K* via elbow and silhouette methods (S3 Appendix)—to partition the corpus into visually coherent groups without any labels. The K-means clustering algorithm initializes *K* centroids, assigns each data point to its nearest centroid, and updates centroids as the mean of their assigned points until convergence. This unsupervised grouping detects dominant visual patterns (e.g. border crossings, close-ups, text overlays) but because this classification is not curated with the assigned labels can also mix semantically distinct images whenever they share visual traits (which we refer to as clustering errors). For example, cluster 3, for instance, contains mostly immigrant-group photos alongside politicians; cluster 1 groups border-crossing scenes with tweet screenshots; cluster 6 conflates media figures and immigrant close-ups. Some images even span multiple clusters: e.g., Donald Trump portrait appears in both clusters 2 and 6 (S4 Fig). Such mismatches (illustrated in S5 Fig) demonstrate why manual review and, where necessary, reclustering are essential for assigning accurate, substantive labels.

**Fig 1:**
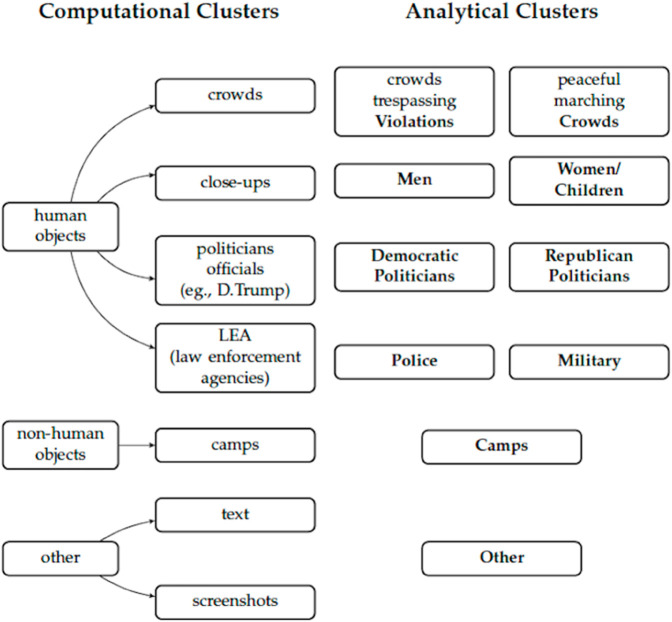
How we formed unsupervised and analytical clusters.

However, while not free of clustering errors, the unsupervised classification approach effectively identified recurrent visual patterns or major tendencies in the representation of immigration. Based on its output, we derived seven distinct image clusters: camps and crowds, close-up portraits, smaller groups of people, either in motion or stationary, as well as wide-angle shots featuring various subjects such as politicians and large gatherings. These results, together with Grabe and Bucy’s approach [20] and prior literature on immigration framing [80], informed our qualitative and theoretical identification of visual frames, as illustrated in Fig 1.

The images we analyzed encompass topics, which we categorize into three broad groups: human objects, non-human objects, and others. Within each group, we further identify specific categories based on careful exploration of the data and theoretical expectations. These final categories include (1) men, (2) women and children, (3) peaceful crowds, (4) violations (e.g., crowds, individuals, or groups trespassing), (5) Democratic politicians, (6) Republican politicians, (7) police, (8) military, (9) camps, and (10) other. When images contain more than one topic, we classify them based on the primary or most prominent frame they convey. By focusing on the primary frame, the classification highlights the most significant aspect of the image, which, according to some previous research [81], is more likely to be the intended message and the one most remembered. However, we acknowledge that determining the “primary” theme can be subjective and may vary among different analysts, potentially introducing bias and inconsistencies in the classification process. To address this issue, we developed a curated codebook (see S16 Appendix). In our sample, at least three coders independently classified each image. Clusters were assigned to images based on 80% agreement or a simple majority rule. Images that did not fit into any of the nine clusters or those without 80% coder agreement were assigned to the “Other” category.

#### b. Temporal shifts in visual frames.

Second, we constructed a timeline for the largest caravan that occurred in October-November 2018 to figure out whether left-leaning and right-leaning media outlets amplified different visual representations at critical points (when the caravan was moving). S6 Fig and S7 Fig in the Appendix depict two timelines. Both timelines represent the most frequent visual frames that were used by liberal (or left-leaning) and conservative (right-leaning) media outlets to report the news stories of migrant caravans.

These timelines show that conservative media outlets used ‘crowds’ more frequently to tell the story about migrant caravans when the caravan started moving in October 2018 and up to its arrival in Mexico City (see S7 Fig). During the same period, liberal media outlets mainly described the same story with images of ‘crowds’ and ‘women and children’ (see S6 Fig). After the caravan arrived in Mexico City, left-leaning media outlets were more likely to illustrate the stories with images of ‘Republican politicians’, and when the caravan reached Tijuana, they extensively used ‘women and children’. After the caravan arrived in Mexico City, and thereafter, right-leaning media outlets extensively used ‘crowds’ and ‘men.’

**Fig 2:**
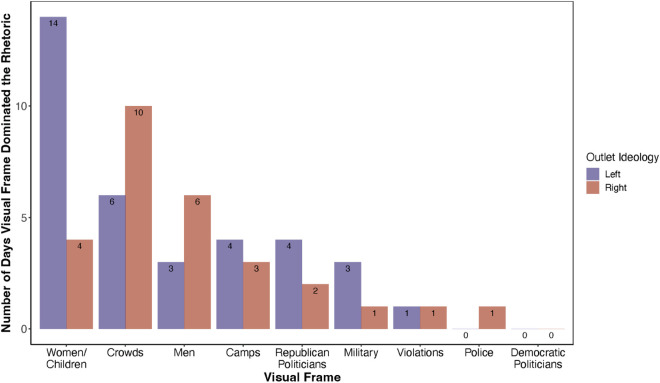
Number of days each visual frame dominated left-and right-leaning media outlets coverage. This plot reflects the number of days when each of the visual frames dominated the rhetoric of left- and right-wing media outlets correspondingly. The plot only includes the days of the publication in the range from Aug to Dec 2018 (media coverage of the second large migrant caravan) and only days that had more than 2 publications in total. The ideology of the media outlets is based on the AllSides, combining “left” and “lean left” outlets in the group of left-leaning, and “right” and “lean right” outlets in the group of right-leaning ones. “Center” media outlets are excluded from the sample.

Fig 2 summarizes the visual representations that appeared most frequently in left- and right-leaning media outlets. It shows the number of days each visual topic dominated media coverage based on the ideological stance of the outlets. We identify visual frame dominance by determining which frame appeared most frequently in images published on a given day, separately for left- and right-wing outlets. For each day, we first count the number of images assigned to each frame across all left-wing and all right-wing sources, respectively. Then, within each ideological group, we calculate the relative frequency of each frame by dividing the number of images in that frame by the total number of images published that day. The frame with the highest proportion is designated as the dominant visual frame for that day (considering only August-December 2018 when the second large migrant caravan occurred and most of our image data and media coverage came from, and days when more than two visual stories were published).

We observe that left-leaning media outlets most often featured close-up images of women and children, while right-leaning outlets emphasized visuals of large crowds. This contrast reflects differences in visual framing choices across ideologically distinct media outlets.

#### c. Visual frames amplified and downplayed.

**Fig 3:**
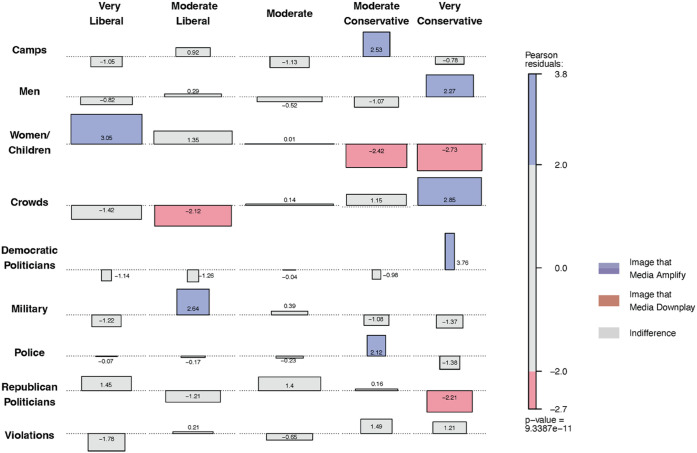
Media outlets with different ideologies amplify and downplay different visual frames. This plot illustrates the relationship between two nominal variables: (1) the ideology of media outlets (from very liberal—“left” according to AllSides—to very conservative—“right” according to AllSides) and (2) visual frame. It is based on images attached to tweets (extracted via the Twitter API), excluding those labeled as “Other.” Colors indicate both the direction and strength of the association between outlet ideology and frame, as measured by Pearson standardized residuals: blue shades denote positive associations (more cases than expected under independence), red shades denote negative associations (fewer cases than expected), and gray indicates no meaningful association. The magnitude of each residual reflects how strongly each cell deviates from the values expected under independence. Numerical values displayed in each cell indicate the Pearson standardized residual for that cell. The p-value displayed corresponds to a Chi-square test of independence, which rejects the null hypothesis of no association between these two variables.

Third, we conducted a cross-tabulation analysis to explore whether there are consistent and statistically significant differences in the visual frames used by media outlets when reporting on migrant caravans. Fig 3 presents an association plot illustrating the distribution of cases between two nominal variables: media ideology and visual frames. In this plot, larger bars indicate a higher frequency of a particular visual frame used by the corresponding type of media outlet. The null hypothesis for the chi-square test is that there is no association between media ideology and the visual frames they use most or least frequently (meaning independence between media ideology and the visual frames they use). If the null hypothesis holds and there is no visual bias, all cells in the plot should be gray. The bars’ color and direction indicate deviations from no association, with blue representing “using this frame more frequently” and red representing “using this frame less frequently.”

The association plot in Fig 3 shows a statistically significant association between outlet ideology and the visual frames used in immigration coverage ($\chi^{2}=111.67$, df=32, p-value <0.001), indicating that image selection aligns systematically with ideological stance rather than occurring at random.

The cross-tabulation analysis in Fig 3 illustrates that right-leaning media outlets tend to use visual frames featuring ‘crowds,’ ‘Democratic politicians,’ ‘camps,’ ‘men,’ and ‘police’ more frequently (indicated by blue shading). In contrast, they use images of ‘women and children’ and Republican politicians’ significantly less often (indicated by red shading).

Conversely, left-leaning media outlets frequently use visual frames of ‘women and children’ and ‘military’ (blue shading), while significantly underusing representations of ‘crowds’ (red shading). Outlets in the middle of the ideological spectrum, depicted in the center of the square in Fig 3, do not follow a specific pattern when selecting visual frames (all cells are gray).

To ensure the robustness of our results, we used an alternative source to define media ideology (or media bias) and a different set of keywords to retrieve images depicting migrant caravans. The robustness checks, detailed in S8 Fig-S10 Fig in the Appendix, support our main findings. The results indicate that left-leaning media consistently amplify images featuring ‘women and children’ while downplaying those showing ‘violations’ and ‘crowds’ or ‘men’ (S8 Fig). Conversely, right-leaning media consistently amplify images of ‘crowds’, ‘police’ or ‘violations’ (S8 Fig) but tend to systematically ignore ‘women and children.’ These findings indicate that visual framing is systematically shaped by ideology rather than applied at random. Liberal and conservative outlets tend to use opposing frames in a patterned, reciprocal manner: when one side amplifies a given frame, the other minimizes it, reflecting their divergent political commitments.

### Framing effects: How people read visual frames

**Fig 4:**
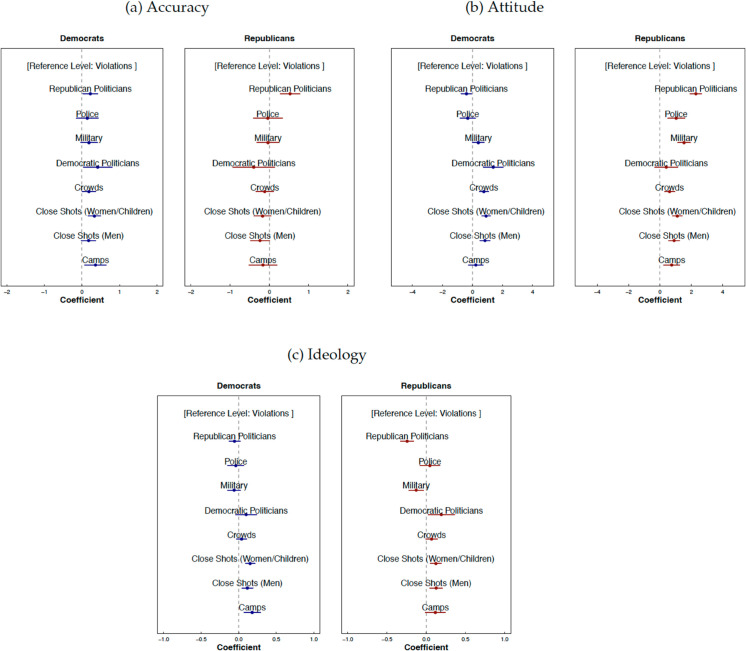
How people react to visual frames within partisan groups. Each whisker represents the estimated regression coefficient for a visual-frame predictor (factor) from linear models fit separately on Democratic and Republican subsamples, using the ‘violations’ frame as the reference category. Partisanship was self-reported (1 = Democrat; 0 = Republican). Accuracy and attitude were measured on 7-point scales ranging from 1 (image gives a faulty representation/evokes a negative attitude) to 7 (image gives an accurate representation/evokes a positive attitude). Media outlet ideology guess was coded as a binary variable: responses of 1–3 on the question “Do you think this image is from a liberal or conservative outlet?” were classified as left-wing (1), and responses of 5–7 as right-wing (0); moderate responses (4) were excluded. All models report 95% confidence intervals, include random effects (random intercepts for images and respondents), and control for gender, age, ethnicity, education, income, and interest in politics.

We present the survey results in Fig 4. (The full regression tables and control variables are provided in S16 Table and S19 Table.) The figures display beta coefficients for a nine-value factor variable, where each factor represents an image cluster for Democrats and Republicans separately. We provide descriptive statistics for each of the outcome variables by visual frame and partisanship in S9 Appendix. S10 Appendix reports the results of an ANOVA test examining how participants with different party affiliations vary in their evaluations of images from distinct media outlets and visual frames.

The effects are estimated on two partisan subsamples using the following model specification:

$$Y_{ig}=\beta_{0}+\beta_{1}X_{g}+\mu C_{i}+\tau_{g}+\upsilon_{i}+\epsilon_{ig}$$ (1)

where *Y*_*ig*_ is an outcome of interest (accuracy, attitude, or ideology) that a respondent *i* expressed towards an image *g*, *X*_*g*_ is a predictor of interest (a visual frame to which image *g* belongs), *C*_*i*_ is a vector of control variables for a respondent *i*, $\tau_{g}$ is image-level random effects, and $\upsilon_{i}$ is respondent-level random effects. Control variables include self-reported gender, age, education, ethnicity, income, and interest in politics (see the questionnaire in S18 Appendix).

The category ‘violations’ is used as the reference in the specifications reported in the main text. This choice reflects its comparable day-level dominance, with each group featuring this frame as dominant on only one day (unlike ‘woman and children,’ ‘crowds,’ and ‘men’) (Fig 2). For additional control, alternative reference groups are also considered: specifications with ‘women and children’ (the most commonly used frame by liberal outlets) and ‘crowds’ (the most commonly used frame by conservative outlets) can be found in S17 Table-S18 Table and S20 Table-S21 Table in the Appendix.

We report several major findings:

First, the results illustrated in Fig 4a show that Democrats trust every frame more than ‘violations’—except the hard-security ones (‘military’ (b = 0.186, se = 0.116, p = 0.109) and ‘police’ (b = 0.143, se = 0.149, p = 0.340)), which they see just as lowly credible as ‘violations.’ In plain terms, they give higher credibility to images evoking humanitarian appeals, politicians of any political flank, or group identities (crowds and close-ups), but not to images emphasizing state force. Across all specifications (illustrated here in Fig 4a and detailed in S16 Table–S18 Table of the Appendix), Democrats draw only two clear credibility lines: images of individual vulnerability (women and children) are rated highest, images of violence or state force (violations, police, military) are rated lowest, and all other frames—close-ups of men, crowds, camps, Democratic or Republican politicians—falls into a middle category with no consistent distinctions. In other words, Democrats apply a simple three-tier heuristic: they elevate human-interest imagery, denigrate violence-oriented scenes, and treat all remaining visual frames as comparatively credible.

Republicans, on the contrary, consistently see all frames as equally credible compared to ‘violations,’ (see Fig 4) except when the frame features their own party: they rate only ‘Republican politicians’ as more accurate than ‘violations’ (b = 0.527, se = 0.128, p < 0.001), showing that their only credibility boost comes when the content involves their in-group.

These patterns imply that perceived accuracy functions as a heuristic cue and the cues differ by party: Democrats use more content-based heuristics (boosting credibility for humanitarian and denigrating the LEA agents), whereas Republicans use an identity-based heuristic (granting extra accuracy only when their own party appears). In S12 Fig-S13 Fig for accuracy we separate the sample on high- (from 5 to 7 on a seven-point scale of confidence) and low- (from 1 to 3 on a seven-point scale of confidence) confidence responses. Our primary concern would be if low-confidence respondents largely drove the results. However, S22 Table shows that the majority of responses come from participants with a high level of confidence. In separating low- and high-confidence responses in the subsamples in S13 Appendix, we also show that the results are robust to the respondents’ level of confidence in answering questions about accuracy.

Second, when we ask Democrats whether these visual frames tend to portray immigration more negatively or positively—the results are shown in Fig 4b—we observe a tonal pattern that to some extent mirrors their accuracy assessments. For instance, the images Democrats rate as least accurate—violations, military, and police—are also among those they perceive as the most negative portrayals of immigration (see S17 Table-S18 Table), with Republican politicians appearing as the most negative overall (b = –0.404, se = 0.176, p = 0.022). Yet, Republican politicians are also judged as equally accurate as humanizing frames featuring women and children ((b = –0.107, se = 0.074, p = 0.149) in S17 Table), as well as frames signaling danger, violations, and crowds ((b = 0.040, se = 0.079, p=0.615) in S18 Table). This indicates that Democrats do not dismiss negatively toned visuals as inaccurate; rather, they can separate the perceived tone of portrayal from perceived representational adequacy. But by and large, the more a frame humanizes an immigration subject overall, the more positive its tone appears and the more accurate it seems; the more it emphasizes control or threat, the harsher its tone and the less credible it feels.

Republicans view every image—from close-ups of women and children to scenes of crowds, camps, police, the military, and even their own party’s politicians—as conveying a more positive attitude toward immigration than ‘violations’ (see Fig 4b). Only images of ‘Democratic politicians’ are perceived as negatively as ‘violations’ (b = 0.401, se = 0.380, p = 0.292). Shifting the baseline to ‘women and children’ flips the contrast: ‘crowds,’ ‘Democratic politicians,’ and ‘violations’ now appear more negative than that humanizing frame (see S17 Table-S18 Table), whereas ‘military’ (b = 0.428, se = 0.165, p = 0.010) and ‘Republican politicians’ (b = 1.181, se = 0.135, p < 0.001) emerge more positive. When compare ‘women and children’ with ‘close-ups of men,’ ‘camps,’ or ‘police’ no distinction remains (see S17 Table for details). This suggests that Republicans are more likely to resonate with themes of strength, order, and authority, as represented by ‘military’ and ‘Republican politicians,’ while viewing violent crowds and ‘Democratic politicians’—symbols of perceived disorder and their out-group—more negatively.

Taken together, the visual frames amplified and downplayed by left- and right-leaning media, along with partisans’ reactions to them, reveal the following: left-leaning outlets populate Democratic timelines with the ‘women and children’ frame, while showing fewer ‘crowds’ or ‘violations’ images. This aligns with Democrats’ perceptions, as they rate ‘women and children’ highest in terms of both accuracy and positive tone, place ‘violations’ lowest, and rank ‘crowds’ in the middle. In contrast, right-leaning media amplify frames that Republicans perceive as equally accurate to ‘violations,’ while downplaying ‘Republican politicians,’ the most trusted and positively evaluated visual frame for telling the story of immigration. They also show fewer images of ‘women and children’—one of the most positively evaluated frames—and more ‘violations’ (see S8 Fig-S9 Fig)—one of the most negatively evaluated frames by Republicans. This suggests that right-leaning media are not reinforcing Republicans’ most trusted or positive views. Instead, they are more likely reinforcing negative stereotypes about immigration, as they amplify and likely stabilize a harsher, more punitive portrayal of immigration.

Lastly, we find that both Democrats and Republicans appear to rely on similar heuristics when predicting which visual frames are likely to appear in liberal media outlets. Overall, they tend to assume that humanizing visuals are more common in liberal media, while frames depicting violations are less likely to appear there (see Fig 4c). While this assumption aligns with some actually observed media patterns, both groups misidentify *the extent* to which humanizing frames are prevalent in liberal media. For example, both Democrats and Republicans are more likely to associate close-up shots of men (b = 0.115, se = 0.038, p = 0.003 for Democrats; b = 0.127, se = 0.042, p = 0.003 for Republicans) and women with children (b = 0.152, se = 0.032, p < 0.001 for Democrats; b = 0.123, se = 0.037, p = 0.001 for Republicans) and images of camps (b = 0.180, se = 0.056, p = 0.002 for Democrats; b = 0.117, se = 0.064, p = 0.069 for Republicans) with left-leaning outlets (compared to ‘violations’), even though only images of women and children are actually more frequent in liberal media tweets (see S8 Fig-S9 Fig). This suggests that both groups’ understandings of visual media bias may be influenced more by preconceived ideas of what liberal media outlets *are anticipated* to look like, rather than by their actual framing choices. As a result, they may be more likely to focus on and remember images that match these expectations, reinforcing their belief in media bias. And this selective attention to confirming content may further strengthen their political biases.

In S14 Fig-S15 Fig, we separate the sample into high-confidence responses (ranging from 5 to 7 on a seven-point scale) and low-confidence responses (ranging from 1 to 3 on the same scale). As with the accuracy outcome, our concern here is whether low-confidence respondents disproportionately influenced the results. By separating low and high-confidence responses in the subsamples in S13 Appendix, we demonstrate that the results are robust to the respondents’ confidence levels in guessing outlet ideology. In S15 Appendix we also show correlation matrices for the image-level aggregated probabilities of outlet ideology guesses and the probabilities of these images appearing in left- or right-wing media and report weak correlation levels (not exceeding ρ=0.3).

## Discussion

Political visual frames often convey arguments independently of text [82], a dynamic especially intensified on fast-scrolling social media [83], where users, even if they skip captions, are inevitably exposed to accompanying visuals [24]. As with textual frames, repeated exposure to congruent visual frames reinforces beliefs and normalizes certain representations [18]. By contrast, images that clash with a viewer’s leanings may be ignored, spark skepticism or provoke a defensive reaction, and neutral or random images often leave no lasting impression [84]. To expose these dynamics, we analyzed here which visuals left- and right-leaning outlets share on social media platforms when they report news stories about a politically polarizing topic and measure how Democratic and Republican audiences react to them.

We find that left-leaning media outlets emphasize visual frames that align more closely with Democrats’ understanding of what constitutes credible and positive framing of immigration [85,86] by prioritizing personalized humanitarian imagery (e.g., women and children) and de-emphasizing less personal visual cues, such as crowds, which fit less with the more compassionate liberal narratives. As a result, this reinforcement of certain visual frames may solidify partisan interpretations, such as the tendency among Democrats to partially equate a “positive tone” with “accuracy,” a perspective not shared by Republicans.

Republicans’ skepticism toward immigration is, to a large extent, grounded in perceptions of cultural threat (e.g., demographic change) and societal disorder (e.g., unauthorized border crossings) [69,87]. These predispositions act as cognitive filters, sensitizing conservatives to interpret certain visuals (such as ‘crowds’ or ‘violations’) through a threat-detection frame. But the fact that Republican audiences rate visuals of authority figures (e.g., ‘police’ and ‘military’) and Republican politicians more favorably in terms of image tone than most of those emphasizing vulnerability (e.g., ‘women and children’) or disorder (e.g., ‘crowds’) reflects a “reversed” symmetry in partisan visual interpretation. Authority-linked images, which likely resonate with Republicans’ preference for order and control, are often evaluated as more positive visual portrayals in immigration-related stories [88], yet right-leaning media overrepresent threat-focused frames—particularly those portraying out-groups (e.g., ‘Democratic politicians’, ‘crowds’, ‘men’, ‘camps’)–suggesting a strategic use of threat to sustain attention and mobilize sentiment.

This gap—between positive reception of authority-linked images and the emphasis on threat-based framing—reflects a strategic use of negativity bias in social media. Threatening or conflict-laden content attracts more attention and spreads faster [89,90]. On social media, this effect is amplified by algorithmic preferences for emotionally charged material [89]. Right-leaning media online seem to prioritize outrage over reinforcing positive associations, since outrage is more effective at generating engagement and loyalty—precisely the media’s currency on digital platforms [91].

Moreover, we reveal that both partisan groups operate under shared, stereotypical assumptions about what constitutes “liberal media bias,” even as their perceptions diverge from actual editorial practices. This means their shared assumptions about visual media bias are tied less to actual media content and more to cultural imagery [92]: visuals of migrants in camps, vulnerability, or border chaos have become symbolically coded as “liberal,” even when not strategically pushed by liberal media. So when people see these images, they may not even trace them back to editorial intent—they react to what those images have come to *mean* politically.

This study confronts a critical tension in political communication scholarship: the disproportionate focus on textual and algorithmic biases in digital discourse, despite mounting evidence that visual framing operates as a distinct, under-theorized mechanism of ideological polarization [73,93,94]. We argue that partisan attitudes are increasingly shaped by subtle, cumulative exposure to biased visual frames, which is especially acute on image-driven social media platforms such as Instagram, TikTok, and X (formerly Twitter) [95], where users continuously encounter visual content that reinforces existing stereotypes and partisan divisions [96].

Our findings, based on empirical data from social media, align with prior research on traditional media, which found that certain media outlets tend to portray marginalized groups in an often distorted and unflattering light, exacerbating their stigmatization [97]. First, prior research has focused on representational biases but often overlooked how audiences interpret, resist, or internalize these frames—key to understanding attitude solidification. By analyzing empirical data from social media, our study addresses this gap, showing the importance of examining both visual media bias and audience responses.

Second, prior work demonstrates that visual content primes identity-based biases, as audiences can infer partisan affiliations, ideological positions, or sociodemographic traits (e.g., gender, ethnicity) from imagery alone, absent text or source cues [98]. Cognitive research increasingly indicates that individuals process images independently of text [50]—a critical consideration for social media contexts—though isolating visuals’ “pure effects” in real platforms remains methodologically and practically fraught, as evaluations inevitably intertwine with textual cues and user experience [99], which is a limitation of our approach.

Overall, our study argues that many political outcomes hinge on the interaction between media tactics and how audiences perceive them. This interplay is what shapes narratives, including visual narratives, not just the strategies or perceptions alone. Isolating one from the other in research risks misrepresenting their combined influence—particularly when partisan visual frames gain traction not merely through dissemination but through how they are interpreted as well.

## Supporting information

S1 Appendix Media Outlets Descriptions. (ZIP)

S1 Table List of media outlets. (ZIP)

S2 Table Distribution of all pulled images across media outlets and years. (ZIP)

S3 Table Distribution of images used in the survey wave across media outlets and years. (ZIP)

S2 Appendix “Migrant Caravans” is Equally Present in Liberal and Conservative Media Outlets. (ZIP)

S1 Fig Distribution of tweets mentioning “migrant caravans” across media outlets with different ideological standpoints. (ZIP)

S2 Fig Number of images used in tweets across media outlets with different ideological standpoints. (ZIP)

S3 Appendix Unsupervised Clustering. (ZIP)

S4 Table Distribution of images across clusters. (ZIP)

S3 Fig Optimal number of K clusters testing. (ZIP)

S4 Fig Examples of images from K-means clusters. (ZIP)

S5 Fig Examples of unsupervised clustering errors. (ZIP)

S4 Appendix A Timeline of How Media Outlets Use Visual Frames. (ZIP)

S6 Fig Visual frames that dominated left-leaning media reports about migrant caravans from October to November 2018. (ZIP)

S7 Fig Visual frames that dominated right-leaning media reports about migrant caravans from October to November 2018. (ZIP)

S5 Appendix Robustness Checks. (ZIP)

S8 Fig Visual frames and ideology of media outlets: Alternative ideology measure. (ZIP)

S5 Table Returned tweets for each of the key words searches. (ZIP)

S9 Fig Visual frames and ideology of media outlets: Alternative image search. (ZIP)

S10 Fig Visual frames and ideology of media outlets (only in 2018). (ZIP)

S6 Appendix Participants Sample Description Statistics. (ZIP)

S6 Table Descriptive statistics for survey respondents. (ZIP)

S7 Table Descriptive statistics for survey respondents (excluding participants with self-reported partisanship as Independent). (ZIP)

S8 Table Distribution by gender. (ZIP)

S9 Table Distribution by age. (ZIP)

S10 Table Distribution by Hispanic ethnicity. (ZIP)

S7 Appendix Manipulation Check. (ZIP)

S11 Table Results of multinomial logistic regression for the randomization check. (ZIP)

S8 Appendix Power Analysis Simulations. (ZIP)

S11 Fig Power simulation. (ZIP)

S9 Appendix Descriptive Statistics for the Three Main Outcome Variables. (ZIP)

S12 Table Descriptive statistics by visual frame and partisan groups.

S10 Appendix ANOVA Results. (ZIP)

S13 Table ANOVA results: Accuracy. (ZIP)

S14 Table ANOVA results: Attitude. (ZIP)

S15 Table ANOVA results: Ideology. (ZIP)

S11 Appendix Within-Party Analysis of All Visual Frames: Regression Results for Accuracy and Attitudes. (ZIP)

S16 Table Linear regressions for partisan subsamples on all clusters with “Violations” as a baseline category. (ZIP)

S17 Table Linear regressions for partisan subsamples on all clusters with “Close Shots (Women/Children)” as a baseline category. (ZIP)

S18 Table Linear regressions for partisan subsamples on all clusters with “Crowds” as a baseline category. (ZIP)

S12 Appendix Media Outlet Ideology Guess Results. (ZIP)

S19 Table Linear regressions for partisan subsamples on all clusters with “Violations” as a baseline category. (ZIP)

S20 Table Linear regressions for partisan subsamples on all clusters with “Women/Children” as a baseline category. (ZIP)

S21 Table Linear regressions for partisan subsamples on all clusters with “Crowds” as a baseline category. (ZIP)

S13 Appendix Comparison of High- and Low-Confidence Responses for Accuracy and Outlet Ideology Guesses. (ZIP)

S22 Table Distribution of response confidence for accuracy and outlet ideology guesses. (ZIP)

S12 Fig Accuracy results for high-confidence respondents. (ZIP)

S13 Fig Accuracy results for low-confidence respondents. (ZIP)

S14 Fig Media outlet ideology guesses for high-confidence respondents. (ZIP)

S15 Fig Media outlet ideology guesses for low-confidence respondents. (ZIP)

S14 Appendix Outlets Ideology and the Choice of Visual Frames. (ZIP)

S23 Table Visual frames that liberal media outlets use more often than conservative media outlets: Binary OLS Results. (ZIP)

S15 Appendix Correlations between Outlet Ideology and Outlet Ideology Guesses. (ZIP)

S24 Table Scoring correlation table: Democrats. (ZIP)

S25 Table Scoring correlation table: Republicans. (ZIP)

S16 Appendix Curated Labeling. (ZIP)

S26 Table Curated labels cluster sizes. (ZIP)

S27 Table Curated labels distribution in the survey wave. (ZIP)

S16 Fig Examples of images with curated labels. (ZIP)

S17 Appendix Questionnaire. (ZIP)

S18 Appendix Curated Codebook and Labeling. (ZIP)

S28 Table Explanation of the label assignment with image examples. (ZIP)
