# Supplementary material for: Media choice and audience perceptions: Evidence from visual framing of immigration in news stories
Source: PLoS One. 2025 Sep 15;20(9):e0331219. doi: 10.1371/journal.pone.0331219 (PMC12435698; doi:10.1371/journal.pone.0331219)
Supplement: S1 Appendix — (ZIP) [file pone.0331219.s001.zip › si_files/S14_Table.pdf]

Table S.14: ANOVA results: Attitude.

	Df	Sum Sq	Mean Sq	F value	Pr(>F)
Outlet Ideology	2.00	99.41	49.70	13.51	0.00
Image Frame	8.00	646.22	80.78	21.96	0.00
Partisanship	1.00	174.74	174.74	47.50	0.00
Outlet Ideology*Image Frame	15.00	236.60	15.77	4.29	0.00
Outlet Ideology*Partisanship	2.00	2.79	1.39	0.38	0.68
Image Frame*Partisanship	8.00	1783.38	222.92	60.60	0.00
Outlet Ideology*Image Frame*Partisanship	15.00	68.99	4.60	1.25	0.23

Table S.15: ANOVA results: Ideology.

	Df	Sum Sq	Mean Sq	F value	Pr(>F)
Outlet Ideology	2.00	3.19	1.59	6.91	0.00
Image Frame	8.00	49.93	6.24	27.08	0.00
Partisanship	1.00	14.20	14.20	61.61	0.00
Outlet Ideology*Image Frame	15.00	3.98	0.27	1.15	0.30
Outlet Ideology*Partisanship	2.00	0.56	0.28	1.22	0.30
Image Frame*Partisanship	8.00	7.96	1.00	4.32	0.00
Outlet Ideology*Image Frame*Partisanship	15.00	7.05	0.47	2.04	0.01

S11 Within-Party Analysis of All Visual Frames: Regression Results for Accuracy and Attitudes
