# Supplementary material for: Media choice and audience perceptions: Evidence from visual framing of immigration in news stories
Source: PLoS One. 2025 Sep 15;20(9):e0331219. doi: 10.1371/journal.pone.0331219 (PMC12435698; doi:10.1371/journal.pone.0331219)
Supplement: S1 Appendix — (ZIP) [file pone.0331219.s001.zip › si_files/S16_Fig.pdf]

Fig. S.16: Examples of images with curated labels.

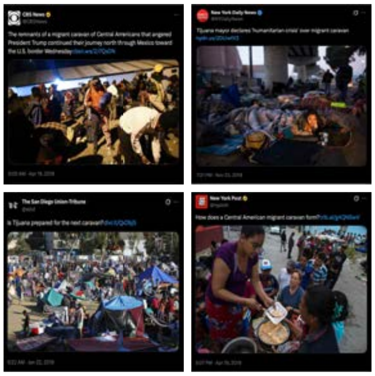

(a) Camps

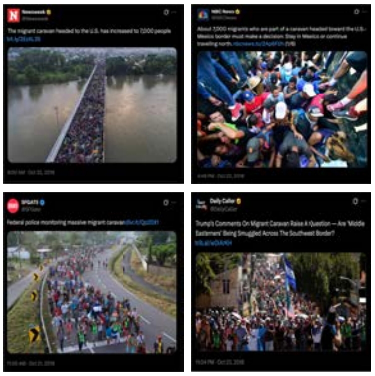

(b) Crowds

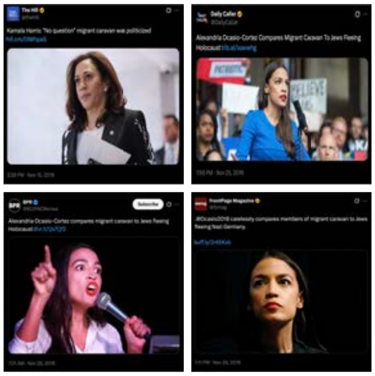

(c) Democratic Politicians

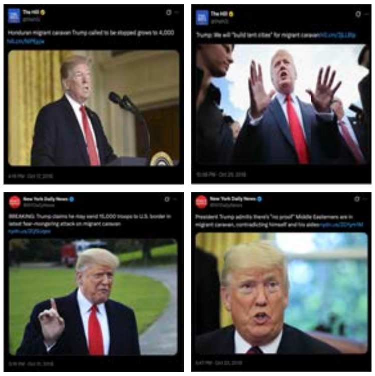

(d) Republican Politicians

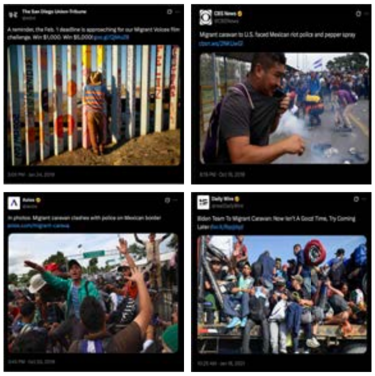

(e) Men

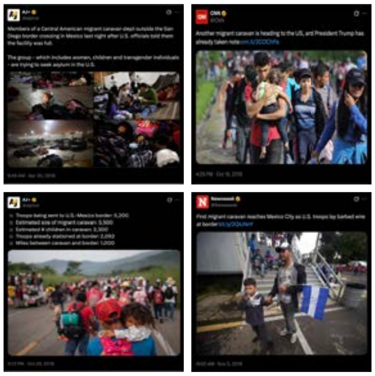

(f) Women and Children

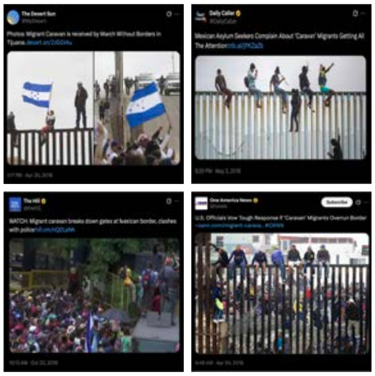

(g) Violations

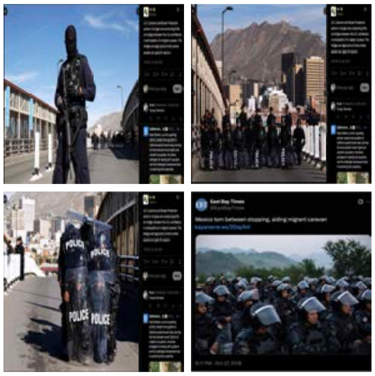

(h) Police

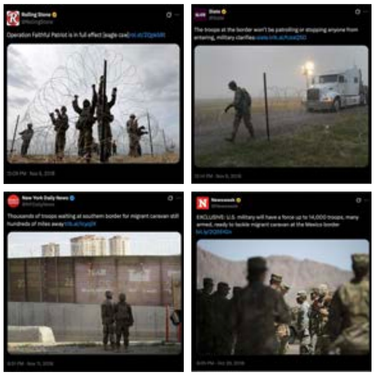

(i) Military
