# Supplementary material for: Media choice and audience perceptions: Evidence from visual framing of immigration in news stories
Source: PLoS One. 2025 Sep 15;20(9):e0331219. doi: 10.1371/journal.pone.0331219 (PMC12435698; doi:10.1371/journal.pone.0331219)
Supplement: S1 Appendix — (ZIP) [file pone.0331219.s001.zip › si_files/S21_Table.pdf]

Table S.21: Linear regressions for partisan subsamples on all clusters with "Crowds" as a baseline category.

	Dependent variable:	
	Is it a Liberal Outlet?	
	Democrats	Republicans
	(1)	(2)
Camps	0.142***	0.048
	(0.050)	(0.058)
Close Shots (Men)	0.078***	0.058*
	(0.029)	(0.031)
Close Shots (Women/Children)	0.114***	0.055**
	(0.021)	(0.024)
Crowds	[baseline category]	
Democratic Politicians	0.061	0.124
	(0.068)	(0.081)
Military	−0.099***	−0.196***
	(0.037)	(0.038)
Police	−0.076	−0.021
	(0.050)	(0.058)
Republican Politicians	−0.094***	−0.311***
	(0.029)	(0.031)
Violations	−0.037	−0.068*
	(0.034)	(0.038)
Age Category	0.016*	0.044***
	(0.008)	(0.009)
Gender	0.017	−0.015
	(0.029)	(0.034)
Education	−0.007	0.011
	(0.011)	(0.012)
Hispanic	0.037	0.058
	(0.045)	(0.071)
Income	−0.006	−0.010
	(0.006)	(0.007)
Interest in Politics	0.009	−0.017
	(0.013)	(0.015)
Constant	0.287***	0.373***
	(0.072)	(0.078)
Controls	✓	✓
Observations	3,153	2,247

Note: *p<0.1; **p<0.05; ***p<0.01. All regressions are linear models with image-level and respondent-level random effects. Standard errors are in parentheses.
