# Supplementary material for: Media choice and audience perceptions: Evidence from visual framing of immigration in news stories
Source: PLoS One. 2025 Sep 15;20(9):e0331219. doi: 10.1371/journal.pone.0331219 (PMC12435698; doi:10.1371/journal.pone.0331219)
Supplement: S1 Appendix — (ZIP) [file pone.0331219.s001.zip › si_files/S19_Table.pdf]

S12 Media Outlet Ideology Guess Results

Table S.19: Linear regressions for partisan subsamples on all clusters with "Violations" as a baseline category.

	Dependent variable:	
	Is it a Liberal Outlet?	
	Democrats	Republicans
	(1)	(2)
Camps	0.180***	0.117*
	(0.056)	(0.064)
Close Shots (Men)	0.115***	0.127***
	(0.038)	(0.042)
Close Shots (Women/Children)	0.152***	0.123***
	(0.032)	(0.037)
Crowds	0.037	0.068*
	(0.034)	(0.038)
Democratic Politicians	0.098	0.192**
	(0.073)	(0.086)
Military	−0.061	−0.128***
	(0.044)	(0.048)
Police	−0.039	0.047
	(0.055)	(0.065)
Republican Politicians	−0.057	−0.243***
	(0.038)	(0.042)
Violations	[baseline category]	
Age Category	0.016*	0.044***
	(0.008)	(0.009)
Gender	0.017	−0.015
	(0.029)	(0.034)
Education	−0.007	0.011
	(0.011)	(0.012)
Hispanic	0.037	0.058
	(0.045)	(0.071)
Income	−0.006	−0.010
	(0.006)	(0.007)
Interest in Politics	0.009	−0.017
	(0.013)	(0.015)
Constant	0.250***	0.305***
	(0.075)	(0.084)
Controls	✓	✓
Observations	3,153	2,247

Note: *p<0.1; **p<0.05; ***p<0.01. All regressions are linear models with image-level and respondent-level random effects. Standard errors are in parentheses.
