# Supplementary material for: Media choice and audience perceptions: Evidence from visual framing of immigration in news stories
Source: PLoS One. 2025 Sep 15;20(9):e0331219. doi: 10.1371/journal.pone.0331219 (PMC12435698; doi:10.1371/journal.pone.0331219)
Supplement: S1 Appendix — (ZIP) [file pone.0331219.s001.zip › si_files/S1_Appendix.pdf]

Media Choice and Audience Perceptions: Evidence from Visual Framing of Immigration in News Stories.
Online Appendix

Contents

S1 Media Outlets Descriptions **2**

S2 "Migrant Caravans" is Equally Present in Liberal and Conservative Media Outlets **8**

S3 Unsupervised Clustering **11**

S4 A Timeline of How Media Outlets Use Visual Frames **14**

S5 Robustness Checks **16**
- S5.1 Alternative Measure of Outlet Ideology 16
- S5.2 Alternative Search of Images 18
- S5.3 Only 2018 20

S6 Participants Sample Description Statistics **22**
- S6.1 Sample demographics vs. Population demographics 24

S7 Manipulation Check **25**

S8 Power Analysis Simulations **26**

S9 Descriptive Statistics for the Three Main Outcome Variables **27**

S10 ANOVA Results **28**

S11 Within-Party Analysis of All Visual Frames: Regression Results for Accuracy and Attitudes **29**

S12 Media Outlet Ideology Guess Results **33**

S13 Comparison of High- and Low-Confidence Responses for Accuracy and Outlet Ideology Guesses **36**

S14 Outlets Ideology and the Choice of Visual Frames **41**

S15 Correlations between Outlet Ideology and Outlet Ideology Guesses **42**

S16 Curated Labeling **43**

S17 Questionnaire **45**

S18 Curated Codebook and Labeling **53**

S1 Media Outlets Descriptions

To acquire media outlets list we scraped all media outlet entries from AllSides website (N=1044); we then subset only those media outlets that were labeled as "News Media" (N=437) to eliminate individual authors, candidate/campaign advocates, dialogues and debates, satire and human outlets, podcasts, opinion outlets, policy/think tank outlets, reference, and fact checking outlets. We then mapped Twitter user names to each of these outlets, which left us with 393 news outlets that had active (meaning that it had a valid username and was accessible through Twitter API) Twitter accounts. For each of these 393 media outlets we used All Sides label of ideological leaning of the outlet (captured at the point of December 2021), that was measured on a 5 point scale: Very Liberal (Left), Moderate Liberal (Lean Left), Moderate (Center), Moderate Conservative (Lean Right), Very Conservative (Right). The full list of these 393 outlets is displayed at Table S.1 split by an assigned ideological label according to AllSides.

Table S.1: List of media outlets.

Category	Items
**Very Liberal (Left)**	AJ+, AlterNet, Aquinas College Saint, Arkansas Democrat-Gazette, Block Club Chicago, Blue Virginia, Boing Boing, Brown Girl Magazine, BuzzFeed News, Care2, Chicago Crusader, Chicago Defender, CNN (Online News), CNN (Opinion), Common Dreams, Current Affairs, Daily Beast, Daily Chela, Daily Kos, Democracy Now, Esquire, Falls Church News - Press, Herald Democrat, Hip Latina, HuffPost, Jacobin, Jezebel, Latino Rebels, Leafly, LGBTQ Nation, Mashable, Media Matters, Metro Weekly, Mother Jones, MSNBC, New Republic, New York Daily News, New York Magazine, New York Times (Opinion), NewsOne, Peacock Panache, PinkNews, PoliticusUSA, Raw Story, Refinery29, RollingStone.com, Salon, San Francisco Chronicle, Slate, Socialist Alternative, Splinter, The Boston Globe, The Intercept, The Juggernaut, The Nation, The New Yorker, ThinkProgress, Truthdig, Upworthy, Vice, Vox, Yes! Magazine
**Moderate Liberal (Lean Left)**	ABC News (Online), Above The Law, Al Jazeera, Atlanta Black Star, Atlanta Journal-Constitution, Austin American-Statesman, BET, Black Enterprise, Bloomberg, Boston Review, Bowling Green Daily News, Bustle, CBS News (Online), Center for Public Integrity, Chicago Sun-Times, CNN Business, Counter Currents, Daily Northwestern, Daily Targum, Detroit Free Press, East Bay Times, Elle Magazine, ESPN.com, FAIR, Gizmodo, GLAAD, Google News, GQ.com, Grist, Harvard Business School, High Times, Indy Week, Las Vegas Sun, Longmont Times-Call, Los Angeles Times, Louisville Courier-Journal, Marijuana Moment, Mediaite, Miami Herald, Michigan Daily, MTV News Online, NBC News (Online), NBC Today Show, New York Times (News), Pacific Standard, Politico, ProPublica, San Diego Union-Tribune, San Jose Mercury News, Scientific American, SFGate, Sky-Hi Daily News, Spokesman Review, Star Tribune, State Journal, Teen Vogue, The Advocate, The Advocate-Messenger, The Atlantic, The Cadiz Record, The Colorado Sun, The Commercial Appeal, The Daily Dot, The Delaware County Daily Times, The Economist, The Guardian, The Hollywood Reporter, The Justice, The Maneater, The Onion (Humor), The Philadelphia Inquirer, The Root, The Sacramento Bee, The Texas Observer, The Texas Tribune, The Verge, The Week - News, Time Magazine, TruthOut, U.S. News & World Report, Univision, USA TODAY, Vanity Fair, VT Digger, Washington Monthly, Washington Post, Wisconsin Gazette, Yahoo News

**Moderate (Center)**	Arc Digital, Associated Press, Axios, AZ Central, Barnstable Patriot, BBC News, Bridgemi.com, C-SPAN, CalMatters, CalWatchdog, Chicago Tribune, Christian Science Monitor, CNBC, CNET, Columbia Journalism Review, Columbia Missourian, Concord Monitor, Cook Report, CrowdPAC, CU Independent, Daily Breeze, Daily Cardinal, Daily Progress, Defense One, Des Moines Register, Detroit News, Deutsche Welle, Diplomatic Courier, Duke Chronicle, Education Week, Erraticus, Estes Park Trail Gazette, Eurek Alert, Financial Times, FiveThirtyEight, Forbes, Foreign Affairs, Foreign Policy, Fort Worth Star-Telegram, Fortune, GoLocal Providence, Haaretz, Hampton Roads Messanger, Heavy.com, Honolulu Civil Beat, Houston Chronicle, Idaho Statesman, Independent Journal Review, Indiana Daily Student, Indy Online, IndyStar, Inland Valley Daily Bulletin, Inside Philanthropy, International Business Times, IVN, Jefferson Public Radio, Journalist's Resource, JSTOR Daily, Jubilee Media, Just Security, Just The News, KALW.org, KATU, Kenosha News, KQED, KWCH, Law & Crime, Lifehacker, Live Science, Long Beach Press-Telegram, Los Angeles Daily News, Making Sense, MarketWatch, McClatchyDC, Media Village, Military Times, Misinformation Review, MIT News, National Geographic, National Journal, Nature.com, Nautilus Quarterly, New Discourses, New Hampshire Union Leader, Newsweek, Newsy, Newtrals, Nieman Lab, NMPolitics.net, NPR (Online News), Our.News, Outkick the Coverage, Palm Springs Desert Sun, Pasadena Star-News, Patch.com, PBS NewsHour, Phys.org, Portland Press Herald, Poynter, PRI (Public Radio International), Psychology Today, Quartz, Rasmussen Reports, RealClearPolitics, Record Journal, Redlands Daily Facts, Reuters, Roll Call, San Antonio Express-News, San Bernardino Sun, San Gabriel Valley Tribune, Science Daily, SCOTUSblog, Scriberr Media - News, SF Weekly, Smerconish, Smithsonian Magazine, South China Morning Post, St. Louis Post-Dispatch, STAT, Tallahassee Democrat, Tampa Bay Times, Tech Xplore, TechCrunch, The Appeal, The Bellows, The Columbus Dispatch, The Dallas Morning News, The Fulcrum, The Globe and Mail, The Hill, The Japan Times, The Jerusalem Post, The Korea Herald, The Lufkin Daily News, The Markup, The Marshall Project, The Observer (New York), The Oracle, The Oregonian, The Red and Black, The Saturday Evening Post, The Seattle Times, The South African, The Tennesseean, The Times-Picayune, Times Union, Variety, Virginia Mercury, Voice of America, Volante, Wake Up to Politics, Wall Street Journal (News), WANDTV, WFAE, WGBH, WGN, Whittier Daily News, Wired
**Moderate Conservative (Lean Right)**	Babylon Bee (Humor), Boston Herald, Christianity Today, Daily Press, Deseret News, Desiring God, Drudge Report, Evie Magazine, Fiscal Times, Fox Business, HotAir, Investor's Business Daily, Judicial Watch, Leesburg Today, Live Action News, Meridian Magazine, New York Post (News), Newsmax (News), Orange County Register, Pittsburgh Post-Gazette, Project Veritas, Quillette, Reason, Richmond Times Dispatch, Spiked, Tablet Mag, The American Conservative, The American Mind, The Bulwark, The Christian Post, The Dispatch, The Epoch Times, The Libertarian Republic, The Police Tribune, The Post Millennial, The Press-Enterprise, The Telegraph - UK, The Texan, Wall Street Journal (Opinion), Washington Examiner, Washington Times, Watchdog.org, ZeroHedge
**Very Conservative (Right)**	American Greatness, American Thinker, Bearing Drift, Biblical Gender Roles, Bizpac Review, Breitbart News, Brownstone Institute, CBN, City Journal, CNSNews.com, Commentary Magazine, Daily Citizen, Daily Mail, Fox News (Online News), Fox News Latino, FrontPage Magazine, Inacow, KSL, Media Research Center, National Review, New York Post (Opinion), NewsBusters, One America News Network (OAN), PJ Media, RedState, Right Side News, The American Spectator, The College Fix, The Daily Caller, The Daily Signal, The Daily Wire, The Federalist, The Gateway Pundit, The Imaginative Conservative, The Resurgent, The Weekly Standard, The Western Journal, TheBlaze.com, Townhall, Washington Free Beacon, WND.com

Table S.2: Distribution of all pulled images across media outlets and years.

**Media Outlet/Year**	2017	2018	2019	2020	2021	**Total**
ABC News	0	26	0	0	0	26
AJ+	1	25	0	0	0	26
Al Jazeera English	0	13	1	5	10	29
American Greatness	0	1	0	0	0	1
Axios	0	2	0	0	0	2
azcentral	0	1	0	0	0	1
Block Club Chicago	0	2	0	0	0	2
Blue Lives Matter	0	4	0	0	0	4
Boston Herald	0	6	0	0	0	6
Breitbart News	0	1	0	0	0	1
BuzzFeed News	0	18	0	0	0	18
CBS News	0	53	9	0	0	62
Chicago Sun-Times	0	1	0	0	0	1
Chicago Tribune	0	19	0	0	0	19
CNN	0	58	3	0	0	61
CNN Business	0	1	0	0	0	1
Commercial Appeal	0	2	0	0	0	2
Conservative News	0	23	4	1	9	37
Courier Journal	0	3	0	0	0	3
Daily Caller	0	127	14	0	0	141
Daily Mail US	0	1	0	0	0	1
Daily Press	0	2	0	0	0	2
Daily Wire	0	21	0	4	4	29
Democracy Now!	0	11	0	0	0	11
DW News	0	1	0	0	0	1
East Bay Times	0	9	0	0	0	9
Esquire	0	0	1	0	0	1
Financial Times	0	1	0	0	0	1
FOX Business	0	4	0	0	0	4
Fox News	0	30	0	0	0	30
FrontPage Magazine	0	4	0	0	0	4
Gizmodo	0	2	0	0	0	2
GoLocalProv	0	1	0	0	0	1
GQ Magazine	0	3	0	0	0	3
heralddemocrat	0	1	0	0	0	1
HotAir.com	0	0	2	3	3	8
IndyStar	0	7	0	0	0	7
Jezebel	0	6	0	0	0	6
Judicial Watch	0	9	0	0	0	9
KATU News	0	5	0	0	0	5
KQED	0	4	1	0	0	5
L.A. Daily News	0	2	0	0	0	2
Las Vegas Sun	0	2	0	0	0	2
Law & Crime	0	4	0	0	0	4
LGBTQ Nation	0	7	0	0	0	7
Los Angeles Times	0	26	1	0	0	27
MarketWatch	0	5	0	0	0	5
Media Matters	0	9	3	0	4	16
Mediaite	0	17	0	0	0	17
MediaResearchCenter	0	10	1	0	0	11
Mercury News	0	4	0	0	0	4
Miami Herald	0	8	1	0	0	9
Military Times	0	3	0	0	0	3
Mother Jones	0	18	0	0	0	18
MSNBC	0	2	0	0	0	2
National Review	0	26	2	0	0	28
NBC News	0	13	0	0	0	13
New York Daily News	0	23	0	0	0	23
New York Post	0	25	1	2	14	42
New York Times Opinion	0	7	0	0	0	7

NewsBusters	0	11	1	0	0	12
Newsmax	0	2	0	1	0	3
Newsweek	0	122	2	0	0	124
NOLA.com	0	2	0	0	0	2
NPR	0	4	2	0	0	6
NY Post Opinion	0	3	0	0	0	3
Observer	0	1	0	0	0	1
One America News	0	16	9	3	2	30
PJ Media	0	1	0	0	0	1
POLITICO	0	6	0	0	0	6
PoliticusUSA	0	1	0	0	0	1
Rasmussen Reports	0	0	0	0	3	3
Record-Journal	0	1	0	0	0	1
Reuters	0	26	6	16	36	84
Rolling Stone	0	8	0	0	0	8
San Francisco Chronicle	0	2	1	0	0	3
SFGATE	0	7	0	0	0	7
Slate	0	57	0	0	0	57
South China Morning Post	0	1	0	0	0	1
Splinter	0	13	2	0	0	15
St. Louis Post-Dispatch	0	3	1	0	3	7
Star Tribune	0	8	1	0	0	9
Tennessean	0	1	0	0	0	1
Texas Tribune	0	9	12	0	0	21
The Babylon Bee	0	6	0	0	0	6
The Boston Globe	0	6	1	0	0	7
The Christian Science Monitor	0	1	0	0	0	1
The Daily Beast	0	8	0	0	0	8
The Daily Cardinal	0	1	0	0	0	1
The Daily Dot	0	9	2	0	0	11
The Daily Signal	0	8	1	0	0	9
The Desert Sun	0	16	0	0	0	16
The Detroit News	0	3	0	0	0	3
The Epoch Times	0	4	0	0	0	4
The Globe and Mail	0	16	0	0	0	16
The Guardian	0	1	0	0	0	1
The Hill	0	170	9	1	4	184
The Hollywood Reporter	0	5	0	0	0	5
The Japan Times	0	6	1	0	0	7
The Jerusalem Post	0	2	0	0	0	2
The New Republic	0	9	0	0	0	9
The New Yorker	0	23	0	0	0	23
The Oregonian	0	5	0	0	0	5
The Resurgent	0	4	0	0	0	4
The Root	0	1	0	0	0	1
The San Diego Union-Tribune	0	47	12	0	0	59
The Telegraph	0	1	0	0	0	1
The Verge	0	4	0	0	0	4
The Voice of America	0	6	0	0	0	6
The Washington Times	0	153	18	1	1	173
The Week	0	3	0	0	0	3
TheBlaze	0	3	0	0	0	3
ThinkProgress	0	76	0	0	0	76
TODAY	0	1	0	0	0	1
Truthout	0	3	0	0	0	3
U.S. News & World Report	0	12	1	0	0	13
UnionLeader.com	0	1	0	0	0	1
USA TODAY	0	13	0	0	0	13
Variety	0	1	0	0	0	1
Washington Examiner	0	29	0	0	0	29
WFAE	0	8	0	0	0	8
WGN TV News	0	4	0	0	0	4
Yahoo News	0	50	2	0	0	52

YES! Magazine	0	4	0	0	0	4

Table S.3: Distribution of images used in the survey wave across media outlets and years.

**Media Outlet/Year**	2017	2018	2019	2020	2021	**Total**
ABC News	0	1	0	0	0	1
AJ+	1	9	0	0	0	10
Al Jazeera English	0	2	1	2	2	7
American Greatness	0	1	0	0	0	1
Axios	0	1	0	0	0	1
azcentral	0	1	0	0	0	1
Boston Herald	0	1	0	0	0	1
CBS News	0	15	3	0	0	18
Chicago Tribune	0	5	0	0	0	5
CNN	0	7	0	0	0	7
Conservative News	0	2	1	1	2	6
Daily Caller	0	24	3	0	0	27
Daily Wire	0	3	0	1	2	6
Democracy Now!	0	5	0	0	0	5
DW News	0	1	0	0	0	1
East Bay Times	0	3	0	0	0	3
Fox News	0	1	0	0	0	1
FrontPage Magazine	0	1	0	0	0	1
Gizmodo	0	2	0	0	0	2
GoLocalProv	0	1	0	0	0	1
HotAir.com	0	0	0	0	1	1
IndyStar	0	2	0	0	0	2
KATU News	0	2	0	0	0	2
KQED	0	2	0	0	0	2
L.A. Daily News	0	1	0	0	0	1
Los Angeles Times	0	6	0	0	0	6
Mediaite	0	1	0	0	0	1
MediaResearchCenter	0	2	0	0	0	2
Military Times	0	2	0	0	0	2
Mother Jones	0	1	0	0	0	1
National Review	0	6	1	0	0	7
NBC News	0	5	0	0	0	5
New York Daily News	0	8	0	0	0	8
New York Post	0	6	1	0	3	10
Newsmax	0	1	0	0	0	1
Newsweek	0	31	0	0	0	31
NOLA.com	0	2	0	0	0	2
NY Post Opinion	0	2	0	0	0	2
One America News	0	8	4	1	0	13
PJ Media	0	1	0	0	0	1
POLITICO	0	1	0	0	0	1
PoliticusUSA	0	1	0	0	0	1
Reuters	0	5	2	6	10	23
Rolling Stone	0	2	0	0	0	2
San Francisco Chronicle	0	1	0	0	0	1
SFGATE	0	2	0	0	0	2
Slate	0	6	0	0	0	6
Splinter	0	1	0	0	0	1
St. Louis Post-Dispatch	0	2	0	0	1	3
Star Tribune	0	4	1	0	0	5
Texas Tribune	0	2	1	0	0	3
The Boston Globe	0	2	1	0	0	3
The Daily Beast	0	1	0	0	0	1
The Daily Signal	0	2	0	0	0	2
The Desert Sun	0	9	0	0	0	9

The Globe and Mail	0	6	0	0	0	6
The Guardian	0	1	0	0	0	1
The Hill	0	19	1	0	1	21
The Hollywood Reporter	0	1	0	0	0	1
The Japan Times	0	1	0	0	0	1
The New Republic	0	1	0	0	0	1
The Oregonian	0	3	0	0	0	3
The San Diego Union-Tribune	0	5	3	0	0	8
The Voice of America	0	4	0	0	0	4
The Washington Times	0	14	2	0	0	16
ThinkProgress	0	1	0	0	0	1
Truthout	0	2	0	0	0	2
U.S. News & World Report	0	3	0	0	0	3
USA TODAY	0	1	0	0	0	1
Washington Examiner	0	2	0	0	0	2
WGN TV News	0	1	0	0	0	1
Yahoo News	0	9	1	0	0	10
YES! Magazine	0	1	0	0	0	1

S2 "Migrant Caravans" is Equally Present in Liberal and Conservative Media Outlets

It is possible that Republicans use the expression ″migrant caravan″ more frequently than Democrats, so the word choice we have made might have influenced the structure of the image sample we use. By searching social media content with only one textual connotation, some information may not be retrieved. We show in Figure S.1 that this intuition is not supported. The term ″migrant caravan″ is equally frequently used by left- and right-leaning media outlets, so our main predictor has no effect on it. In Figure S.2 it is shown that left-leaning and right-leaning media outlets are equally likely to attach images to tweets, indicating that there is no systematic bias in the frequency with which left- and right-leaning media use the term ″migrant caravans″ to discuss immigration, and these media are equally likely to attach images to these stories.
