# Supplementary material for: Media choice and audience perceptions: Evidence from visual framing of immigration in news stories
Source: PLoS One. 2025 Sep 15;20(9):e0331219. doi: 10.1371/journal.pone.0331219 (PMC12435698; doi:10.1371/journal.pone.0331219)
Supplement: S1 Appendix — (ZIP) [file pone.0331219.s001.zip › si_files/S6_Appendix.pdf]

S6 Participants Sample Description Statistics

Table S.6: Descriptive statistics for survey respondents.

	N	Mean	St. Dev.	Min	Pct(25)	Pct(75)	Max
**N of participants**	2089						
**Age (by age group)**	2089	4.80	1.83	2.00	3.00	6.00	9.00
1- Under 18							
2- 18 - 24							
3- 25 - 34							
4- 35 - 44							
5- 45 - 54							
6- 55 - 64							
7- 65 - 74							
8- 75 - 84							
9- 85 or older							
**Gender**	2089	0.59	0.49	0.00	0.00	1.00	1.00
0-Male							
1-Female							
**Education**	2067	3.72	1.56	1.00	2.00	5.00	7.00
1-Less than high school							
2-High school graduate							
3-Some college but no degree							
4-Associate's degree in college (2-year)							
5-Bachelor's degree in college (4-year)							
6-Master's degree							
7-Doctoral degree							
**Hispanic Ethnicity**	2074	0.09	0.28	0.00	0.00	0.00	1.00
0-Not Hispanic							
1-Hispanic							
**Income**	2085	5.37	2.99	1.00	3.00	8.00	10.00
1-Less than $10,000							
2-$10,000 - $19,999							
3-$20,000 - $29,999							
4-$30,000 - $39,999							
5-$40,000 - $49,999							
6-$50,000 - $59,999							
7-$60,000 - $69,999							
8-$70,000 - $89,999							
9-$90,000 - $109,999							
10-More than $110,000							
**Interest**	1989	3.72	1.27	1.00	3.00	5.00	5.00
1-Not interested at all							
5-Very interested							
**Ideology**	2087	3.72	1.85	1.00	2.00	5.00	7.00
1-Very Conservative							
7-Very Liberal							
**Partisanship (3 point scale)**	2089	1.94	0.83	1.00	1.00	3.00	3.00
1-Democrat							
2-Republican							
3-Independent							
**Respondent's Perception of Accuracy**	1590	4.53	1.65	1.00	4.00	6.00	7.00
1-Faulty							
4-Middle Ground							
7-Accurate							
**Respondent's Attitudes**	1900	3.99	1.86	1.00	3.00	5.00	7.00
1-Extremely Negative							
4-Middle Ground							
7-Extremely Positive							
**Respondent's Guess of Outlet Ideology**	1524	4.01	1.90	1.00	3.00	5.00	7.00
1-Liberal							

4-Moderate							
7-Conservative							

Note: Descriptive statistics are provided for all respondents. Age is a categorical variable structured based on age ranges.

Table S.7: Descriptive statistics for survey respondents (excluding participants with self-reported partisanship as Independent).

	N	Mean	St. Dev.	Min	Pct(25)	Pct(75)	Max
**N of participants**	1425						
**Age (by age group)**	1425	4.85	1.81	2.00	3.00	6.00	9.00
1- Under 18							
2- 18 - 24							
3- 25 - 34							
4- 35 - 44							
5- 45 - 54							
6- 55 - 64							
7- 65 - 74							
8- 75 - 84							
9- 85 or older							
**Gender**	1425	0.59	0.49	0.00	0.00	1.00	1.00
0-Male							
1-Female							
**Education**	1410	3.83	1.56	1.00	2.00	5.00	7.00
1-Less than high school							
2-High school graduate							
3-Some college but no degree							
4-Associate's degree in college (2-year)							
5-Bachelor's degree in college (4-year)							
6-Master's degree							
7-Doctoral degree							
**Hispanic Ethnicity**	1417	0.08	0.27	0.00	0.00	0.00	1.00
0-Not Hispanic							
1-Hispanic							
**Income**	1421	5.62	2.97	1.00	3.00	8.00	10.00
1-Less than $10,000							
2-$10,000 - $19,999							
3-$20,000 - $29,999							
4-$30,000 - $39,999							
5-$40,000 - $49,999							
6-$50,000 - $59,999							
7-$60,000 - $69,999							
8-$70,000 - $89,999							
9-$90,000 - $109,999							
10-More than $110,000							
**Interest**	1364	3.90	1.18	1.00	4.00	5.00	5.00
1-Not interested at all							
5-Very interested							
**Ideology**	1425	3.68	2.04	1.00	2.00	5.00	7.00
1-Very Conservative							
7-Very Liberal							
**Partisanship (binary)**	1425	1.44	0.50	1.00	1.00	2.00	2.00
1-Democrat							
2-Republican							
**Respondent's Perception of Accuracy**	1120	4.58	1.64	1.00	4.00	6.00	7.00
1-Faulty							

4-Middle Ground							
7-Accurate							
**Respondent's Attitudes**	1320	4.04	1.88	1.00	3.00	5.00	7.00
1-Extremely Negative							
4-Middle Ground							
7-Extremely Positive							
**Respondent's Guess of Outlet Ideology**	1081	4.02	1.92	1.00	3.00	5.00	7.00
1-Liberal							
4-Moderate							
7-Conservative							

Note: Descriptive statistics are provided for all respondents. Age is a categorical variable structured based on age ranges.

S6.1 Sample demographics vs. Population demographics

Tables S.8, S.9, and S.10 present the distribution of sample respondents by demographic characteristics (gender, age, and ethnicity) in comparison to the estimates of general population. The general population distributions are derived from the Census Bureau's American Community Survey (ACS) Public Use Microdata Sample (PUMS) based on person files, built based on a five-year period (2019-2013) and reported in 2023 (https://www2.census.gov/programs-surveys/acs/data/pums/). We can observe similar distribution patterns across demographic groups. The observed differences are in gender and several age groups. Our sample has a larger proportion of females and slightly over representing population of age groups "25-34" and "35-44."

Table S.8: Distribution by gender.

	Census Freq	Survey Freq
Male	0.49	0.41
Female	0.51	0.59

Table S.9: Distribution by age.

	Census Freq	Survey Freq
18 - 24	0.106	0.107
25 - 34	0.147	0.166
35 - 44	0.148	0.199
45 - 54	0.149	0.129
55 - 64	0.178	0.174
65 - 74	0.159	0.157
75 - 84	0.083	0.060
85 and older	0.031	0.008

Table S.10: Distribution by Hispanic ethnicity.

	Census Freq	Survey Freq
Not Hispanic	0.86	0.92
Hispanic	0.14	0.08

S7 Manipulation Check

Table S.11: Results of multinomial logistic regression for the randomization check.

	Dependent variable:							
	Camps	Close Shots (Men)	Crowds	Democratic Politicians	Military	Police	Republican Politicians	Violations
	(1)	(2)	(3)	(4)	(5)	(6)	(7)	(8)
Party (Binary)	−0.044	−0.049	−0.092	0.239	−0.058	−0.015	0.038	0.003
	(0.128)	(0.070)	(0.056)	(0.197)	(0.092)	(0.133)	(0.075)	(0.088)
Age	−0.002	0.005	−0.009	−0.057	0.022	0.003	0.017	−0.016
	(0.036)	(0.020)	(0.016)	(0.054)	(0.026)	(0.037)	(0.021)	(0.025)
Gender	0.107	0.002	0.052	−0.064	0.084	−0.065	−0.004	−0.033
	(0.130)	(0.072)	(0.058)	(0.198)	(0.094)	(0.136)	(0.077)	(0.090)
Education	0.017	−0.045*	−0.018	0.051	0.001	0.055	−0.031	0.001
	(0.047)	(0.026)	(0.021)	(0.071)	(0.034)	(0.049)	(0.028)	(0.032)
Hispanic	0.539*	0.044	0.091	0.226	−0.090	−0.013	−0.058	0.109
	(0.286)	(0.130)	(0.104)	(0.360)	(0.164)	(0.238)	(0.134)	(0.163)
Income	0.014	0.030**	0.004	−0.022	0.0001	−0.034	−0.015	−0.002
	(0.025)	(0.014)	(0.011)	(0.037)	(0.018)	(0.026)	(0.015)	(0.017)
Interest in Politics	−0.022	−0.037	0.003	0.072	−0.023	0.073	−0.004	0.021
	(0.056)	(0.030)	(0.025)	(0.088)	(0.040)	(0.061)	(0.033)	(0.039)
Constant	−3.024***	−0.936***	−0.343**	−3.722***	−1.657***	−2.812***	−1.053***	−1.679***
	(0.376)	(0.185)	(0.149)	(0.527)	(0.240)	(0.356)	(0.196)	(0.235)
AIC	35,262.800	35,262.800	35,262.800	35,262.800	35,262.800	35,262.800	35,262.800	35,262.800

Note: *p<0.1; **p<0.05; ***p<0.01. Each column presents the results for a given image cluster in comparison with the reference category cluster category - ”Close Shots (Women/Children)”.
