# Supplementary material for: Media choice and audience perceptions: Evidence from visual framing of immigration in news stories
Source: PLoS One. 2025 Sep 15;20(9):e0331219. doi: 10.1371/journal.pone.0331219 (PMC12435698; doi:10.1371/journal.pone.0331219)
Supplement: S1 Appendix — (ZIP) [file pone.0331219.s001.zip › si_files/S10_Appendix.pdf]

S10 ANOVA Results

In this section, we report the results of ANOVA tests conducted for each outcome variable to examine whether there were significant differences in evaluations between Democrats and Republicans across image frames originating from media outlets with different ideological leanings. The analysis includes nine visual frame categories, two respondent partisanship groups (Democrats and Republicans), and three outlet ideology categories (Liberal, Moderate, Conservative).

We present the results for three key outcomes: accuracy (Table S.13), attitude (Table S.14), and media outlet ideology guess (Table S.15). Across all three outcomes, we observe statistically significant differences in how partisans evaluate different visual frames (Image Frame * Partisanship) interaction. However, we do not find statistically significant differences in how partisans evaluate images from the outlets with different ideological leanings (Outlet Ideology * Partisanship interaction).

For the accuracy and attitude outcomes, we also do not observe significant three-way interactions (Outlet Ideology * Image Frame * Partisanship), indicating that the combination of outlet ideology and visual frame does not significantly influence partisan evaluations on these measures. In contrast, for the outcome related to guessing the outlet's ideology, we do find a significant three-way interaction (Table S.15; $F = 2.04$, $p = 0.01$), suggesting that partisanship shapes how respondents infer outlet ideology based on both the visual frame and the outlet's ideological stance.

Table S.13: ANOVA results: Accuracy.

	Df	Sum Sq	Mean Sq	F value	Pr(>F)
Outlet Ideology	2.00	10.77	5.39	1.95	0.14
Image Frame	8.00	97.19	12.15	4.39	0.00
Partisanship	1.00	50.12	50.12	18.13	0.00
Outlet Ideology*Image Frame	15.00	69.14	4.61	1.67	0.05
Outlet Ideology*Partisanship	2.00	6.51	3.25	1.18	0.31
Image Frame*Partisanship	8.00	128.33	16.04	5.80	0.00
Outlet Ideology*Image Frame*Partisanship	15.00	42.99	2.87	1.04	0.41

Table S.14: ANOVA results: Attitude.

	Df	Sum Sq	Mean Sq	F value	Pr(>F)
Outlet Ideology	2.00	99.41	49.70	13.51	0.00
Image Frame	8.00	646.22	80.78	21.96	0.00
Partisanship	1.00	174.74	174.74	47.50	0.00
Outlet Ideology*Image Frame	15.00	236.60	15.77	4.29	0.00
Outlet Ideology*Partisanship	2.00	2.79	1.39	0.38	0.68
Image Frame*Partisanship	8.00	1783.38	222.92	60.60	0.00
Outlet Ideology*Image Frame*Partisanship	15.00	68.99	4.60	1.25	0.23

Table S.15: ANOVA results: Ideology.

	Df	Sum Sq	Mean Sq	F value	Pr(>F)
Outlet Ideology	2.00	3.19	1.59	6.91	0.00
Image Frame	8.00	49.93	6.24	27.08	0.00
Partisanship	1.00	14.20	14.20	61.61	0.00
Outlet Ideology*Image Frame	15.00	3.98	0.27	1.15	0.30
Outlet Ideology*Partisanship	2.00	0.56	0.28	1.22	0.30
Image Frame*Partisanship	8.00	7.96	1.00	4.32	0.00
Outlet Ideology*Image Frame*Partisanship	15.00	7.05	0.47	2.04	0.01

S11 Within-Party Analysis of All Visual Frames: Regression Results for Accuracy and Attitudes
