# Supplementary material for: Media choice and audience perceptions: Evidence from visual framing of immigration in news stories
Source: PLoS One. 2025 Sep 15;20(9):e0331219. doi: 10.1371/journal.pone.0331219 (PMC12435698; doi:10.1371/journal.pone.0331219)
Supplement: S1 Appendix — (ZIP) [file pone.0331219.s001.zip › si_files/S17_Appendix.pdf]

Table S.27: Curated labels distribution in the survey wave.

Curated Label	Number of Images
Camps	9
Close Shots (Men)	39
Close Shots (Women/Children)	108
Crowds	78
Democratic Politicians	4
Military	20
Police	8
Republican Politicians	32
Violations	21

S17 Questionnaire

Demographics I

1. What is your current age?

   - Under 18 (excluded)
   - 18 - 24
   - 25 - 34
   - 35 - 44
   - 45 - 54
   - 55 - 64
   - 65 - 74
   - 75 - 84
   - 85 or older

2. In which state of the United States do you live? [Drop-down list with all the US states]

3. How would you describe your gender?

   - Female
   - Male
   - Other

4. Please check one or more categories below to indicate what race(s) you consider yourself to be

   - White

- Black or African American
- American Indian or Alaska Native
- Asian/Pacific Islander
- Multi-racial
- Other

5. Are you of Spanish or Hispanic origin or descent?
    - Yes
    - No
    - Don't know
6. What is the highest level of school you have completed or the highest degree you have received?
    - Less than high school
    - High school graduate (high school diploma or equivalent including GED)
    - Some college but no degree
    - Associate's degree in college (2-year)
    - Bachelor's degree in college (4-year)
    - Master's degree
    - Doctoral degree

Attention Check I

Imagine you win $\$1,000,000$ in a lottery. And you are supposed to spend this money in one month. How would you spend it? If you don't want to spend it please explain why.

Please make sure to write down **at least two sentences**.

Demographics II

7. Please indicate your annual household income
    - Less than $10,000
    - $10,000 - $19,999
    - $20,000 - $29,999
    - $30,000 - $39,999

- $40,000 - $49,999
- $50,000 - $59,999
- $60,000 - $69,999
- $70,000 - $89,999
- $90,000 - $109,999
- $110,000 or more

Attention Check II

What are your favorite ice cream flavors?

Recent research on decision making shows that choices are affected by context. Differences in how people feel, their previous knowledge and experience, and their environment can affect choices. To help us understand how people make decisions, we are interested in information about you. Specifically, we are interested in whether you actually take the time to read the directions. To show this, please ignore the question below about how your real favorite ice cream flavors, and instead check cookie dough, neapolitan, and peanut butter swirl options as your answer. Thank you very much.

Please check all words that describe your favorite ice cream flavors.

- peanut butter swirl (1 score point)
- chocolate mint
- neapolitan (1 score point)
- cookie dough (1 score point)
- chocolate
- vanilla

Scoring: 3 points total with 1 point for each correct answer.

Political Attitudes

8. How interested are you in what is going on in the government and politics?
    - Not interested at all
    - Somewhat not interested
    - Indifferent
    - Somewhat interested
    - Strongly interested

9. Generally speaking, do you usually think of yourself as a DEMOCRAT, a REPUBLICAN, an INDEPENDENT, or other?
    - Democrat
    - Republican
    - Independent
    - Other party (specify)
10. In the survey flow, if DEMOCRAT: Would you call yourself a STRONG Democrat or a NOT VERY STRONG Democrat?
    - Strong Democrat
    - Not very strong Democrat
11. In the survey flow, if REPUBLICAN: Would you call yourself a STRONG Republican or a NOT VERY STRONG Republican?
    - Strong Republican
    - Not very strong Republican
12. In the survey flow, if INDEPENDENT: Do you think of yourself as CLOSER to the Republican Party or the Democratic Party?
    - Closer to Republican Party
    - Closer to Democratic party
    - Neither

Disclaimer I

We will now ask you a few questions about your political preferences and opinions on certain issues.

13. We'd like you to rate how you feel towards both Democrats and Republicans on a scale of 0 to 100, which we call a "feeling thermometer." On this feeling thermometer scale, ratings between 0 and 49 degrees mean that you feel unfavorable and cold (with 0 being the most unfavorable/coldest). Ratings between 51 and 100 degrees mean that you feel favorable and warm (with 100 being the most favorable/warmest). A rating of 50 means you have no feelings one way or the other.

    Respondents have to move the thermometer roller on the relevant for them level for 1. Democrats; 2. Republicans
14. When it comes to politics, would you describe yourself as liberal, conservative, or neither liberal nor conservative?

- Very conservative
- Somewhat conservative
- Slightly conservative
- Moderate; middle of the road
- Slightly liberal
- Somewhat liberal
- Very liberal

15. Some people believe that immigrants are entitled to live here while retaining their own culture. Others feel that they should adapt entirely to the American culture. Where would you place yourself on a scale from 1 to 5, where 1 means that immigrants can retain their own culture and 5 means that they should adapt entirely?

    - 1. immigrants can retain their own culture
    - 2
    - 3
    - 4
    - 5 they should adapt entirely

Attention Check III

The previous question asked you about ....

- Trust and distrust toward different sources of information (correct answer)
- Your opinion about whether you support or oppose gay marriage
- Which media sources you prefer for getting political information
- Whether you are interested in politics

Attention Check Filter: Participants who scored fewer than three points on Attention Check II and answered this single-choice question incorrectly were excluded.

Disclaimer II

We will now show you a series of images that were used in different news stories about immigration. We ask you to answer a few questions regarding your perceptions of each of those photos.

Images Evaluation

[Each Respondent randomly receives 10 images. Each image evaluation has the same structure and is presented in the following way]

Look at the following image

[IMAGE IS PRESENTED HERE]

16. Would you say that this image portrays the subject(s) in this picture in a positive or negative light? ′1′ stands for negative, ′4′ is the middle ground (neutral), and ′7′ stands for positive.
    - 1 - negative
    - 2
    - 3
    - 4 - neutral
    - 5
    - 6
    - 7 - positive
    - Don′t know/ Not applicable
17. Do you think that this image is a faulty or accurate representation of the story that actually occurred? ′1′ stands for faulty, ′4′ is the middle ground, ′7′ stands for accurate.
    - 1 - faulty
    - 2
    - 3
    - 4 - neutral
    - 5
    - 6
    - 7 - accurate
    - Don′t know/ Not applicable
18. How confident are you in your opinion?
    - 1 - not confident at all
    - 2
    - 3
    - 4 - neutral

- 5
- 6
- 7 - very confident
- Don’t know/ Not applicable

19. Do you think that this image is from a liberal or conservative media outlet? ’1’ stands for liberal, ’4’ is moderate, and ’7’ stands for conservative.

    - 1 - liberal
    - 2
    - 3
    - 4 - moderate
    - 5
    - 6
    - 7 - conservative
    - Don’t know/ Not applicable

20. How confident are you in your opinion?

    - 1 - not confident at all
    - 2
    - 3
    - 4 - neutral
    - 5
    - 6
    - 7 - very confident
    - Don’t know/ Not applicable

Distraction I

In your opinion, which of the following movies do you think earned the most in the U.S. in 2021?

- Spider-Man: No Way Home
- No time to die
- Cruella
- Black Widow

Attention Check IV

Before we proceed, we have a question about how you are feeling.

To answer this question accurately, take a moment to assess your current mood. Please ignore the question below about how you are feeling, and instead check both the ″calm″ and ″jittery″ options as your answer. Thank you very much.

Please check all words that describe how you are currently feeling.

- Interested
- Distressed
- Excited
- Upset
- Strong
- Calm
- Guilty
- Scared
- Hostile
- Proud
- Enthusiastic
- Irritable
- Alert
- Ashamed
- Inspired
- Happy
- Nervous
- Determined
- Attentive
- Jittery
- Active
- Afraid
- None of the above

Closing Validity Checks

21. What year were you born?
22. What is your zip code?
