# Supplementary material for: Media choice and audience perceptions: Evidence from visual framing of immigration in news stories
Source: PLoS One. 2025 Sep 15;20(9):e0331219. doi: 10.1371/journal.pone.0331219 (PMC12435698; doi:10.1371/journal.pone.0331219)
Supplement: S1 Appendix — (ZIP) [file pone.0331219.s001.zip › si_files/S6_Table.pdf]

S6 Participants Sample Description Statistics

Table S.6: Descriptive statistics for survey respondents.

	N	Mean	St. Dev.	Min	Pct(25)	Pct(75)	Max
**N of participants**	2089						
**Age (by age group)**	2089	4.80	1.83	2.00	3.00	6.00	9.00
1- Under 18							
2- 18 - 24							
3- 25 - 34							
4- 35 - 44							
5- 45 - 54							
6- 55 - 64							
7- 65 - 74							
8- 75 - 84							
9- 85 or older							
**Gender**	2089	0.59	0.49	0.00	0.00	1.00	1.00
0-Male							
1-Female							
**Education**	2067	3.72	1.56	1.00	2.00	5.00	7.00
1-Less than high school							
2-High school graduate							
3-Some college but no degree							
4-Associate's degree in college (2-year)							
5-Bachelor's degree in college (4-year)							
6-Master's degree							
7-Doctoral degree							
**Hispanic Ethnicity**	2074	0.09	0.28	0.00	0.00	0.00	1.00
0-Not Hispanic							
1-Hispanic							
**Income**	2085	5.37	2.99	1.00	3.00	8.00	10.00
1-Less than $10,000							
2-$10,000 - $19,999							
3-$20,000 - $29,999							
4-$30,000 - $39,999							
5-$40,000 - $49,999							
6-$50,000 - $59,999							
7-$60,000 - $69,999							
8-$70,000 - $89,999							
9-$90,000 - $109,999							
10-More than $110,000							
**Interest**	1989	3.72	1.27	1.00	3.00	5.00	5.00
1-Not interested at all							
5-Very interested							
**Ideology**	2087	3.72	1.85	1.00	2.00	5.00	7.00
1-Very Conservative							
7-Very Liberal							
**Partisanship (3 point scale)**	2089	1.94	0.83	1.00	1.00	3.00	3.00
1-Democrat							
2-Republican							
3-Independent							
**Respondent's Perception of Accuracy**	1590	4.53	1.65	1.00	4.00	6.00	7.00
1-Faulty							
4-Middle Ground							
7-Accurate							
**Respondent's Attitudes**	1900	3.99	1.86	1.00	3.00	5.00	7.00
1-Extremely Negative							
4-Middle Ground							
7-Extremely Positive							
**Respondent's Guess of Outlet Ideology**	1524	4.01	1.90	1.00	3.00	5.00	7.00
1-Liberal							

4-Moderate							
7-Conservative							

Note: Descriptive statistics are provided for all respondents. Age is a categorical variable structured based on age ranges.

Table S.7: Descriptive statistics for survey respondents (excluding participants with self-reported partisanship as Independent).

	N	Mean	St. Dev.	Min	Pct(25)	Pct(75)	Max
**N of participants**	1425						
**Age (by age group)**	1425	4.85	1.81	2.00	3.00	6.00	9.00
1- Under 18							
2- 18 - 24							
3- 25 - 34							
4- 35 - 44							
5- 45 - 54							
6- 55 - 64							
7- 65 - 74							
8- 75 - 84							
9- 85 or older							
**Gender**	1425	0.59	0.49	0.00	0.00	1.00	1.00
0-Male							
1-Female							
**Education**	1410	3.83	1.56	1.00	2.00	5.00	7.00
1-Less than high school							
2-High school graduate							
3-Some college but no degree							
4-Associate's degree in college (2-year)							
5-Bachelor's degree in college (4-year)							
6-Master's degree							
7-Doctoral degree							
**Hispanic Ethnicity**	1417	0.08	0.27	0.00	0.00	0.00	1.00
0-Not Hispanic							
1-Hispanic							
**Income**	1421	5.62	2.97	1.00	3.00	8.00	10.00
1-Less than $10,000							
2-$10,000 - $19,999							
3-$20,000 - $29,999							
4-$30,000 - $39,999							
5-$40,000 - $49,999							
6-$50,000 - $59,999							
7-$60,000 - $69,999							
8-$70,000 - $89,999							
9-$90,000 - $109,999							
10-More than $110,000							
**Interest**	1364	3.90	1.18	1.00	4.00	5.00	5.00
1-Not interested at all							
5-Very interested							
**Ideology**	1425	3.68	2.04	1.00	2.00	5.00	7.00
1-Very Conservative							
7-Very Liberal							
**Partisanship (binary)**	1425	1.44	0.50	1.00	1.00	2.00	2.00
1-Democrat							
2-Republican							
**Respondent's Perception of Accuracy**	1120	4.58	1.64	1.00	4.00	6.00	7.00
1-Faulty							
