# Supplementary material for: Media choice and audience perceptions: Evidence from visual framing of immigration in news stories
Source: PLoS One. 2025 Sep 15;20(9):e0331219. doi: 10.1371/journal.pone.0331219 (PMC12435698; doi:10.1371/journal.pone.0331219)
Supplement: S1 Appendix — (ZIP) [file pone.0331219.s001.zip › si_files/S27_Table.pdf]

Table S.27: **Curated labels distribution in the survey wave.**

Curated Label	Number of Images
Camps	9
Close Shots (Men)	39
Close Shots (Women/Children)	108
Crowds	78
Democratic Politicians	4
Military	20
Police	8
Republican Politicians	32
Violations	21

S17 Questionnaire

Demographics I

1. What is your current age?
   - Under 18 (excluded)
   - 18 - 24
   - 25 - 34
   - 35 - 44
   - 45 - 54
   - 55 - 64
   - 65 - 74
   - 75 - 84
   - 85 or older
2. In which state of the United States do you live? [Drop-down list with all the US states]
3. How would you describe your gender?
   - Female
   - Male
   - Other
4. Please check one or more categories below to indicate what race(s) you consider yourself to be
   - White
