# Supplementary material for: Media choice and audience perceptions: Evidence from visual framing of immigration in news stories
Source: PLoS One. 2025 Sep 15;20(9):e0331219. doi: 10.1371/journal.pone.0331219 (PMC12435698; doi:10.1371/journal.pone.0331219)
Supplement: S1 Appendix — (ZIP) [file pone.0331219.s001.zip › si_files/S12_Table.pdf]

S9 Descriptive Statistics for the Three Main Outcome Variables

Table S.12: Descriptive statistics by visual frame and partisan groups.

	Accuracy		Attitude		Ideology	
Visual Frame	Dem	Rep	Dem	Rep	Dem	Rep
Camps	$\mu = 5.20$ ($\sigma = 1.66$)	$\mu = 4.82$ ($\sigma = 1.72$)	$\mu = 3.74$ ($\sigma = 2.14$)	$\mu = 3.71$ ($\sigma = 1.96$)	$\mu = 3.90$ ($\sigma = 1.88$)	$\mu = 3.71$ ($\sigma = 1.72$)
Close Shots (Men)	$\mu = 4.95$ ($\sigma = 1.52$)	$\mu = 4.71$ ($\sigma = 1.60$)	$\mu = 4.43$ ($\sigma = 1.85$)	$\mu = 3.76$ ($\sigma = 1.82$)	$\mu = 4.12$ ($\sigma = 1.77$)	$\mu = 3.77$ ($\sigma = 1.86$)
Close Shots (Women/Children)	$\mu = 5.09$ ($\sigma = 1.60$)	$\mu = 4.73$ ($\sigma = 1.68$)	$\mu = 4.53$ ($\sigma = 1.92$)	$\mu = 3.98$ ($\sigma = 1.86$)	$\mu = 4.04$ ($\sigma = 1.81$)	$\mu = 3.71$ ($\sigma = 1.80$)
Crowds	$\mu = 4.98$ ($\sigma = 1.58$)	$\mu = 4.82$ ($\sigma = 1.73$)	$\mu = 4.37$ ($\sigma = 1.90$)	$\mu = 3.53$ ($\sigma = 1.86$)	$\mu = 4.48$ ($\sigma = 1.76$)	$\mu = 3.88$ ($\sigma = 1.84$)
Democratic Politicians	$\mu = 5.40$ ($\sigma = 1.57$)	$\mu = 4.69$ ($\sigma = 1.87$)	$\mu = 4.91$ ($\sigma = 1.96$)	$\mu = 3.40$ ($\sigma = 1.88$)	$\mu = 4.17$ ($\sigma = 2.08$)	$\mu = 3.03$ ($\sigma = 1.80$)
Military	$\mu = 5.03$ ($\sigma = 1.57$)	$\mu = 4.97$ ($\sigma = 1.78$)	$\mu = 4.10$ ($\sigma = 2.03$)	$\mu = 4.50$ ($\sigma = 2.09$)	$\mu = 4.68$ ($\sigma = 1.78$)	$\mu = 4.65$ ($\sigma = 1.90$)
Police	$\mu = 4.89$ ($\sigma = 1.53$)	$\mu = 5.09$ ($\sigma = 1.49$)	$\mu = 3.22$ ($\sigma = 1.98$)	$\mu = 4.00$ ($\sigma = 2.13$)	$\mu = 4.70$ ($\sigma = 1.82$)	$\mu = 3.84$ ($\sigma = 1.97$)
Republican Politicians	$\mu = 5.05$ ($\sigma = 1.97$)	$\mu = 5.45$ ($\sigma = 1.72$)	$\mu = 3.15$ ($\sigma = 2.11$)	$\mu = 5.17$ ($\sigma = 1.91$)	$\mu = 4.91$ ($\sigma = 1.99$)	$\mu = 5.24$ ($\sigma = 1.93$)
Violations	$\mu = 4.81$ ($\sigma = 1.72$)	$\mu = 4.93$ ($\sigma = 1.86$)	$\mu = 3.58$ ($\sigma = 1.99$)	$\mu = 2.91$ ($\sigma = 1.90$)	$\mu = 4.54$ ($\sigma = 1.84$)	$\mu = 4.05$ ($\sigma = 1.99$)

Note: Partisanship was self-reported (1 = Democrat; 0 = Republican). Accuracy and attitude were measured on 7-point scales ranging from 1 (image gives a faulty representation/evokes a negative attitude) to 7 (image gives an accurate representation/evokes a positive attitude). Media outlet ideology guess was measured on a 7-point scale ranging from 1 (image is from a liberal media outlet) to 7 (image is from a conservative media outlet).
