# Supplementary material for: Media choice and audience perceptions: Evidence from visual framing of immigration in news stories
Source: PLoS One. 2025 Sep 15;20(9):e0331219. doi: 10.1371/journal.pone.0331219 (PMC12435698; doi:10.1371/journal.pone.0331219)
Supplement: S1 Appendix — (ZIP) [file pone.0331219.s001.zip › si_files/S7_Fig.pdf]

Fig. S.7: Visual frames that dominated right-leaning media reports about migrant caravans from October to November 2018.

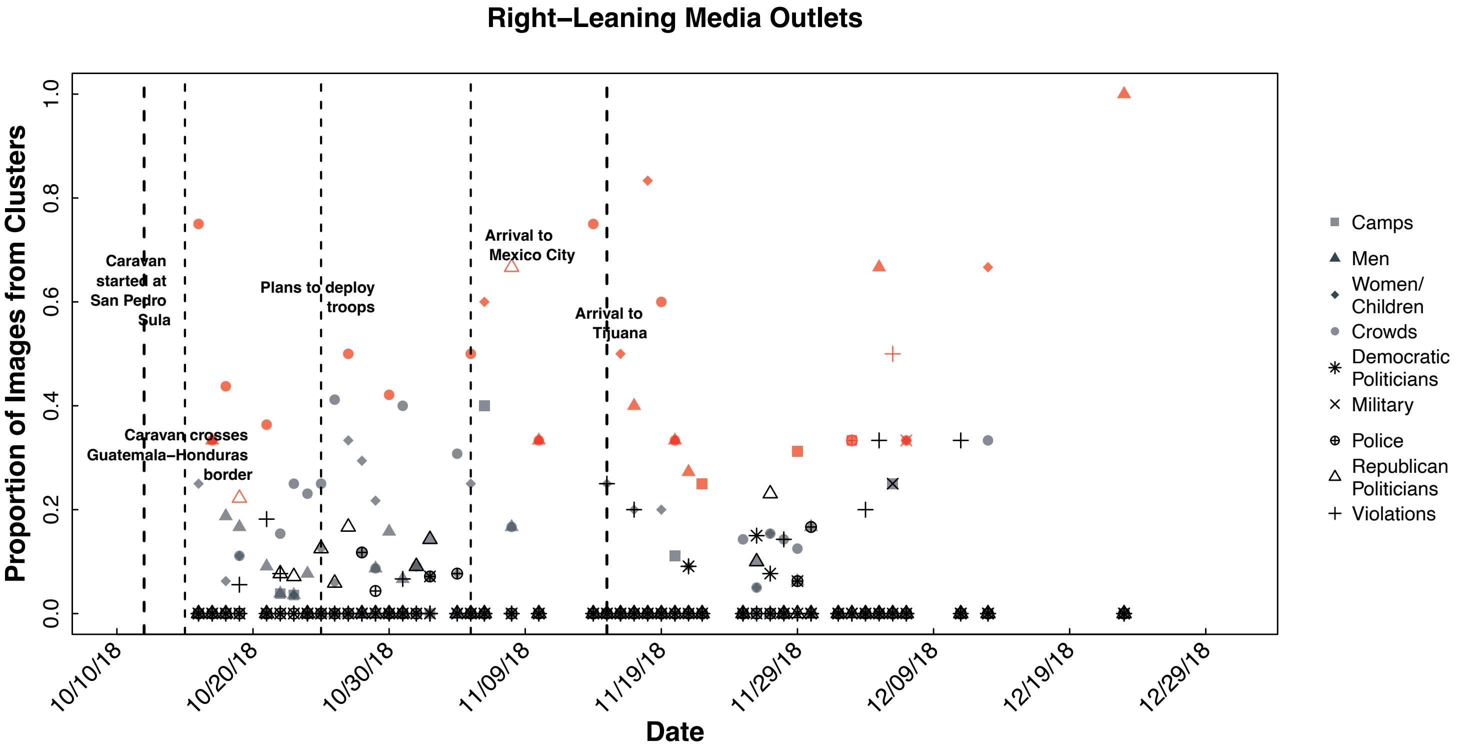

Note: The Y-axis shows the daily proportion of images from each cluster, relative to all migrant-caravan images published that day. Each point represents the overall proportion across left-leaning outlets (as defined by AllSides' "Right" (Very Conservative) and "Lean Right" (Moderate Conservative) categories). Black dashed lines mark key events; bold dashed lines indicate the caravan's departure and its arrival at the U.S.–Mexico border. Red points highlight the cluster with the highest daily proportion; days without a red point indicate that "Other" was the most prevalent cluster.
