# Supplementary material for: Media choice and audience perceptions: Evidence from visual framing of immigration in news stories
Source: PLoS One. 2025 Sep 15;20(9):e0331219. doi: 10.1371/journal.pone.0331219 (PMC12435698; doi:10.1371/journal.pone.0331219)
Supplement: S1 Appendix — (ZIP) [file pone.0331219.s001.zip › si_files/S11_Appendix.pdf]

Table S.16: Linear regressions for partisan subsamples on all clusters with "Violations" as a baseline category.

	Dependent variable:			
	Accuracy		Attitudes	
	Democrats	Republicans	Democrats	Republicans
	(1)	(2)	(3)	(4)
Camps	0.360**	−0.165	0.218	0.747***
	(0.146)	(0.183)	(0.252)	(0.267)
Close Shots (Men)	0.177*	−0.240*	0.829***	0.900***
	(0.099)	(0.125)	(0.169)	(0.178)
Close Shots (Women/Children)	0.331***	−0.175	0.902***	1.105***
	(0.086)	(0.109)	(0.148)	(0.157)
Crowds	0.184**	−0.121	0.751***	0.608***
	(0.090)	(0.113)	(0.154)	(0.162)
Democratic Politicians	0.420**	−0.401	1.378***	0.401
	(0.198)	(0.273)	(0.342)	(0.380)
Military	0.186	−0.036	0.385*	1.534***
	(0.116)	(0.145)	(0.199)	(0.209)
Police	0.143	−0.041	−0.318	1.031***
	(0.149)	(0.190)	(0.260)	(0.271)
Republican Politicians	0.224**	0.527***	−0.404**	2.286***
	(0.102)	(0.128)	(0.176)	(0.186)
Violations	[baseline category]			
Age Category	0.153***	0.141***	−0.130***	−0.111***
	(0.024)	(0.028)	(0.025)	(0.027)
Gender	−0.214**	0.090	−0.204**	0.140
	(0.087)	(0.101)	(0.093)	(0.096)
Education	−0.011	0.004	0.085**	0.062*
	(0.031)	(0.037)	(0.034)	(0.035)
Hispanic	0.014	−0.080	−0.054	−0.050
	(0.135)	(0.217)	(0.148)	(0.211)
Income	0.019	−0.013	0.048***	0.004
	(0.017)	(0.020)	(0.018)	(0.019)
Interest in Politics	0.074**	0.101**	0.053	0.037
	(0.037)	(0.044)	(0.040)	(0.042)
Constant	3.852***	3.806***	3.506***	2.990***
	(0.221)	(0.246)	(0.256)	(0.257)
Controls	✓	✓	✓	✓
Observations	4,737	3,471	5,179	3,914

Note: *p<0.1; **p<0.05; ***p<0.01. All regressions are linear models with image-level and respondent-level random effects. Standard errors are in parentheses.

Table S.17: Linear regressions for partisan subsamples on all clusters with "Close Shots (Women/Children)" as a baseline category.

	Dependent variable:			
	Accuracy		Attitudes	
	Democrats	Republicans	Democrats	Republicans
	(1)	(2)	(3)	(4)
Camps	0.030	0.009	−0.684***	−0.358
	(0.127)	(0.160)	(0.222)	(0.235)
Close Shots (Men)	−0.154**	−0.065	−0.073	−0.206
	(0.071)	(0.089)	(0.120)	(0.125)
Close Shots (Women/Children)	[baseline category]			
Crowds	−0.147***	0.054	−0.151	−0.497***
	(0.057)	(0.070)	(0.097)	(0.100)
Democratic Politicians	0.089	−0.226	0.476	−0.705**
	(0.185)	(0.258)	(0.320)	(0.358)
Military	−0.145	0.138	−0.517***	0.428***
	(0.092)	(0.115)	(0.159)	(0.165)
Police	−0.188	0.134	−1.220***	−0.074
	(0.132)	(0.169)	(0.231)	(0.240)
Republican Politicians	−0.107	0.701***	−1.306***	1.181***
	(0.074)	(0.093)	(0.128)	(0.135)
Violations	−0.331***	0.175	−0.902***	−1.105***
	(0.086)	(0.109)	(0.148)	(0.157)
Age Category	0.153***	0.141***	−0.130***	−0.111***
	(0.024)	(0.028)	(0.025)	(0.027)
Gender	−0.214**	0.090	−0.204**	0.140
	(0.087)	(0.101)	(0.093)	(0.096)
Education	−0.011	0.004	0.085**	0.062*
	(0.031)	(0.037)	(0.034)	(0.035)
Hispanic	0.014	−0.080	−0.054	−0.050
	(0.135)	(0.217)	(0.148)	(0.211)
Income	0.019	−0.013	0.048***	0.004
	(0.017)	(0.020)	(0.018)	(0.019)
Interest in Politics	0.074**	0.101**	0.053	0.037
	(0.037)	(0.044)	(0.040)	(0.042)
Constant	4.183***	3.631***	4.408***	4.095***
	(0.209)	(0.228)	(0.227)	(0.222)
Controls	✓	✓	✓	✓
Observations	4,737	3,471	5,179	3,914

Note: *p<0.1; **p<0.05; ***p<0.01. All regressions are linear models with image-level and respondent-level random effects. Standard errors are in parentheses.

Table S.18: Linear regressions for partisan subsamples on all clusters with "Crowds" as a baseline category.

	Dependent variable:			
	Accuracy		Attitudes	
	Democrats	Republicans	Democrats	Republicans
	(1)	(2)	(3)	(4)
Camps	0.176	−0.045	−0.533**	0.138
	(0.130)	(0.163)	(0.226)	(0.239)
Close Shots (Men)	−0.007	−0.119	0.079	0.291**
	(0.075)	(0.093)	(0.127)	(0.132)
Close Shots (Women/Children)	0.147***	−0.054	0.151	0.497***
	(0.057)	(0.070)	(0.097)	(0.100)
Crowds		[baseline category]		
Democratic Politicians	0.235	−0.280	0.627*	−0.208
	(0.186)	(0.260)	(0.322)	(0.360)
Military	0.002	0.084	−0.366**	0.925***
	(0.096)	(0.118)	(0.164)	(0.170)
Police	−0.042	0.080	−1.068***	0.422*
	(0.135)	(0.171)	(0.234)	(0.243)
Republican Politicians	0.040	0.647***	−1.155***	1.678***
	(0.079)	(0.097)	(0.135)	(0.141)
Violations	−0.184**	0.121	−0.751***	−0.608***
	(0.090)	(0.113)	(0.154)	(0.162)
Age Category	0.153***	0.141***	−0.130***	−0.111***
	(0.024)	(0.028)	(0.025)	(0.027)
Gender	−0.214**	0.090	−0.204**	0.140
	(0.087)	(0.101)	(0.093)	(0.096)
Education	−0.011	0.004	0.085**	0.062*
	(0.031)	(0.037)	(0.034)	(0.035)
Hispanic	0.014	−0.080	−0.054	−0.050
	(0.135)	(0.217)	(0.148)	(0.211)
Income	0.019	−0.013	0.048***	0.004
	(0.017)	(0.020)	(0.018)	(0.019)
Interest in Politics	0.074**	0.101**	0.053	0.037
	(0.037)	(0.044)	(0.040)	(0.042)
Constant	4.036***	3.685***	4.257***	3.598***
	(0.211)	(0.230)	(0.230)	(0.226)
Controls	✓	✓	✓	✓
Observations	4,737	3,471	5,179	3,914

Note: *p<0.1; **p<0.05; ***p<0.01. All regressions are linear models with image-level and respondent-level random effects. Standard errors are in parentheses.
