# Supplementary material for: Media choice and audience perceptions: Evidence from visual framing of immigration in news stories
Source: PLoS One. 2025 Sep 15;20(9):e0331219. doi: 10.1371/journal.pone.0331219 (PMC12435698; doi:10.1371/journal.pone.0331219)
Supplement: S1 Appendix — (ZIP) [file pone.0331219.s001.zip › si_files/S20_Table.pdf]

Table S.20: Linear regressions for partisan subsamples on all clusters with "Women/Children" as a baseline category.

	Dependent variable:	
	Is it a Liberal Outlet?	
	Democrats	Republicans
	(1)	(2)
Camps	0.028	−0.007
	(0.049)	(0.057)
Close Shots (Men)	−0.037	0.003
	(0.027)	(0.029)
Close Shots (Women/Children)	[baseline category]	
Crowds	−0.114***	−0.055**
	(0.021)	(0.024)
Democratic Politicians	−0.053	0.069
	(0.068)	(0.081)
Military	−0.213***	−0.251***
	(0.035)	(0.037)
Police	−0.191***	−0.076
	(0.049)	(0.058)
Republican Politicians	−0.208***	−0.366***
	(0.027)	(0.030)
Violations	−0.152***	−0.123***
	(0.032)	(0.037)
Age Category	0.016*	0.044***
	(0.008)	(0.009)
Gender	0.017	−0.015
	(0.029)	(0.034)
Education	−0.007	0.011
	(0.011)	(0.012)
Hispanic	0.037	0.058
	(0.045)	(0.071)
Income	−0.006	−0.010
	(0.006)	(0.007)
Interest in Politics	0.009	−0.017
	(0.013)	(0.015)
Constant	0.401***	0.428***
	(0.071)	(0.078)
Controls	✓	✓
Observations	3,153	2,247

Note: *p<0.1; **p<0.05; ***p<0.01. All regressions are linear models with image-level and respondent-level random effects. Standard errors are in parentheses.
