# Supplementary material for: Media choice and audience perceptions: Evidence from visual framing of immigration in news stories
Source: PLoS One. 2025 Sep 15;20(9):e0331219. doi: 10.1371/journal.pone.0331219 (PMC12435698; doi:10.1371/journal.pone.0331219)
Supplement: S1 Appendix — (ZIP) [file pone.0331219.s001.zip › si_files/S18_Table.pdf]

Table S.18: Linear regressions for partisan subsamples on all clusters with "Crowds" as a baseline category.

	Dependent variable:			
	Accuracy		Attitudes	
	Democrats	Republicans	Democrats	Republicans
	(1)	(2)	(3)	(4)
Camps	0.176	−0.045	−0.533**	0.138
	(0.130)	(0.163)	(0.226)	(0.239)
Close Shots (Men)	−0.007	−0.119	0.079	0.291**
	(0.075)	(0.093)	(0.127)	(0.132)
Close Shots (Women/Children)	0.147***	−0.054	0.151	0.497***
	(0.057)	(0.070)	(0.097)	(0.100)
Crowds	[baseline category]			
Democratic Politicians	0.235	−0.280	0.627*	−0.208
	(0.186)	(0.260)	(0.322)	(0.360)
Military	0.002	0.084	−0.366**	0.925***
	(0.096)	(0.118)	(0.164)	(0.170)
Police	−0.042	0.080	−1.068***	0.422*
	(0.135)	(0.171)	(0.234)	(0.243)
Republican Politicians	0.040	0.647***	−1.155***	1.678***
	(0.079)	(0.097)	(0.135)	(0.141)
Violations	−0.184**	0.121	−0.751***	−0.608***
	(0.090)	(0.113)	(0.154)	(0.162)
Age Category	0.153***	0.141***	−0.130***	−0.111***
	(0.024)	(0.028)	(0.025)	(0.027)
Gender	−0.214**	0.090	−0.204**	0.140
	(0.087)	(0.101)	(0.093)	(0.096)
Education	−0.011	0.004	0.085**	0.062*
	(0.031)	(0.037)	(0.034)	(0.035)
Hispanic	0.014	−0.080	−0.054	−0.050
	(0.135)	(0.217)	(0.148)	(0.211)
Income	0.019	−0.013	0.048***	0.004
	(0.017)	(0.020)	(0.018)	(0.019)
Interest in Politics	0.074**	0.101**	0.053	0.037
	(0.037)	(0.044)	(0.040)	(0.042)
Constant	4.036***	3.685***	4.257***	3.598***
	(0.211)	(0.230)	(0.230)	(0.226)
Controls	✓	✓	✓	✓
Observations	4,737	3,471	5,179	3,914

Note: *p<0.1; **p<0.05; ***p<0.01. All regressions are linear models with image-level and respondent-level random effects. Standard errors are in parentheses.
