# Supplementary material for: Media choice and audience perceptions: Evidence from visual framing of immigration in news stories
Source: PLoS One. 2025 Sep 15;20(9):e0331219. doi: 10.1371/journal.pone.0331219 (PMC12435698; doi:10.1371/journal.pone.0331219)
Supplement: S1 Appendix — (ZIP) [file pone.0331219.s001.zip › si_files/S5_Fig.pdf]

Fig. S.4: Examples of images from K-means clusters.

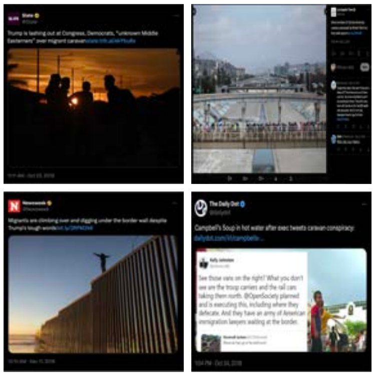

(a) Cluster 1

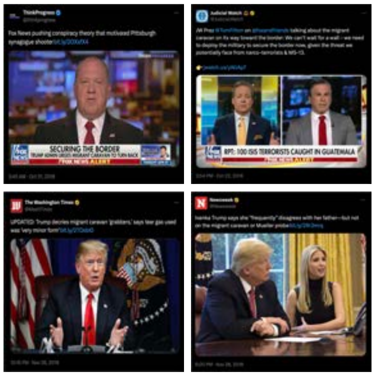

(b) Cluster 2

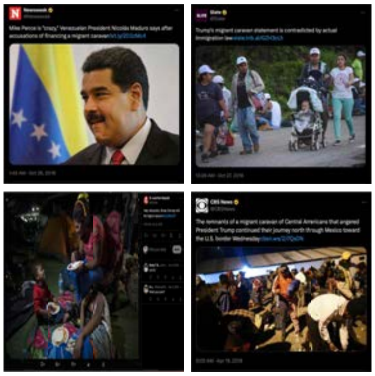

(c) Cluster 3

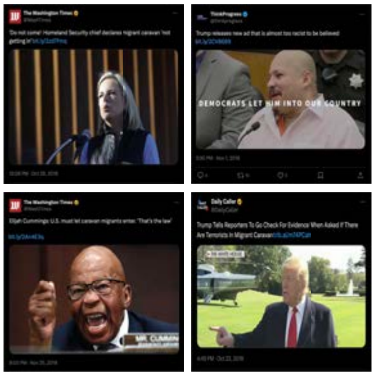

(d) Cluster 5

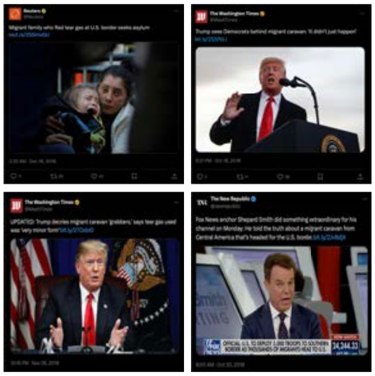

(e) Cluster 6

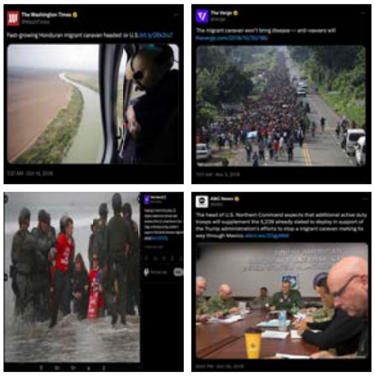

(f) Cluster 7

Fig. S.5: Examples of unsupervised clustering errors

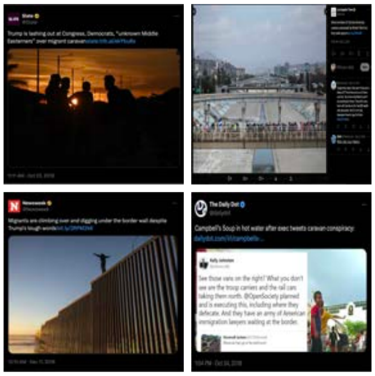

(a) Cluster 1

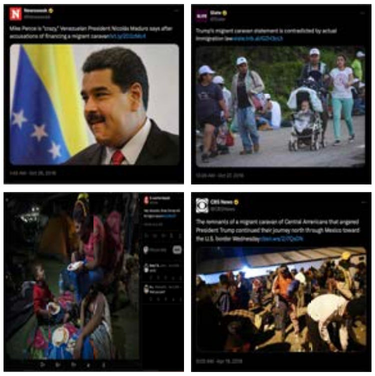

(b) Cluster 3

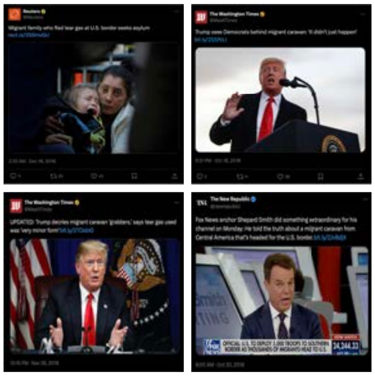

(c) Cluster 6
