# Supplementary material for: Media choice and audience perceptions: Evidence from visual framing of immigration in news stories
Source: PLoS One. 2025 Sep 15;20(9):e0331219. doi: 10.1371/journal.pone.0331219 (PMC12435698; doi:10.1371/journal.pone.0331219)
Supplement: S1 Appendix — (ZIP) [file pone.0331219.s001.zip › si_files/S7_Table.pdf]

4-Moderate							
7-Conservative							

Note: Descriptive statistics are provided for all respondents. Age is a categorical variable structured based on age ranges.

Table S.7: Descriptive statistics for survey respondents (excluding participants with self-reported partisanship as Independent).

	N	Mean	St. Dev.	Min	Pct(25)	Pct(75)	Max
**N of participants**	1425						
**Age (by age group)**	1425	4.85	1.81	2.00	3.00	6.00	9.00
1- Under 18							
2- 18 - 24							
3- 25 - 34							
4- 35 - 44							
5- 45 - 54							
6- 55 - 64							
7- 65 - 74							
8- 75 - 84							
9- 85 or older							
**Gender**	1425	0.59	0.49	0.00	0.00	1.00	1.00
0-Male							
1-Female							
**Education**	1410	3.83	1.56	1.00	2.00	5.00	7.00
1-Less than high school							
2-High school graduate							
3-Some college but no degree							
4-Associate's degree in college (2-year)							
5-Bachelor's degree in college (4-year)							
6-Master's degree							
7-Doctoral degree							
**Hispanic Ethnicity**	1417	0.08	0.27	0.00	0.00	0.00	1.00
0-Not Hispanic							
1-Hispanic							
**Income**	1421	5.62	2.97	1.00	3.00	8.00	10.00
1-Less than $10,000							
2-$10,000 - $19,999							
3-$20,000 - $29,999							
4-$30,000 - $39,999							
5-$40,000 - $49,999							
6-$50,000 - $59,999							
7-$60,000 - $69,999							
8-$70,000 - $89,999							
9-$90,000 - $109,999							
10-More than $110,000							
**Interest**	1364	3.90	1.18	1.00	4.00	5.00	5.00
1-Not interested at all							
5-Very interested							
**Ideology**	1425	3.68	2.04	1.00	2.00	5.00	7.00
1-Very Conservative							
7-Very Liberal							
**Partisanship (binary)**	1425	1.44	0.50	1.00	1.00	2.00	2.00
1-Democrat							
2-Republican							
**Respondent's Perception of Accuracy**	1120	4.58	1.64	1.00	4.00	6.00	7.00
1-Faulty							

4-Middle Ground							
7-Accurate							
**Respondent's Attitudes**	1320	4.04	1.88	1.00	3.00	5.00	7.00
1-Extremely Negative							
4-Middle Ground							
7-Extremely Positive							
**Respondent's Guess of Outlet Ideology**	1081	4.02	1.92	1.00	3.00	5.00	7.00
1-Liberal							
4-Moderate							
7-Conservative							

Note: Descriptive statistics are provided for all respondents. Age is a categorical variable structured based on age ranges.

S6.1 Sample demographics vs. Population demographics

Tables S.8, S.9, and S.10 present the distribution of sample respondents by demographic characteristics (gender, age, and ethnicity) in comparison to the estimates of general population. The general population distributions are derived from the Census Bureau's American Community Survey (ACS) Public Use Microdata Sample (PUMS) based on person files, built based on a five-year period (2019-2013) and reported in 2023 (https://www2.census.gov/programs-surveys/acs/data/pums/). We can observe similar distribution patterns across demographic groups. The observed differences are in gender and several age groups. Our sample has a larger proportion of females and slightly over representing population of age groups "25-34" and "35-44."

Table S.8: Distribution by gender.

	Census Freq	Survey Freq
Male	0.49	0.41
Female	0.51	0.59
