# Supplementary material for: Media choice and audience perceptions: Evidence from visual framing of immigration in news stories
Source: PLoS One. 2025 Sep 15;20(9):e0331219. doi: 10.1371/journal.pone.0331219 (PMC12435698; doi:10.1371/journal.pone.0331219)
Supplement: S1 Appendix — (ZIP) [file pone.0331219.s001.zip › si_files/S15_Fig.pdf]

Fig. S.15: Media outlet ideology guesses for low-confidence respondents.

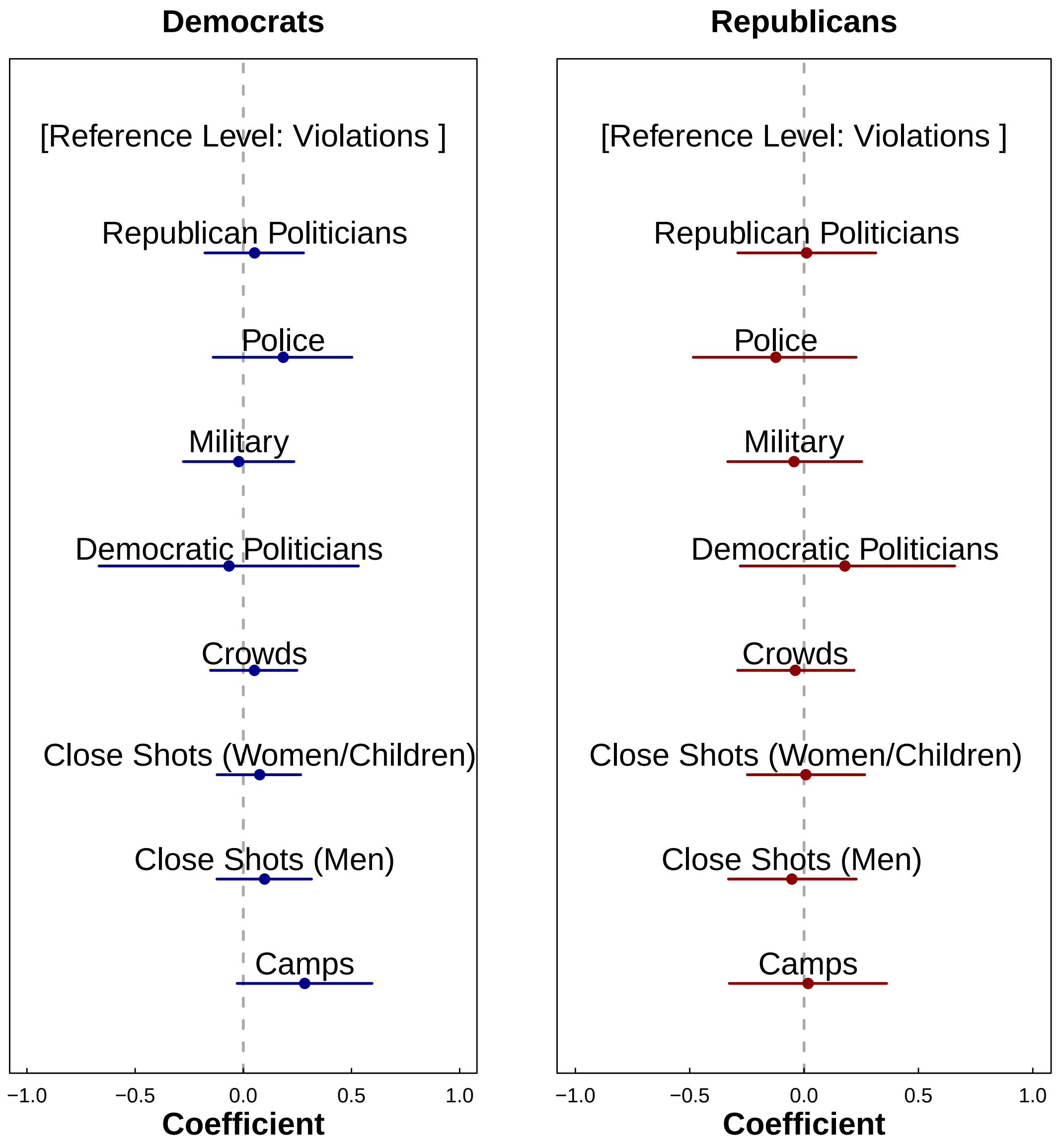

Note: The plot presents results from two linear models with random effects (random intercepts for images and respondents): one for Democratic respondents and one for Republican respondents. Each model includes only respondents reporting low confidence (ratings less than 4 on the 7-point scale). Each line shows an estimated regression coefficient (with 95% CI) for visual-frame predictors, using the "Violations" cluster as the reference. The outcome is a binary indicator (1 = liberal outlet guess; 0 = conservative outlet guess). Both models control for gender, age, ethnicity, income, education, and interest in politics.
