# Supplementary material for: Media choice and audience perceptions: Evidence from visual framing of immigration in news stories
Source: PLoS One. 2025 Sep 15;20(9):e0331219. doi: 10.1371/journal.pone.0331219 (PMC12435698; doi:10.1371/journal.pone.0331219)
Supplement: S1 Appendix — (ZIP) [file pone.0331219.s001.zip › si_files/S26_Table.pdf]

Table S.24: Scoring correlation table: Democrats.

	Outlet: Prop Liberal	Outlet: Prop Conservative
Respondent Guess: Prop Liberal	0.302	-0.001
Respondent Guess: Prop Conservative	-0.302	0.001

Table S.25: Scoring correlation table: Republicans.

	Outlet: Prop Liberal	Outlet: Prop Conservative
Respondent Guess: Prop Liberal	0.261	0.164
Respondent Guess: Prop Conservative	-0.261	-0.164

S16 Curated Labeling

Table S.26 presents the distribution of curated labels across all 2,006 harvested images and Table S.27 shows distribution of curated labels for images used in the survey wave. The ”Other” category is a residual group that includes images containing text, multiple aligned images of political figures, and images with logos (e.g., news channel logos). The ”Undefined” category includes images for which coders did not reach agreement.

Table S.26: Curated labels cluster sizes.

Curated Label	Number of Images
Camps	68
Close Shots (Men)	186
Close Shots (Women/Children)	349
Crowds	338
Democratic Politicians	7
Military	59
Police	33
Republican Politicians	170
Violations	93
Other	602
Undefined	101

In Figure S.16 we demonstrate examples of curated labels, and the way how such a curated labeling potentially can improve classification task.
