# Supplementary material for: Media choice and audience perceptions: Evidence from visual framing of immigration in news stories
Source: PLoS One. 2025 Sep 15;20(9):e0331219. doi: 10.1371/journal.pone.0331219 (PMC12435698; doi:10.1371/journal.pone.0331219)
Supplement: S1 Appendix — (ZIP) [file pone.0331219.s001.zip › si_files/S1_Table.pdf]

Table S.1: List of media outlets.

Category	Items
**Very Liberal (Left)**	AJ+, AlterNet, Aquinas College Saint, Arkansas Democrat-Gazette, Block Club Chicago, Blue Virginia, Boing Boing, Brown Girl Magazine, BuzzFeed News, Care2, Chicago Crusader, Chicago Defender, CNN (Online News), CNN (Opinion), Common Dreams, Current Affairs, Daily Beast, Daily Chela, Daily Kos, Democracy Now, Esquire, Falls Church News - Press, Herald Democrat, Hip Latina, HuffPost, Jacobin, Jezebel, Latino Rebels, Leafly, LGBTQ Nation, Mashable, Media Matters, Metro Weekly, Mother Jones, MSNBC, New Republic, New York Daily News, New York Magazine, New York Times (Opinion), NewsOne, Peacock Panache, PinkNews, PoliticusUSA, Raw Story, Refinery29, RollingStone.com, Salon, San Francisco Chronicle, Slate, Socialist Alternative, Splinter, The Boston Globe, The Intercept, The Juggernaut, The Nation, The New Yorker, ThinkProgress, Truthdig, Upworthy, Vice, Vox, Yes! Magazine
**Moderate Liberal (Lean Left)**	ABC News (Online), Above The Law, Al Jazeera, Atlanta Black Star, Atlanta Journal-Constitution, Austin American-Statesman, BET, Black Enterprise, Bloomberg, Boston Review, Bowling Green Daily News, Bustle, CBS News (Online), Center for Public Integrity, Chicago Sun-Times, CNN Business, Counter Currents, Daily Northwestern, Daily Targum, Detroit Free Press, East Bay Times, Elle Magazine, ESPN.com, FAIR, Gizmodo, GLAAD, Google News, GQ.com, Grist, Harvard Business School, High Times, Indy Week, Las Vegas Sun, Longmont Times-Call, Los Angeles Times, Louisville Courier-Journal, Marijuana Moment, Mediaite, Miami Herald, Michigan Daily, MTV News Online, NBC News (Online), NBC Today Show, New York Times (News), Pacific Standard, Politico, ProPublica, San Diego Union-Tribune, San Jose Mercury News, Scientific American, SFGate, Sky-Hi Daily News, Spokesman Review, Star Tribune, State Journal, Teen Vogue, The Advocate, The Advocate-Messenger, The Atlantic, The Cadiz Record, The Colorado Sun, The Commercial Appeal, The Daily Dot, The Delaware County Daily Times, The Economist, The Guardian, The Hollywood Reporter, The Justice, The Maneater, The Onion (Humor), The Philadelphia Inquirer, The Root, The Sacramento Bee, The Texas Observer, The Texas Tribune, The Verge, The Week - News, Time Magazine, TruthOut, U.S. News & World Report, Univision, USA TODAY, Vanity Fair, VT Digger, Washington Monthly, Washington Post, Wisconsin Gazette, Yahoo News

**Moderate (Center)**	Arc Digital, Associated Press, Axios, AZ Central, Barnstable Patriot, BBC News, Bridgemi.com, C-SPAN, CalMatters, CalWatchdog, Chicago Tribune, Christian Science Monitor, CNBC, CNET, Columbia Journalism Review, Columbia Missourian, Concord Monitor, Cook Report, CrowdPAC, CU Independent, Daily Breeze, Daily Cardinal, Daily Progress, Defense One, Des Moines Register, Detroit News, Deutsche Welle, Diplomatic Courier, Duke Chronicle, Education Week, Erraticus, Estes Park Trail Gazette, Eurek Alert, Financial Times, FiveThirtyEight, Forbes, Foreign Affairs, Foreign Policy, Fort Worth Star-Telegram, Fortune, GoLocal Providence, Haaretz, Hampton Roads Messanger, Heavy.com, Honolulu Civil Beat, Houston Chronicle, Idaho Statesman, Independent Journal Review, Indiana Daily Student, Indy Online, IndyStar, Inland Valley Daily Bulletin, Inside Philanthropy, International Business Times, IVN, Jefferson Public Radio, Journalist's Resource, JSTOR Daily, Jubilee Media, Just Security, Just The News, KALW.org, KATU, Kenosha News, KQED, KWCH, Law & Crime, Lifehacker, Live Science, Long Beach Press-Telegram, Los Angeles Daily News, Making Sense, MarketWatch, McClatchyDC, Media Village, Military Times, Misinformation Review, MIT News, National Geographic, National Journal, Nature.com, Nautilus Quarterly, New Discourses, New Hampshire Union Leader, Newsweek, Newsy, Newtrals, Nieman Lab, NMPolitics.net, NPR (Online News), Our.News, Outkick the Coverage, Palm Springs Desert Sun, Pasadena Star-News, Patch.com, PBS NewsHour, Phys.org, Portland Press Herald, Poynter, PRI (Public Radio International), Psychology Today, Quartz, Rasmussen Reports, RealClearPolitics, Record Journal, Redlands Daily Facts, Reuters, Roll Call, San Antonio Express-News, San Bernardino Sun, San Gabriel Valley Tribune, Science Daily, SCOTUSblog, Scriberr Media - News, SF Weekly, Smerconish, Smithsonian Magazine, South China Morning Post, St. Louis Post-Dispatch, STAT, Tallahassee Democrat, Tampa Bay Times, Tech Xplore, TechCrunch, The Appeal, The Bellows, The Columbus Dispatch, The Dallas Morning News, The Fulcrum, The Globe and Mail, The Hill, The Japan Times, The Jerusalem Post, The Korea Herald, The Lufkin Daily News, The Markup, The Marshall Project, The Observer (New York), The Oracle, The Oregonian, The Red and Black, The Saturday Evening Post, The Seattle Times, The South African, The Tennesseean, The Times-Picayune, Times Union, Variety, Virginia Mercury, Voice of America, Volante, Wake Up to Politics, Wall Street Journal (News), WANDTV, WFAE, WGBH, WGN, Whittier Daily News, Wired
**Moderate Conservative (Lean Right)**	Babylon Bee (Humor), Boston Herald, Christianity Today, Daily Press, Deseret News, Desiring God, Drudge Report, Evie Magazine, Fiscal Times, Fox Business, HotAir, Investor's Business Daily, Judicial Watch, Leesburg Today, Live Action News, Meridian Magazine, New York Post (News), Newsmax (News), Orange County Register, Pittsburgh Post-Gazette, Project Veritas, Quillette, Reason, Richmond Times Dispatch, Spiked, Tablet Mag, The American Conservative, The American Mind, The Bulwark, The Christian Post, The Dispatch, The Epoch Times, The Libertarian Republic, The Police Tribune, The Post Millennial, The Press-Enterprise, The Telegraph - UK, The Texan, Wall Street Journal (Opinion), Washington Examiner, Washington Times, Watchdog.org, ZeroHedge
**Very Conservative (Right)**	American Greatness, American Thinker, Bearing Drift, Biblical Gender Roles, Bizpac Review, Breitbart News, Brownstone Institute, CBN, City Journal, CNSNews.com, Commentary Magazine, Daily Citizen, Daily Mail, Fox News (Online News), Fox News Latino, FrontPage Magazine, Inacow, KSL, Media Research Center, National Review, New York Post (Opinion), NewsBusters, One America News Network (OAN), PJ Media, RedState, Right Side News, The American Spectator, The College Fix, The Daily Caller, The Daily Signal, The Daily Wire, The Federalist, The Gateway Pundit, The Imaginative Conservative, The Resurgent, The Weekly Standard, The Western Journal, TheBlaze.com, Townhall, Washington Free Beacon, WND.com
