# Supplementary material for: Media choice and audience perceptions: Evidence from visual framing of immigration in news stories
Source: PLoS One. 2025 Sep 15;20(9):e0331219. doi: 10.1371/journal.pone.0331219 (PMC12435698; doi:10.1371/journal.pone.0331219)
Supplement: S1 Appendix — (ZIP) [file pone.0331219.s001.zip › si_files/S9_Table.pdf]

Table S.9: Distribution by age.

	Census Freq	Survey Freq
18 - 24	0.106	0.107
25 - 34	0.147	0.166
35 - 44	0.148	0.199
45 - 54	0.149	0.129
55 - 64	0.178	0.174
65 - 74	0.159	0.157
75 - 84	0.083	0.060
85 and older	0.031	0.008

Table S.10: Distribution by Hispanic ethnicity.

	Census Freq	Survey Freq
Not Hispanic	0.86	0.92
Hispanic	0.14	0.08

S7 Manipulation Check

Table S.11: Results of multinomial logistic regression for the randomization check.

	Dependent variable:							
	Camps	Close Shots (Men)	Crowds	Democratic Politicians	Military	Police	Republican Politicians	Violations
	(1)	(2)	(3)	(4)	(5)	(6)	(7)	(8)
Party (Binary)	−0.044	−0.049	−0.092	0.239	−0.058	−0.015	0.038	0.003
	(0.128)	(0.070)	(0.056)	(0.197)	(0.092)	(0.133)	(0.075)	(0.088)
Age	−0.002	0.005	−0.009	−0.057	0.022	0.003	0.017	−0.016
	(0.036)	(0.020)	(0.016)	(0.054)	(0.026)	(0.037)	(0.021)	(0.025)
Gender	0.107	0.002	0.052	−0.064	0.084	−0.065	−0.004	−0.033
	(0.130)	(0.072)	(0.058)	(0.198)	(0.094)	(0.136)	(0.077)	(0.090)
Education	0.017	−0.045*	−0.018	0.051	0.001	0.055	−0.031	0.001
	(0.047)	(0.026)	(0.021)	(0.071)	(0.034)	(0.049)	(0.028)	(0.032)
Hispanic	0.539*	0.044	0.091	0.226	−0.090	−0.013	−0.058	0.109
	(0.286)	(0.130)	(0.104)	(0.360)	(0.164)	(0.238)	(0.134)	(0.163)
Income	0.014	0.030**	0.004	−0.022	0.0001	−0.034	−0.015	−0.002
	(0.025)	(0.014)	(0.011)	(0.037)	(0.018)	(0.026)	(0.015)	(0.017)
Interest in Politics	−0.022	−0.037	0.003	0.072	−0.023	0.073	−0.004	0.021
	(0.056)	(0.030)	(0.025)	(0.088)	(0.040)	(0.061)	(0.033)	(0.039)
Constant	−3.024***	−0.936***	−0.343**	−3.722***	−1.657***	−2.812***	−1.053***	−1.679***
	(0.376)	(0.185)	(0.149)	(0.527)	(0.240)	(0.356)	(0.196)	(0.235)
AIC	35,262.800	35,262.800	35,262.800	35,262.800	35,262.800	35,262.800	35,262.800	35,262.800

Note: *p<0.1; **p<0.05; ***p<0.01. Each column presents the results for a given image cluster in comparison with the reference category cluster category - ”Close Shots (Women/Children)”.
