# Supplementary material for: Media choice and audience perceptions: Evidence from visual framing of immigration in news stories
Source: PLoS One. 2025 Sep 15;20(9):e0331219. doi: 10.1371/journal.pone.0331219 (PMC12435698; doi:10.1371/journal.pone.0331219)
Supplement: S1 Appendix — (ZIP) [file pone.0331219.s001.zip › si_files/S23_Table.pdf]

S14 Outlets Ideology and the Choice of Visual Frames

Table S.23: Visual frames that liberal media outlets use more often than conservative media outlets: Binary OLS Results.

	Dependent variable:								
	Camps	Men	Women/Children	Crowds	Dem Politicians	Military	Police	Rep Politicians	Violations
	(1)	(2)	(3)	(4)	(5)	(6)	(7)	(8)	(9)
Outlet Ideology (R-L)	−0.002	−0.003	0.057***	−0.034***	−0.004**	0.010**	−0.001	0.011	−0.010*
	(0.005)	(0.008)	(0.010)	(0.010)	(0.002)	(0.004)	(0.003)	(0.007)	(0.006)
Constant	0.039***	0.100***	0.055**	0.240***	0.012***	0.009	0.018**	0.062***	0.067***
	(0.011)	(0.017)	(0.022)	(0.022)	(0.004)	(0.010)	(0.008)	(0.017)	(0.013)
Observations	2,006	2,006	2,006	2,006	2,006	2,006	2,006	2,006	2,006

Note: In all models, each image constitutes an observation. Each model is a separate OLS regression predicting a binary outcome (1 = image belongs to the specified visual frame; 0 = it does not), with no additional control variables or fixed effects. The sole predictor is media outlet ideology, measured on a 3-point ordinal scale (1 = conservative outlet; 2 = moderate outlet; 3 = liberal outlet). These results illustrate the simple association between outlet ideology and the likelihood of an image appearing in each visual frame.
