# Supplementary material for: Media choice and audience perceptions: Evidence from visual framing of immigration in news stories
Source: PLoS One. 2025 Sep 15;20(9):e0331219. doi: 10.1371/journal.pone.0331219 (PMC12435698; doi:10.1371/journal.pone.0331219)
Supplement: S1 Appendix — (ZIP) [file pone.0331219.s001.zip › si_files/S8_Fig.pdf]

Fig. S.8: Visual frames and ideology of media outlets: Alternative ideology measure.

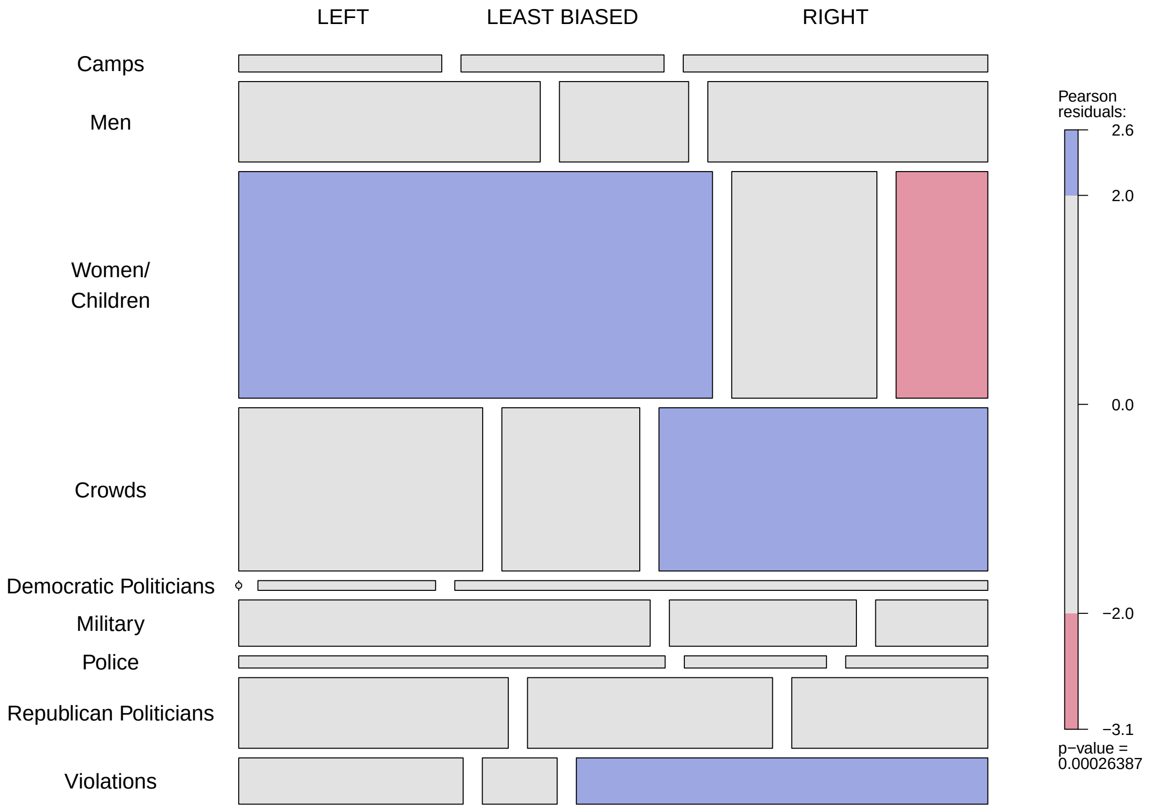

Note: This plot shows the relationship between two nominal variables of interest: (1) media outlet ideology (ranging from very left-leaning to very right-leaning) and (2) image cluster. Colors indicate both the direction and strength of associations between categories, as measured by Pearson standardized residuals—the deviation of observed counts from those expected under independence. Blue shading denotes positive associations (more cases than expected), red shading denotes negative associations (fewer cases than expected), and gray indicates no meaningful association. The p-value displayed corresponds to a Chi-square test of independence and rejects the null hypothesis of no association between the two variables.
