# Supplementary material for: Media choice and audience perceptions: Evidence from visual framing of immigration in news stories
Source: PLoS One. 2025 Sep 15;20(9):e0331219. doi: 10.1371/journal.pone.0331219 (PMC12435698; doi:10.1371/journal.pone.0331219)
Supplement: S1 Appendix — (ZIP) [file pone.0331219.s001.zip › si_files/S16_Table.pdf]

Table S.16: Linear regressions for partisan subsamples on all clusters with "Violations" as a baseline category.

	Dependent variable:			
	Accuracy		Attitudes	
	Democrats	Republicans	Democrats	Republicans
	(1)	(2)	(3)	(4)
Camps	0.360**	−0.165	0.218	0.747***
	(0.146)	(0.183)	(0.252)	(0.267)
Close Shots (Men)	0.177*	−0.240*	0.829***	0.900***
	(0.099)	(0.125)	(0.169)	(0.178)
Close Shots (Women/Children)	0.331***	−0.175	0.902***	1.105***
	(0.086)	(0.109)	(0.148)	(0.157)
Crowds	0.184**	−0.121	0.751***	0.608***
	(0.090)	(0.113)	(0.154)	(0.162)
Democratic Politicians	0.420**	−0.401	1.378***	0.401
	(0.198)	(0.273)	(0.342)	(0.380)
Military	0.186	−0.036	0.385*	1.534***
	(0.116)	(0.145)	(0.199)	(0.209)
Police	0.143	−0.041	−0.318	1.031***
	(0.149)	(0.190)	(0.260)	(0.271)
Republican Politicians	0.224**	0.527***	−0.404**	2.286***
	(0.102)	(0.128)	(0.176)	(0.186)
Violations	[baseline category]			
Age Category	0.153***	0.141***	−0.130***	−0.111***
	(0.024)	(0.028)	(0.025)	(0.027)
Gender	−0.214**	0.090	−0.204**	0.140
	(0.087)	(0.101)	(0.093)	(0.096)
Education	−0.011	0.004	0.085**	0.062*
	(0.031)	(0.037)	(0.034)	(0.035)
Hispanic	0.014	−0.080	−0.054	−0.050
	(0.135)	(0.217)	(0.148)	(0.211)
Income	0.019	−0.013	0.048***	0.004
	(0.017)	(0.020)	(0.018)	(0.019)
Interest in Politics	0.074**	0.101**	0.053	0.037
	(0.037)	(0.044)	(0.040)	(0.042)
Constant	3.852***	3.806***	3.506***	2.990***
	(0.221)	(0.246)	(0.256)	(0.257)
Controls	✓	✓	✓	✓
Observations	4,737	3,471	5,179	3,914

Note: *p<0.1; **p<0.05; ***p<0.01. All regressions are linear models with image-level and respondent-level random effects. Standard errors are in parentheses.
