# Supplementary material for: Media choice and audience perceptions: Evidence from visual framing of immigration in news stories
Source: PLoS One. 2025 Sep 15;20(9):e0331219. doi: 10.1371/journal.pone.0331219 (PMC12435698; doi:10.1371/journal.pone.0331219)

Table S.4: Distribution of images across clusters.

	Number of Images	Proposed Cluster Topic
Cluster 1	208	Border/Night Views
Cluster 2	188	News Anchors/Public Speakers
Cluster 3	110	Crowds/Camps
Cluster 4	499	Groups of People
Cluster 5	107	Public Speakers
Cluster 6	590	Politicians/News Anchors
Cluster 7	307	Enforcement/Crowds

Fig. S.3: Optimal number of K clusters testing.

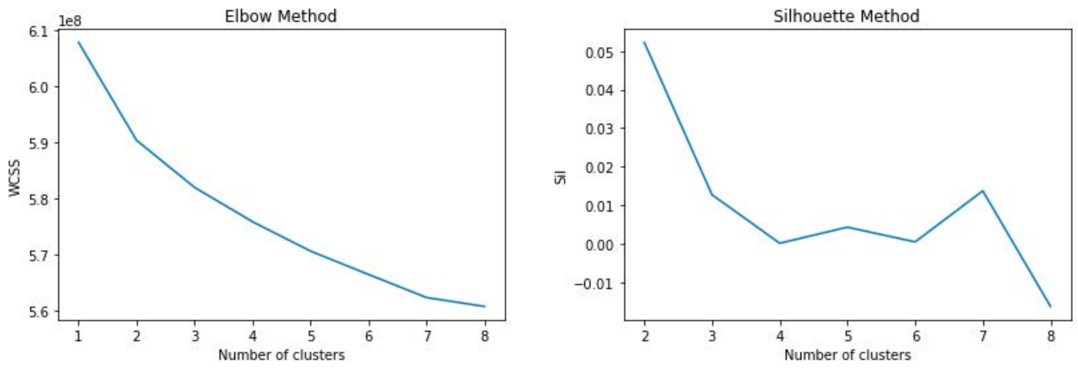

Supplement: S1 Appendix — (ZIP) [file pone.0331219.s001.zip › si_files/S4_Table.pdf]
