# Supplementary material for: Media choice and audience perceptions: Evidence from visual framing of immigration in news stories
Source: PLoS One. 2025 Sep 15;20(9):e0331219. doi: 10.1371/journal.pone.0331219 (PMC12435698; doi:10.1371/journal.pone.0331219)

S8 Power Analysis Simulations

To estimate the necessary sample size for detecting a minimal identifiable effect, we conducted a power analysis using simulations (with $N = 500$ number of simulations). Given that our outcomes of interest are measured on a 7-point scale, we set the minimal detectable effect at 0.3. In the simulation exercise, we also included a specification with covariates—specifically gender and age—whose effects can influence statistical power. Figure S.11 presents the simulation results, showing that a sample size greater than 1,000 respondents yields a power exceeding 0.8 in the model with covariates. Based on these findings, and considering that all our models are estimated separately for self-identified Democrats and Republicans, we recruited 3,000 participants with quotas balanced across partisanship (self-identified Democrats, Republicans, and Independents).

Fig. S.11: Power simulation.

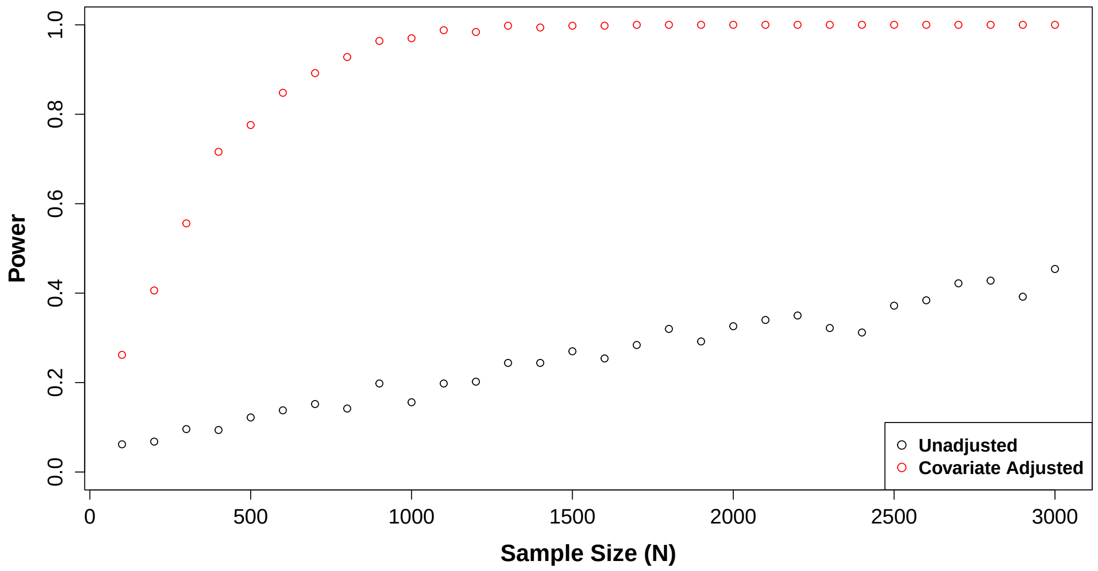

Supplement: S1 Appendix — (ZIP) [file pone.0331219.s001.zip › si_files/S8_Appendix.pdf]
