# Supplementary material for: Media choice and audience perceptions: Evidence from visual framing of immigration in news stories
Source: PLoS One. 2025 Sep 15;20(9):e0331219. doi: 10.1371/journal.pone.0331219 (PMC12435698; doi:10.1371/journal.pone.0331219)
Supplement: S1 Appendix — (ZIP) [file pone.0331219.s001.zip › si_files/S10_Fig.pdf]

Fig. S.10: Visual frames and ideology of media outlets (only in 2018).

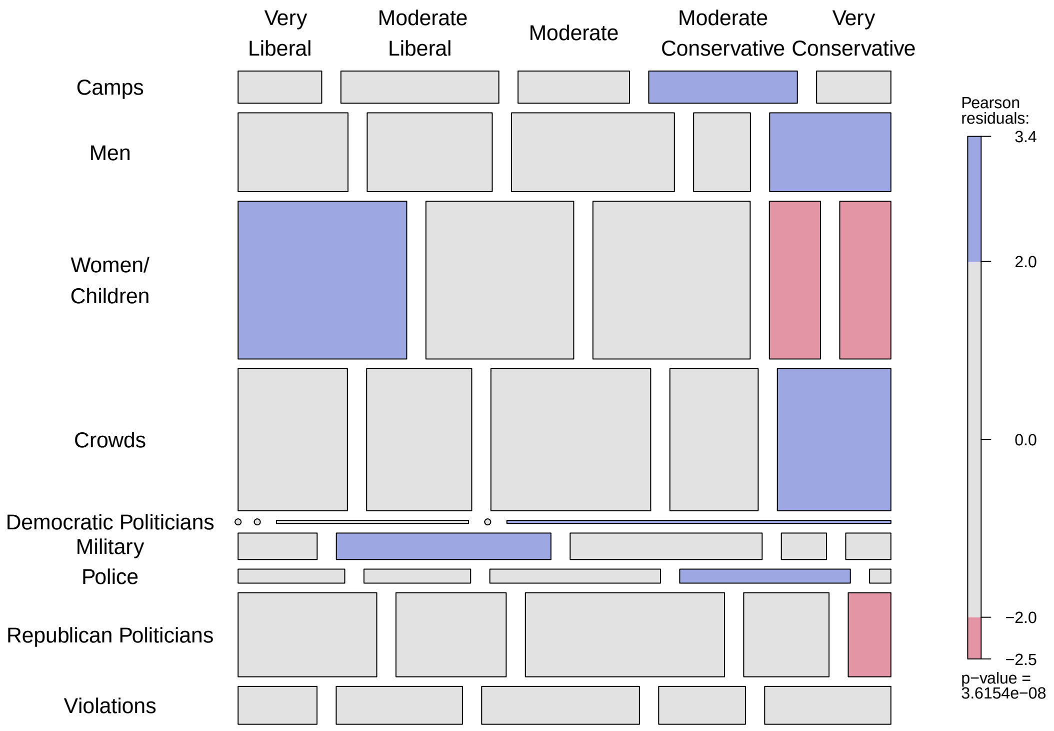

Note: This plot shows the relationship between two nominal variables of interest: (1) media outlet ideology (from very left-leaning to very right-leaning) and (2) image cluster. Colors indicate both the direction and strength of associations between categories. The strength of each association is measured by Pearson standardized residuals, which quantify how much observed counts deviate from those expected under independence. Blue shading denotes positive associations (more cases than expected), red shading denotes negative associations (fewer cases than expected), and gray indicates no meaningful association. The displayed p-value corresponds to a Chi-square test of independence and rejects the null hypothesis of no association between the two variables.
