# Supplementary material for: Media choice and audience perceptions: Evidence from visual framing of immigration in news stories
Source: PLoS One. 2025 Sep 15;20(9):e0331219. doi: 10.1371/journal.pone.0331219 (PMC12435698; doi:10.1371/journal.pone.0331219)
Supplement: S1 Appendix — (ZIP) [file pone.0331219.s001.zip › si_files/S2_Table.pdf]

Table S.2: Distribution of all pulled images across media outlets and years.

**Media Outlet/Year**	2017	2018	2019	2020	2021	**Total**
ABC News	0	26	0	0	0	26
AJ+	1	25	0	0	0	26
Al Jazeera English	0	13	1	5	10	29
American Greatness	0	1	0	0	0	1
Axios	0	2	0	0	0	2
azcentral	0	1	0	0	0	1
Block Club Chicago	0	2	0	0	0	2
Blue Lives Matter	0	4	0	0	0	4
Boston Herald	0	6	0	0	0	6
Breitbart News	0	1	0	0	0	1
BuzzFeed News	0	18	0	0	0	18
CBS News	0	53	9	0	0	62
Chicago Sun-Times	0	1	0	0	0	1
Chicago Tribune	0	19	0	0	0	19
CNN	0	58	3	0	0	61
CNN Business	0	1	0	0	0	1
Commercial Appeal	0	2	0	0	0	2
Conservative News	0	23	4	1	9	37
Courier Journal	0	3	0	0	0	3
Daily Caller	0	127	14	0	0	141
Daily Mail US	0	1	0	0	0	1
Daily Press	0	2	0	0	0	2
Daily Wire	0	21	0	4	4	29
Democracy Now!	0	11	0	0	0	11
DW News	0	1	0	0	0	1
East Bay Times	0	9	0	0	0	9
Esquire	0	0	1	0	0	1
Financial Times	0	1	0	0	0	1
FOX Business	0	4	0	0	0	4
Fox News	0	30	0	0	0	30
FrontPage Magazine	0	4	0	0	0	4
Gizmodo	0	2	0	0	0	2
GoLocalProv	0	1	0	0	0	1
GQ Magazine	0	3	0	0	0	3
heralddemocrat	0	1	0	0	0	1
HotAir.com	0	0	2	3	3	8
IndyStar	0	7	0	0	0	7
Jezebel	0	6	0	0	0	6
Judicial Watch	0	9	0	0	0	9
KATU News	0	5	0	0	0	5
KQED	0	4	1	0	0	5
L.A. Daily News	0	2	0	0	0	2
Las Vegas Sun	0	2	0	0	0	2
Law & Crime	0	4	0	0	0	4
LGBTQ Nation	0	7	0	0	0	7
Los Angeles Times	0	26	1	0	0	27
MarketWatch	0	5	0	0	0	5
Media Matters	0	9	3	0	4	16
Mediaite	0	17	0	0	0	17
MediaResearchCenter	0	10	1	0	0	11
Mercury News	0	4	0	0	0	4
Miami Herald	0	8	1	0	0	9
Military Times	0	3	0	0	0	3
Mother Jones	0	18	0	0	0	18
MSNBC	0	2	0	0	0	2
National Review	0	26	2	0	0	28
NBC News	0	13	0	0	0	13
New York Daily News	0	23	0	0	0	23
New York Post	0	25	1	2	14	42
New York Times Opinion	0	7	0	0	0	7

NewsBusters	0	11	1	0	0	12
Newsmax	0	2	0	1	0	3
Newsweek	0	122	2	0	0	124
NOLA.com	0	2	0	0	0	2
NPR	0	4	2	0	0	6
NY Post Opinion	0	3	0	0	0	3
Observer	0	1	0	0	0	1
One America News	0	16	9	3	2	30
PJ Media	0	1	0	0	0	1
POLITICO	0	6	0	0	0	6
PoliticusUSA	0	1	0	0	0	1
Rasmussen Reports	0	0	0	0	3	3
Record-Journal	0	1	0	0	0	1
Reuters	0	26	6	16	36	84
Rolling Stone	0	8	0	0	0	8
San Francisco Chronicle	0	2	1	0	0	3
SFGATE	0	7	0	0	0	7
Slate	0	57	0	0	0	57
South China Morning Post	0	1	0	0	0	1
Splinter	0	13	2	0	0	15
St. Louis Post-Dispatch	0	3	1	0	3	7
Star Tribune	0	8	1	0	0	9
Tennessean	0	1	0	0	0	1
Texas Tribune	0	9	12	0	0	21
The Babylon Bee	0	6	0	0	0	6
The Boston Globe	0	6	1	0	0	7
The Christian Science Monitor	0	1	0	0	0	1
The Daily Beast	0	8	0	0	0	8
The Daily Cardinal	0	1	0	0	0	1
The Daily Dot	0	9	2	0	0	11
The Daily Signal	0	8	1	0	0	9
The Desert Sun	0	16	0	0	0	16
The Detroit News	0	3	0	0	0	3
The Epoch Times	0	4	0	0	0	4
The Globe and Mail	0	16	0	0	0	16
The Guardian	0	1	0	0	0	1
The Hill	0	170	9	1	4	184
The Hollywood Reporter	0	5	0	0	0	5
The Japan Times	0	6	1	0	0	7
The Jerusalem Post	0	2	0	0	0	2
The New Republic	0	9	0	0	0	9
The New Yorker	0	23	0	0	0	23
The Oregonian	0	5	0	0	0	5
The Resurgent	0	4	0	0	0	4
The Root	0	1	0	0	0	1
The San Diego Union-Tribune	0	47	12	0	0	59
The Telegraph	0	1	0	0	0	1
The Verge	0	4	0	0	0	4
The Voice of America	0	6	0	0	0	6
The Washington Times	0	153	18	1	1	173
The Week	0	3	0	0	0	3
TheBlaze	0	3	0	0	0	3
ThinkProgress	0	76	0	0	0	76
TODAY	0	1	0	0	0	1
Truthout	0	3	0	0	0	3
U.S. News & World Report	0	12	1	0	0	13
UnionLeader.com	0	1	0	0	0	1
USA TODAY	0	13	0	0	0	13
Variety	0	1	0	0	0	1
Washington Examiner	0	29	0	0	0	29
WFAE	0	8	0	0	0	8
WGN TV News	0	4	0	0	0	4
Yahoo News	0	50	2	0	0	52

YES! Magazine	0	4	0	0	0	4

Table S.3: Distribution of images used in the survey wave across media outlets and years.

**Media Outlet/Year**	2017	2018	2019	2020	2021	**Total**
ABC News	0	1	0	0	0	1
AJ+	1	9	0	0	0	10
Al Jazeera English	0	2	1	2	2	7
American Greatness	0	1	0	0	0	1
Axios	0	1	0	0	0	1
azcentral	0	1	0	0	0	1
Boston Herald	0	1	0	0	0	1
CBS News	0	15	3	0	0	18
Chicago Tribune	0	5	0	0	0	5
CNN	0	7	0	0	0	7
Conservative News	0	2	1	1	2	6
Daily Caller	0	24	3	0	0	27
Daily Wire	0	3	0	1	2	6
Democracy Now!	0	5	0	0	0	5
DW News	0	1	0	0	0	1
East Bay Times	0	3	0	0	0	3
Fox News	0	1	0	0	0	1
FrontPage Magazine	0	1	0	0	0	1
Gizmodo	0	2	0	0	0	2
GoLocalProv	0	1	0	0	0	1
HotAir.com	0	0	0	0	1	1
IndyStar	0	2	0	0	0	2
KATU News	0	2	0	0	0	2
KQED	0	2	0	0	0	2
L.A. Daily News	0	1	0	0	0	1
Los Angeles Times	0	6	0	0	0	6
Mediaite	0	1	0	0	0	1
MediaResearchCenter	0	2	0	0	0	2
Military Times	0	2	0	0	0	2
Mother Jones	0	1	0	0	0	1
National Review	0	6	1	0	0	7
NBC News	0	5	0	0	0	5
New York Daily News	0	8	0	0	0	8
New York Post	0	6	1	0	3	10
Newsmax	0	1	0	0	0	1
Newsweek	0	31	0	0	0	31
NOLA.com	0	2	0	0	0	2
NY Post Opinion	0	2	0	0	0	2
One America News	0	8	4	1	0	13
PJ Media	0	1	0	0	0	1
POLITICO	0	1	0	0	0	1
PoliticusUSA	0	1	0	0	0	1
Reuters	0	5	2	6	10	23
Rolling Stone	0	2	0	0	0	2
San Francisco Chronicle	0	1	0	0	0	1
SFGATE	0	2	0	0	0	2
Slate	0	6	0	0	0	6
Splinter	0	1	0	0	0	1
St. Louis Post-Dispatch	0	2	0	0	1	3
Star Tribune	0	4	1	0	0	5
Texas Tribune	0	2	1	0	0	3
The Boston Globe	0	2	1	0	0	3
The Daily Beast	0	1	0	0	0	1
The Daily Signal	0	2	0	0	0	2
The Desert Sun	0	9	0	0	0	9
