# Supplementary material for: Media choice and audience perceptions: Evidence from visual framing of immigration in news stories
Source: PLoS One. 2025 Sep 15;20(9):e0331219. doi: 10.1371/journal.pone.0331219 (PMC12435698; doi:10.1371/journal.pone.0331219)
Supplement: S1 Appendix — (ZIP) [file pone.0331219.s001.zip › si_files/S9_Fig.pdf]

right-leaning media portray immigrants as 'crowds,' 'police,' and 'violations' rather than 'women and children'. They also use more images of politicians, both Republican and Democratic.

Fig. S.9: Visual frames and ideology of media outlets: Alternative image search.

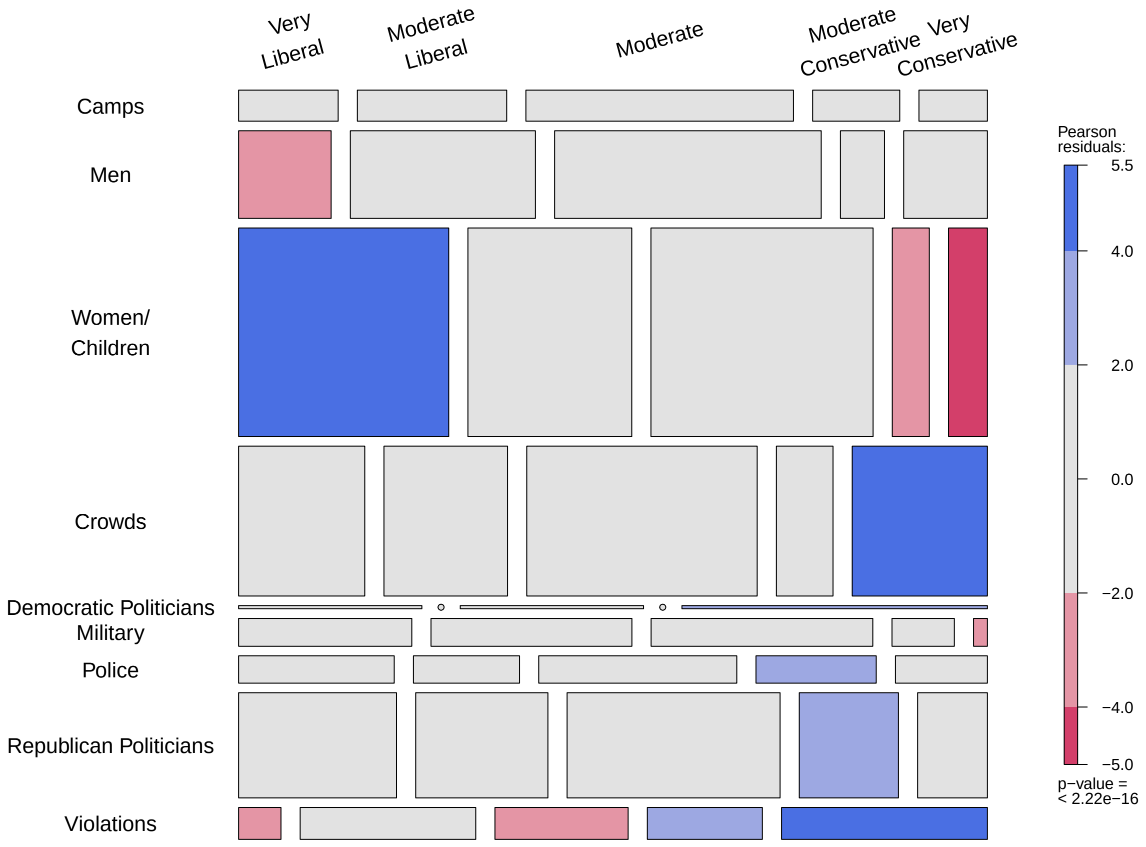

Note: This plot shows the relationship between two nominal variables of interest: (1) media outlet ideology (from very left-leaning/very liberal to very right-leaning/very conservative) and (2) image cluster. Colors indicate both the direction and strength of associations between categories. The strength of each association is measured by Pearson standardized residuals, which quantify deviations of observed counts from those expected under independence. Blue shading denotes positive associations (more cases than expected), red shading denotes negative associations (fewer cases than expected), and gray signifies no meaningful association. The p-value displayed corresponds to a Chi-square test of independence, rejecting the null hypothesis of no association between the two variables.
