# Supplementary material for: Media choice and audience perceptions: Evidence from visual framing of immigration in news stories
Source: PLoS One. 2025 Sep 15;20(9):e0331219. doi: 10.1371/journal.pone.0331219 (PMC12435698; doi:10.1371/journal.pone.0331219)
Supplement: S1 Appendix — (ZIP) [file pone.0331219.s001.zip › si_files/S28_Table.pdf]

Table S.28: Explanation of the label assignment with image examples.

<table>
<tr><th colspan="2">CATEGORY: CLOSE SHOTS (MEN)</th></tr>
<tr><td>

The category should be used for images depicting close-ups of men who are immigrants

</td><td>

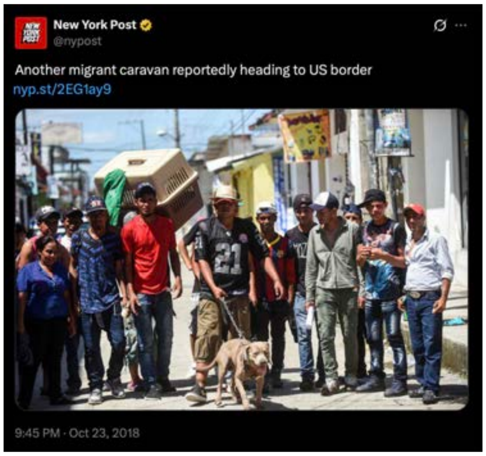

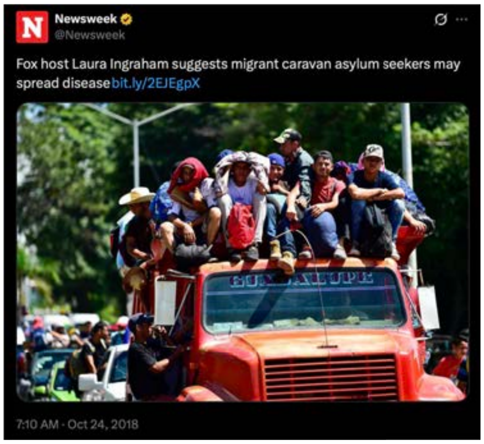

</td></tr>
<tr><td>

The image should be labeled with this category label if there are close-ups of men in a crowd (and you can easily identify people's faces).

Often you will see a crowd behind close shots of men (clearly distinguished facial features of men), label it as **close shots men** and not crowds.

</td><td>

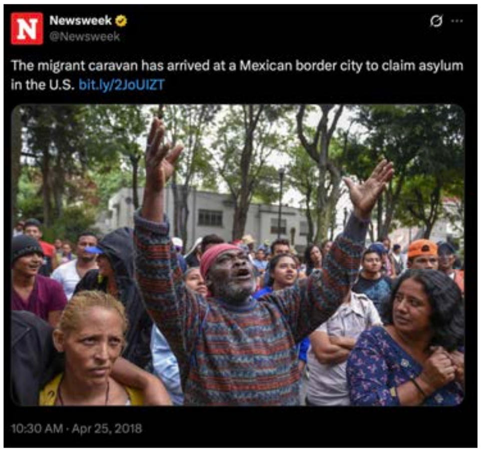

</td></tr>
</table>

<table>
<tr><td></td><td>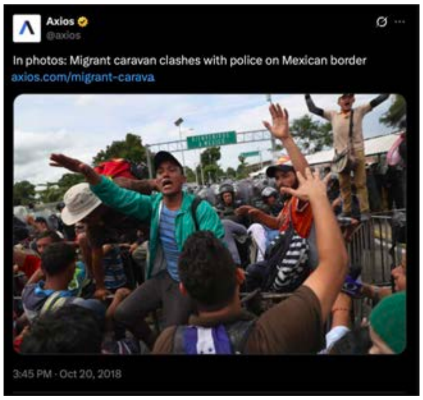
</td></tr>
<tr><td>If you see a man carrying a child, for example, think about what is the focus of the image - a man or a child - and assign an image to either this category or “Close shots: women and children”.</td><td>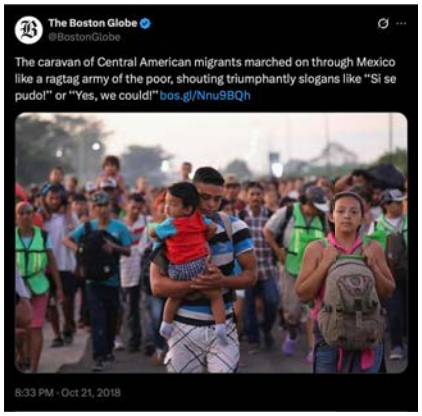
</td></tr>
<tr><td>If you see close shots of men and they wear police/military uniforms, they should be labeled “Police” or “Military” respectively. If an image depicts men with the signs “Policia Militar”, which is a special police force in Mexico, we agree to label them as police and not military.</td><td>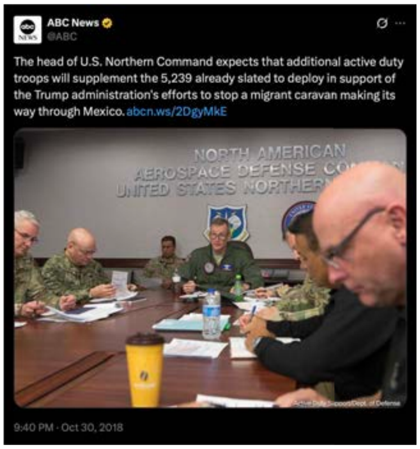
</td></tr>
</table>

If you see close-ups of men and they are not immigrants (e.g. they are journalists, actors, news figures, etc), put them in the **"Other"** category.

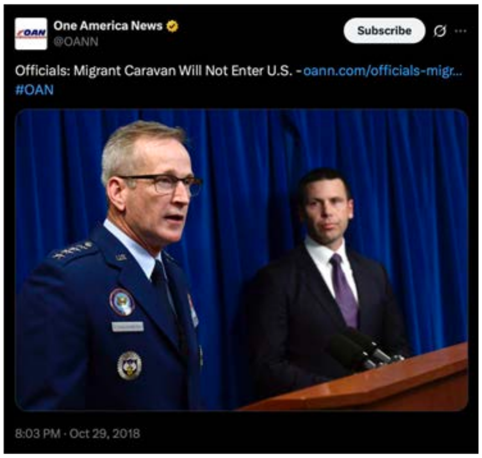

CATEGORY: CLOSE SHOTS (WOMEN AND CHILDREN)

The category should be used for images depicting close-ups of women and children who are immigrants.

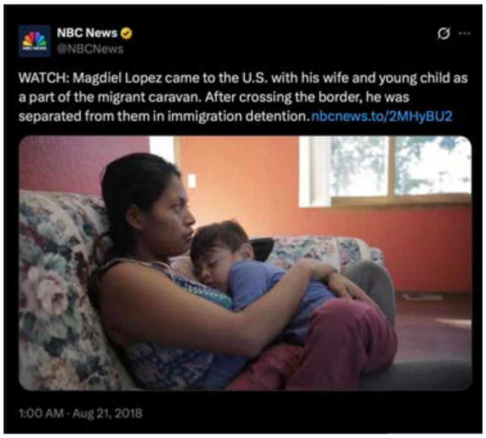

You can assign an image to "Close shots (women and children)" if you see a crowd with women and children in it and (1) can clearly see their faces or (2) they are in the focus of the image.

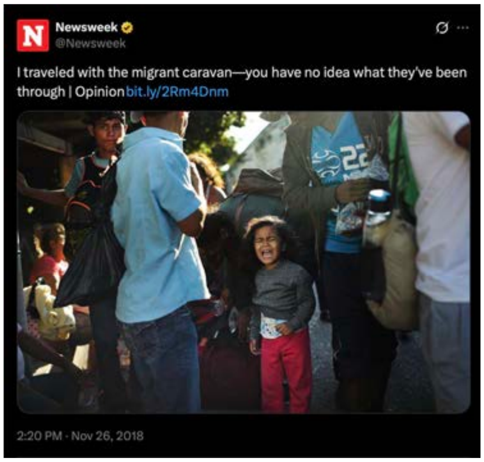

<table>
<tr>
<td></td>
<td>
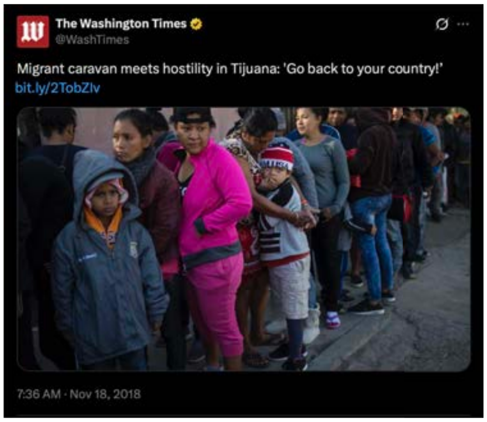

</td>
</tr>
<tr>
<td>
Consider the focus of your attention if you see a close-shot image with men, women, and children in the foreground.

Here, it seems that a man and a child dominate the picture, but you pay attention to the child in the first place, so it is “Close shots (women and children)”

Basically, close shots of children always override the portrayal of men.
</td>
<td>
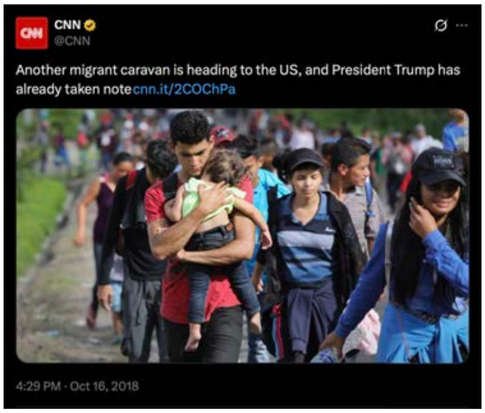

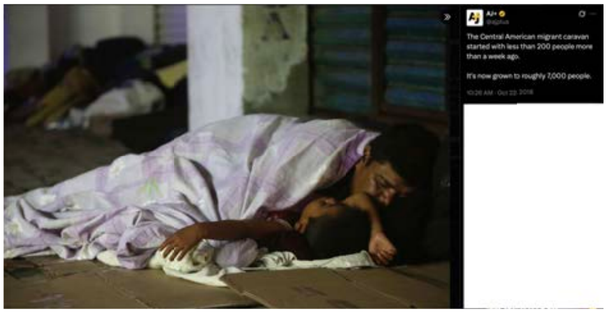

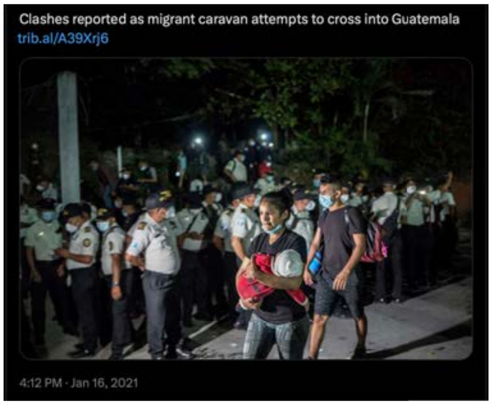

</td>
</tr>
</table>

<table>
<tr><td>The image should be assigned to ”Other” if it depicts a close-up of a woman who is likely to be a politician/reporter/host.</td><td>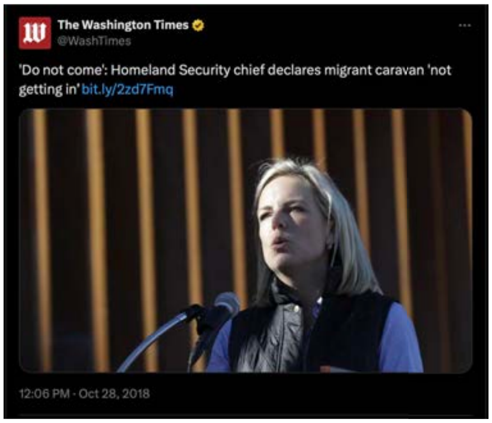
</td></tr>
<tr><td colspan="2">CATEGORY: CROWDS</td></tr>
<tr><td>Images under the “Crowds” label depict a crowd and the faces of people are either unidentifiable or they are mixed-gender barely identifiable, so there is no clear accent on a close shot of a man/woman/children.</td><td>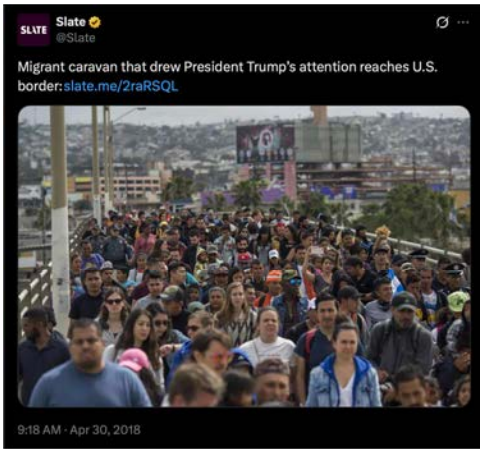
</td></tr>
<tr><td colspan="2">CATEGORY: REPUBLICAN POLITICIANS</td></tr>
<tr><td>Images that portray Donald Trump, Mike Pence, or Ted Cruz</td><td>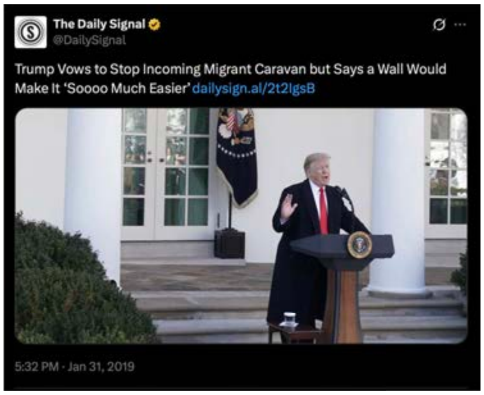
</td></tr>
</table>

Images with recognizable republican politicians that contain words, which take up less than 30% of the image	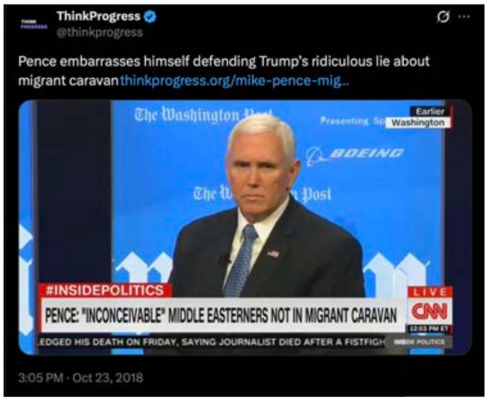
Images that might contain crowd but a Republican politician is the main focus	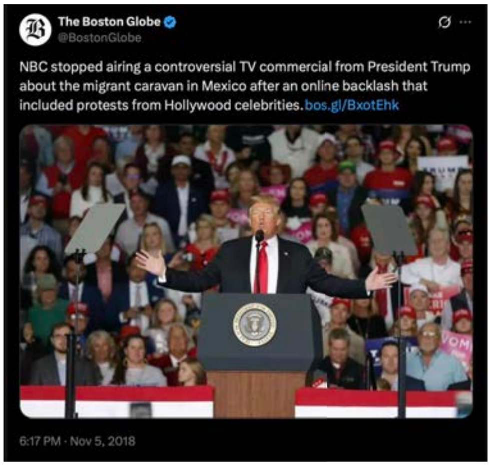<br>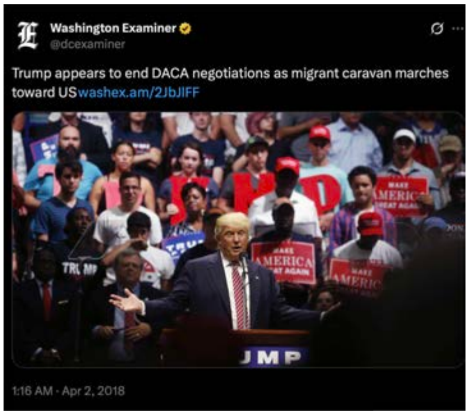

CATEGORY: DEMOCRATIC POLITICIANS

Images with close-ups of Joe Biden, Kamala Harris or Alexandria Ocasio-Cortez, and Elizabeth Warren.

Similar to the category of Republican politicians: images with recognizable republican politicians that contain words, which take up less than 30% of the image; and images that might contain crowds but a Democratic politician is the main focus

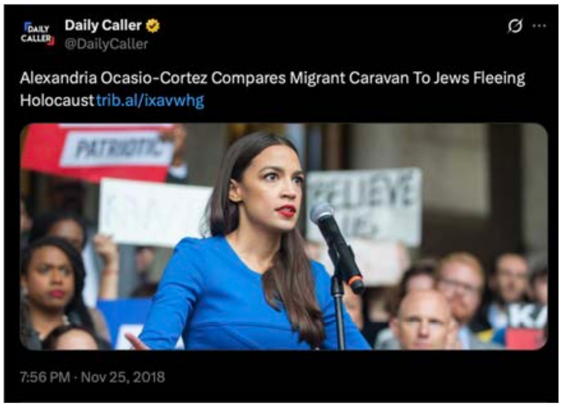

CATEGORY: VIOLATIONS (VIOLENCE/CONFLICT/TRASSPASSING/CLASHES)

If an image depicts a clear violation of the law/norms/rules, a clash/conflict either between immigrants or between immigrants and police, it should be labeled with **'Violations'**.

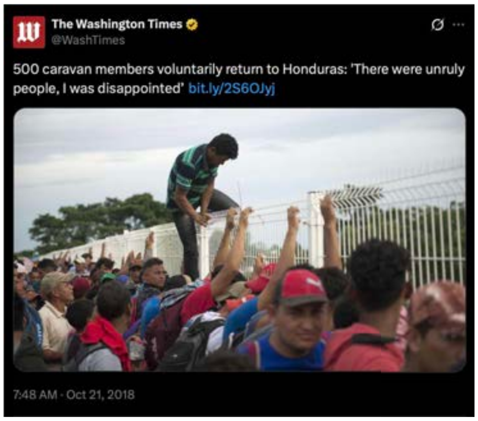

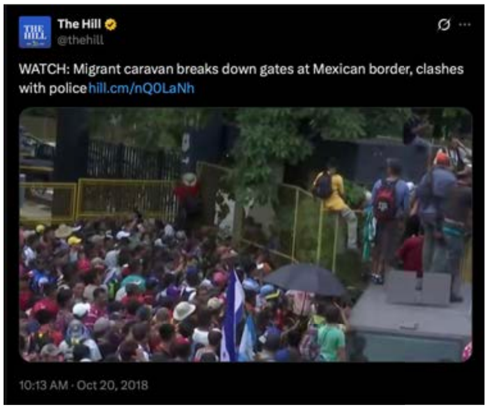

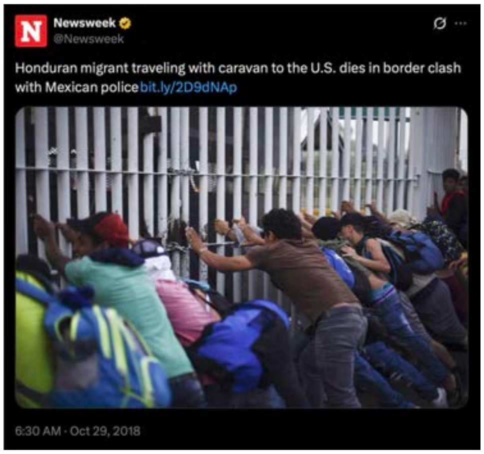

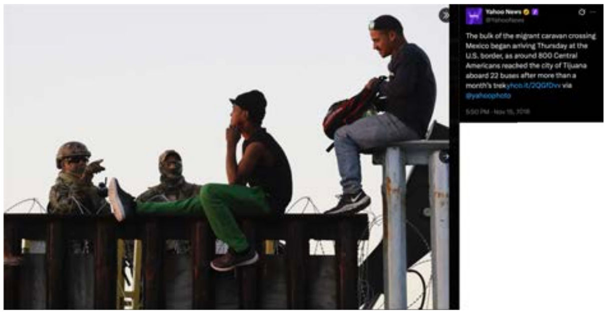

Some images depict "Military" but also "Crowd" and "Violation" and "Close shot (men)", again, think about the focus of the image.

If you see a crowd of immigrants fighting the police crowd, it should be labeled not as crowd, but as **"Violation"**.

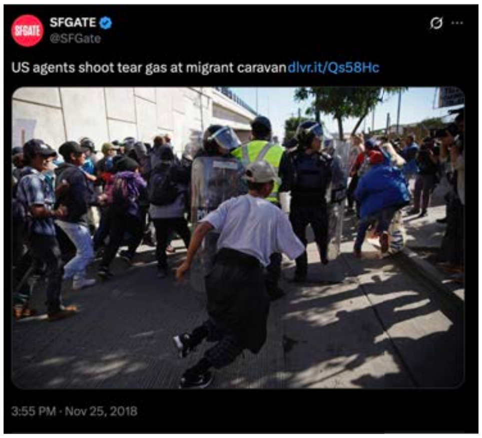

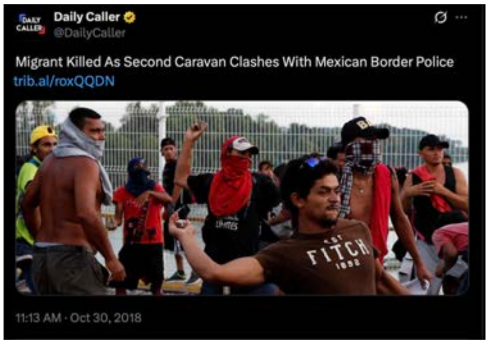

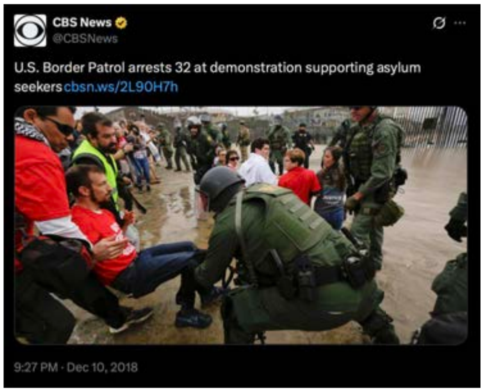

CATEGORY: CAMPS

"Camps" depict images of migrant caravan camps/shelters/refuge.

Occasionally, images of camps may contain close-ups. Think about the focus of the image here and label it either "Camps" or "Close shot (men)" or "Close shot (women and children)".

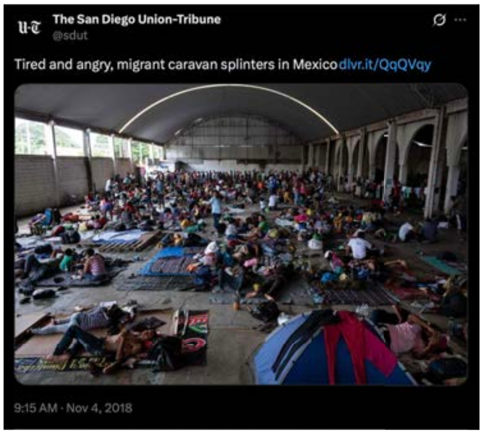

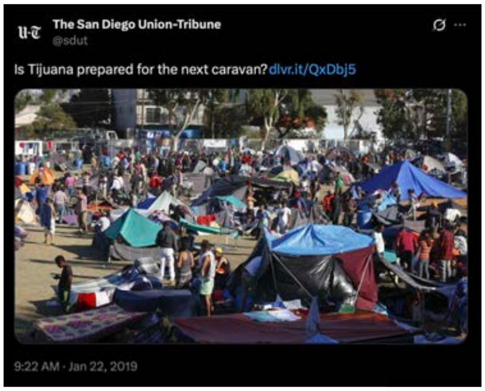

<table>
<tr><td></td><td>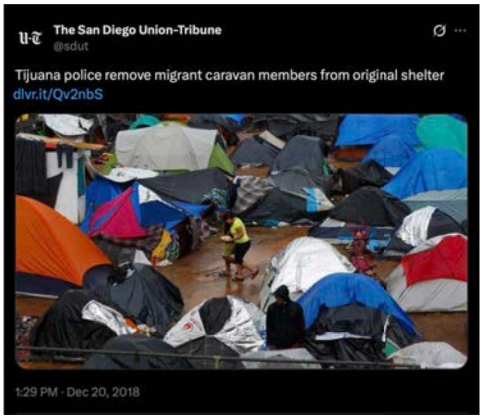
</td></tr>
<tr><td>Sometimes camps contain only images of tents, and no people portrayed at those images, label them as “Camps” as well.</td><td>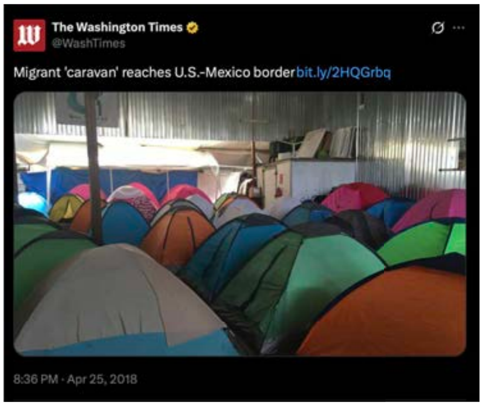
</td></tr>
<tr><td colspan="2">CATEGORY: MILITARY</td></tr>
<tr><td>Images that depict the military should be labeled with “Military”.

Hint: You can usually distinguish military from police by the uniforms. Sand-greenish colors are military colors, and blue/black are police colors. You can also read the name tags military vs police.</td><td>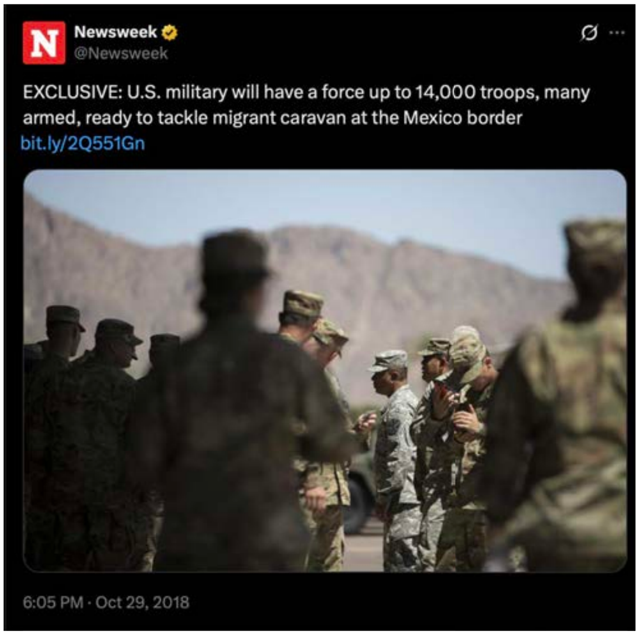
</td></tr>
</table>

If an image depicts men with the signs “Policia Militar”, which is a special police force in Mexico, we agree to label them as a **police** and not military.

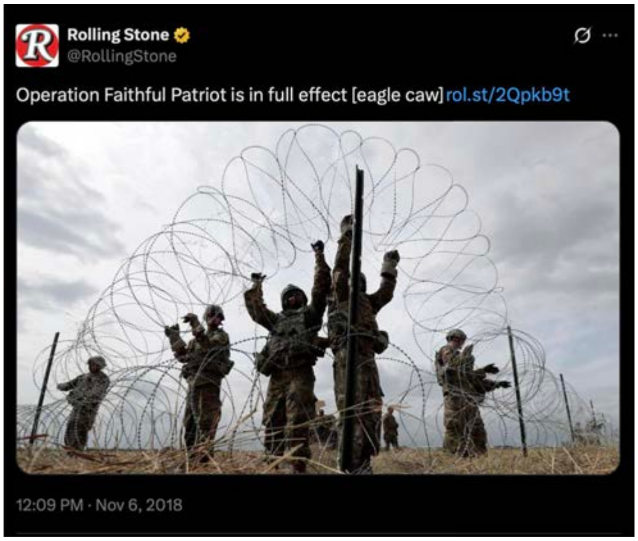

CATEGORY: POLICE

If an image depicts police it should be labeled with the “Police” label.

Hint: You can usually distinguish police from military by the uniforms. Sand-greenish colors are military colors, and blue/black are police colors. You can also read the name tags military vs police.

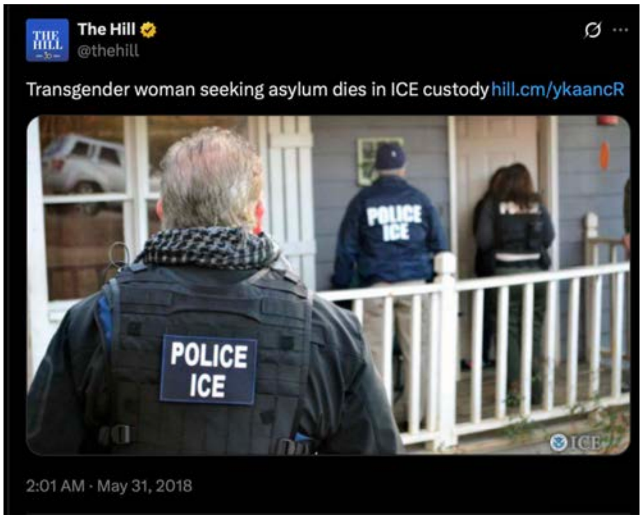

If an image depicts men with the signs “Policia Militar”, which is a special police force in Mexico, we agree to label them as a **police** and not military.

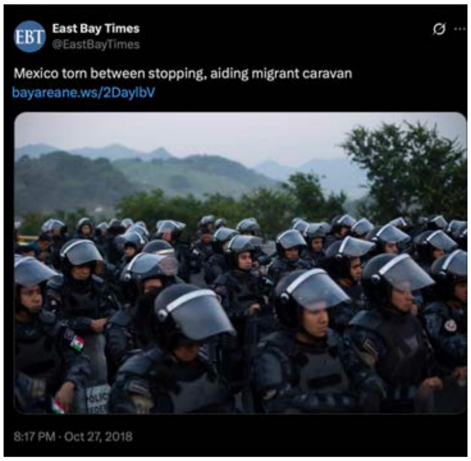

CATEGORY: OTHER

This is a residual category. If you feel that none of the labels fit what you see on the image, please assign it to the“**Other**”label category.

Images that contain text that take up MOST OF THE IMAGE.

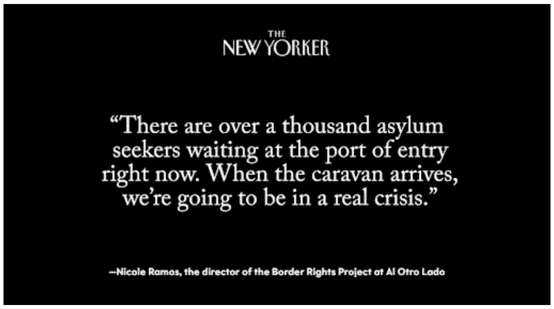

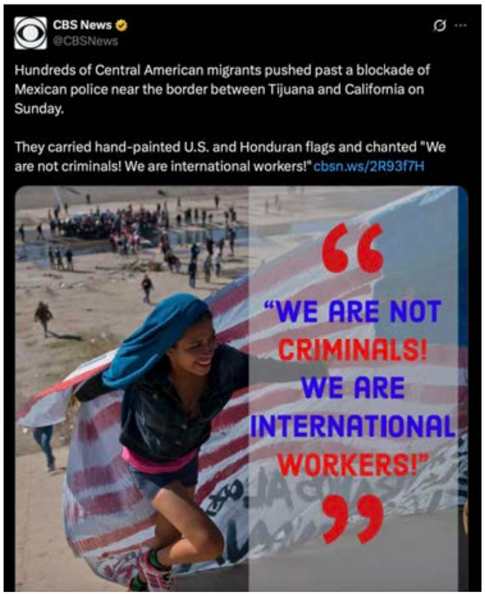

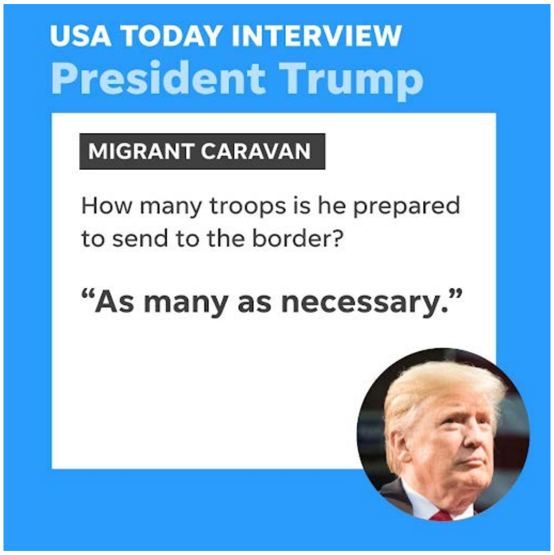

PETE HEGSETH (HOST, *FOX & FRIENDS WEEKEND*): The laws are, as the president calls them, dumb. They don't make sense. They don't protect our sovereignty. That's where you have to go back to Congress and say, it's about time we actually do something about it. A wall is part of saying, you don't get to come into our country unless you prove that you are a refugee. So, go to a port of entry, claim asylum, you'll get your day in court, we'll determine whether it's valid or not. But, you got the president of Guatemala saying to a local newspaper down there just last week, they caught over a hundred ISIS fighters in Guatemala trying to use this caravan or other processes --

STEVE DOOCY (CO-HOST): Are we sure that's true?

HEGSETH: He talked to their local newspaper, we don't know it, it hasn't been verified. But even one poison pill is too many in a caravan.

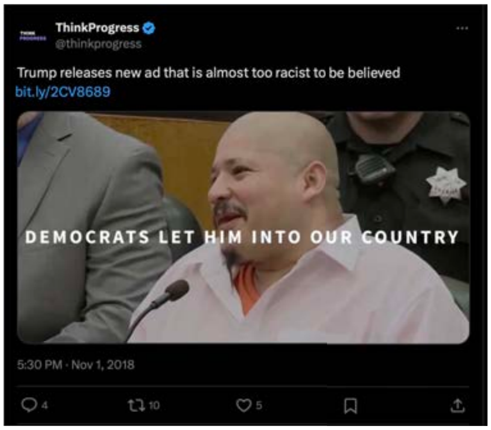

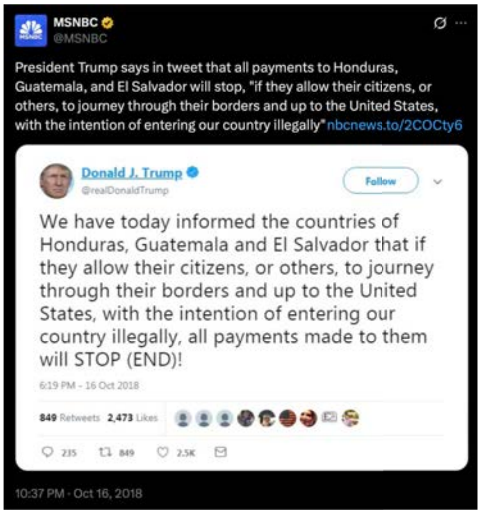

Images that contain several images or several political figures.

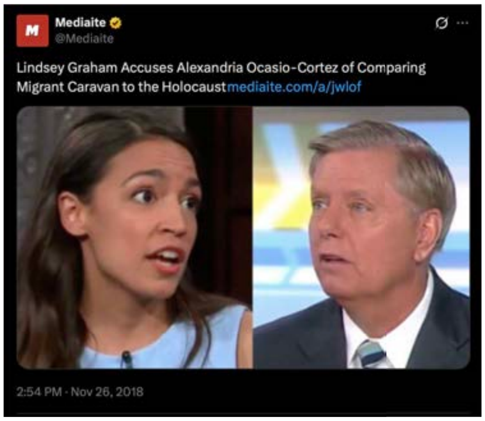

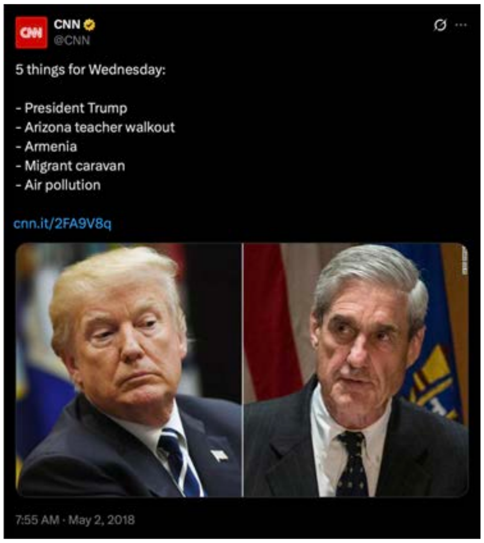

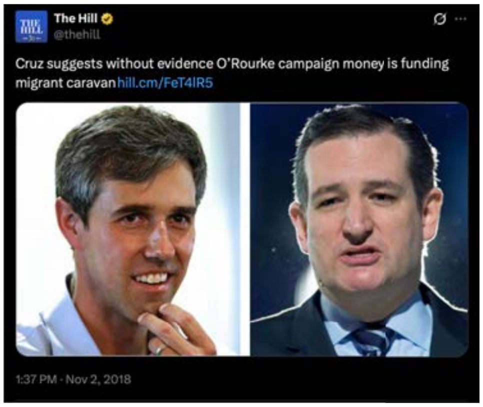

Images that contain labels (e.g. of a news channel) that take up MOST OF THE IMAGE

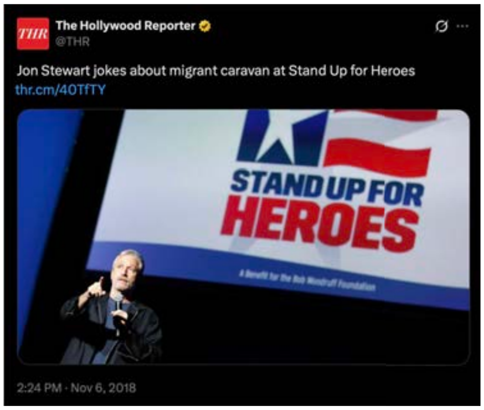

<table>
<tr><td></td><td>TIME</td></tr>
<tr><td>Hard-to-label images that contain objects from several categories or none of the categories.</td><td>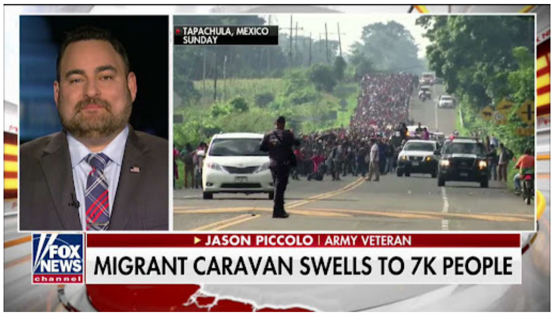

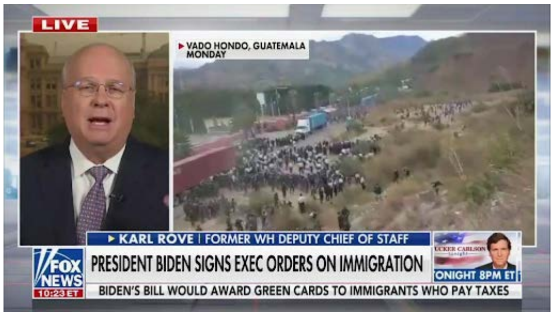

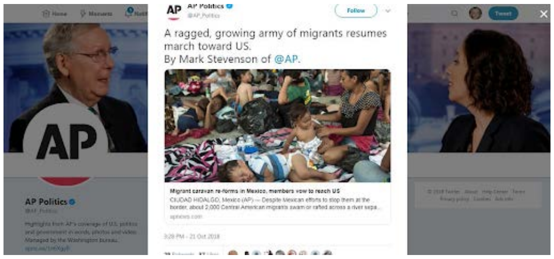

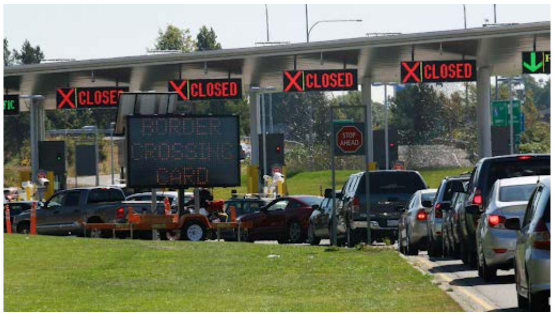
</td></tr>
</table>

Category “Other” will include images of all the politicians that are not the following ones:	
Joe Biden. Should be labeled as **“Democratic politicians”**.	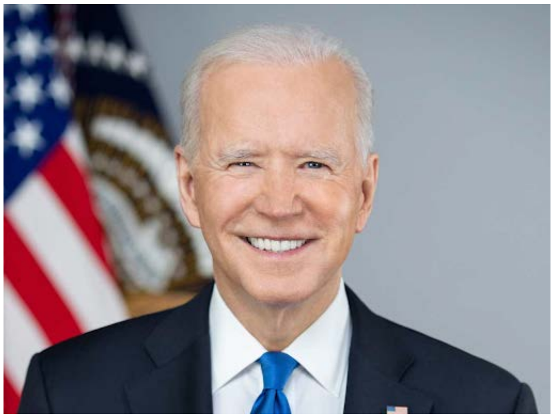
Kamala Harris. Should be labeled as **“Democratic politicians”**.	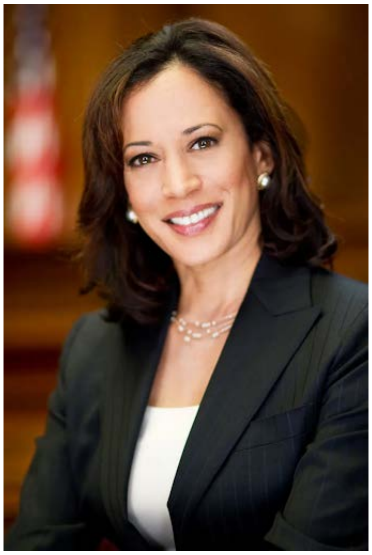
Alexandria Ocasio-Cortez. Should be labeled as **“Democratic politicians”**.	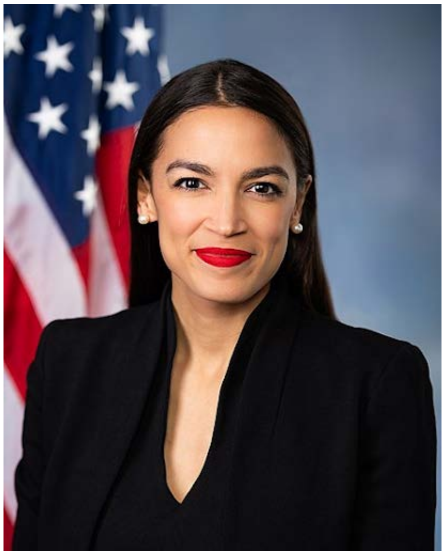

Elizabeth Warren. Should be labeled as **“Democratic politicians”**.	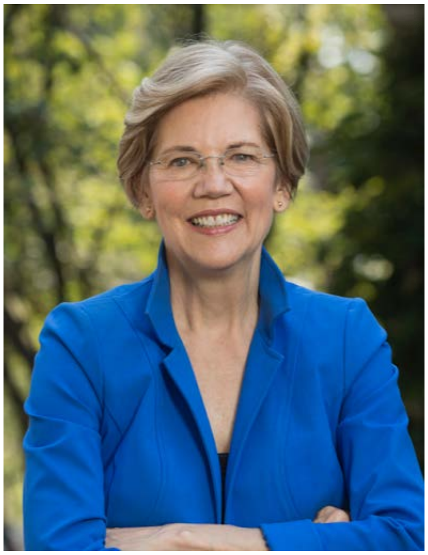
Donald Trump. Should be labeled as **“Republican politicians”**.	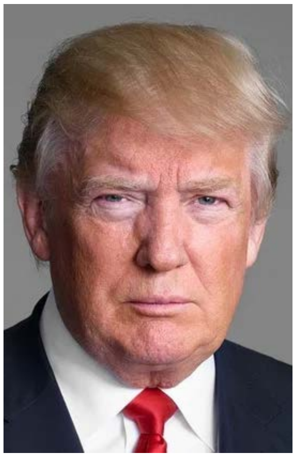
Mike Pence. Should be labeled as **“Republican politicians”**.	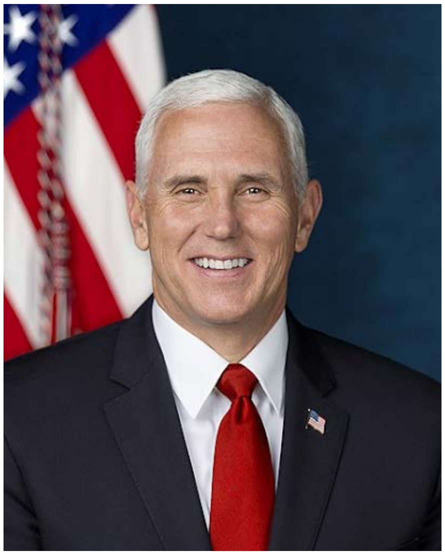

Ted Cruz. Should be labeled as **"Republican politicians"**.	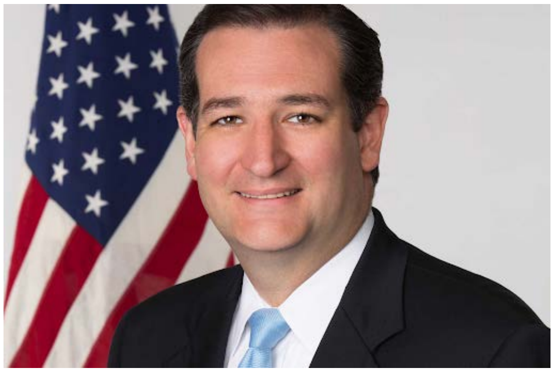
