# Supplementary material for: Media choice and audience perceptions: Evidence from visual framing of immigration in news stories
Source: PLoS One. 2025 Sep 15;20(9):e0331219. doi: 10.1371/journal.pone.0331219 (PMC12435698; doi:10.1371/journal.pone.0331219)
Supplement: S1 Appendix — (ZIP) [file pone.0331219.s001.zip › si_files/S22_Table.pdf]

S13 Comparison of High- and Low-Confidence Responses for Accuracy and Outlet Ideology Guesses

Table S.22: Distribution of response confidence for accuracy and outlet ideology guesses.

Confidence	Accuracy	Ideology
1	267	308
2	266	280
3	508	565
4	2128	2205
5	1842	1738
6	1374	1277
7	3411	3091
