# Supplementary material for: Media choice and audience perceptions: Evidence from visual framing of immigration in news stories
Source: PLoS One. 2025 Sep 15;20(9):e0331219. doi: 10.1371/journal.pone.0331219 (PMC12435698; doi:10.1371/journal.pone.0331219)

Fig. S.2: Number of images used in tweets across media outlets with different ideological standpoints.

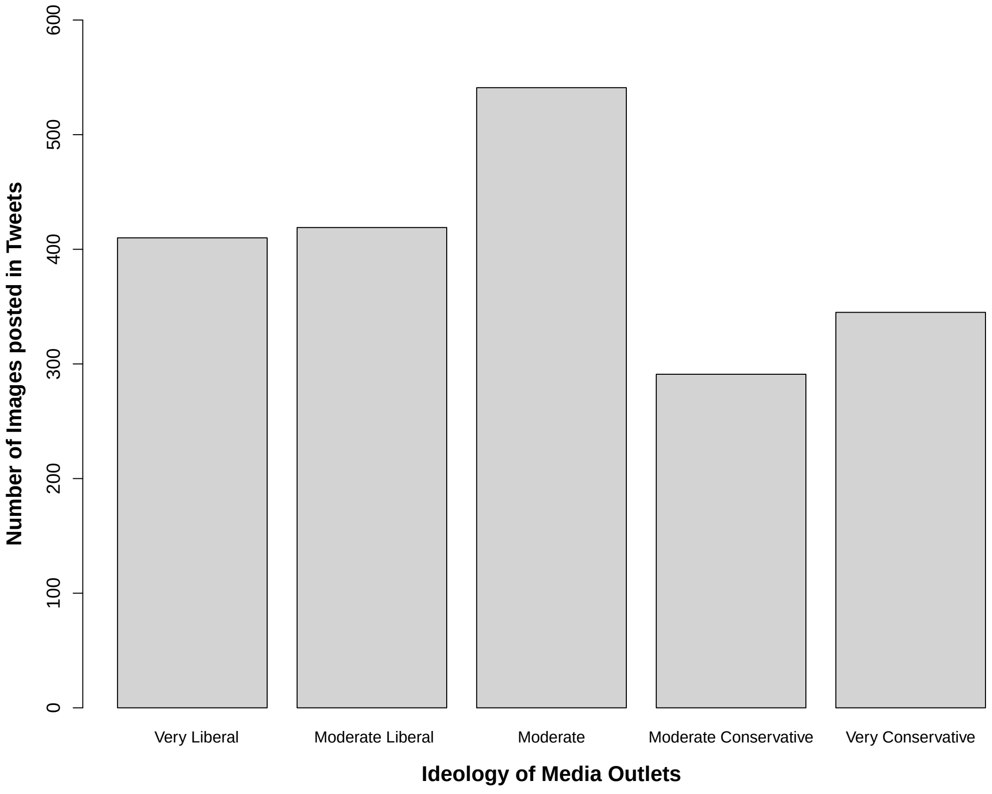

Supplement: S1 Appendix — (ZIP) [file pone.0331219.s001.zip › si_files/S2_Fig.pdf]
