# Supplementary material for: Media choice and audience perceptions: Evidence from visual framing of immigration in news stories
Source: PLoS One. 2025 Sep 15;20(9):e0331219. doi: 10.1371/journal.pone.0331219 (PMC12435698; doi:10.1371/journal.pone.0331219)
Supplement: S1 Appendix — (ZIP) [file pone.0331219.s001.zip › si_files/S13_Appendix.pdf]

# S13 Comparison of High- and Low-Confidence Responses for Accuracy and Outlet Ideology Guesses

**Table S.22: Distribution of response confidence for accuracy and outlet ideology guesses.**

| Confidence | Accuracy | Ideology |
|---|---|---|
| 1 | 267 | 308 |
| 2 | 266 | 280 |
| 3 | 508 | 565 |
| 4 | 2128 | 2205 |
| 5 | 1842 | 1738 |
| 6 | 1374 | 1277 |
| 7 | 3411 | 3091 |

**Fig. S.12: Accuracy results for high-confidence respondents.**

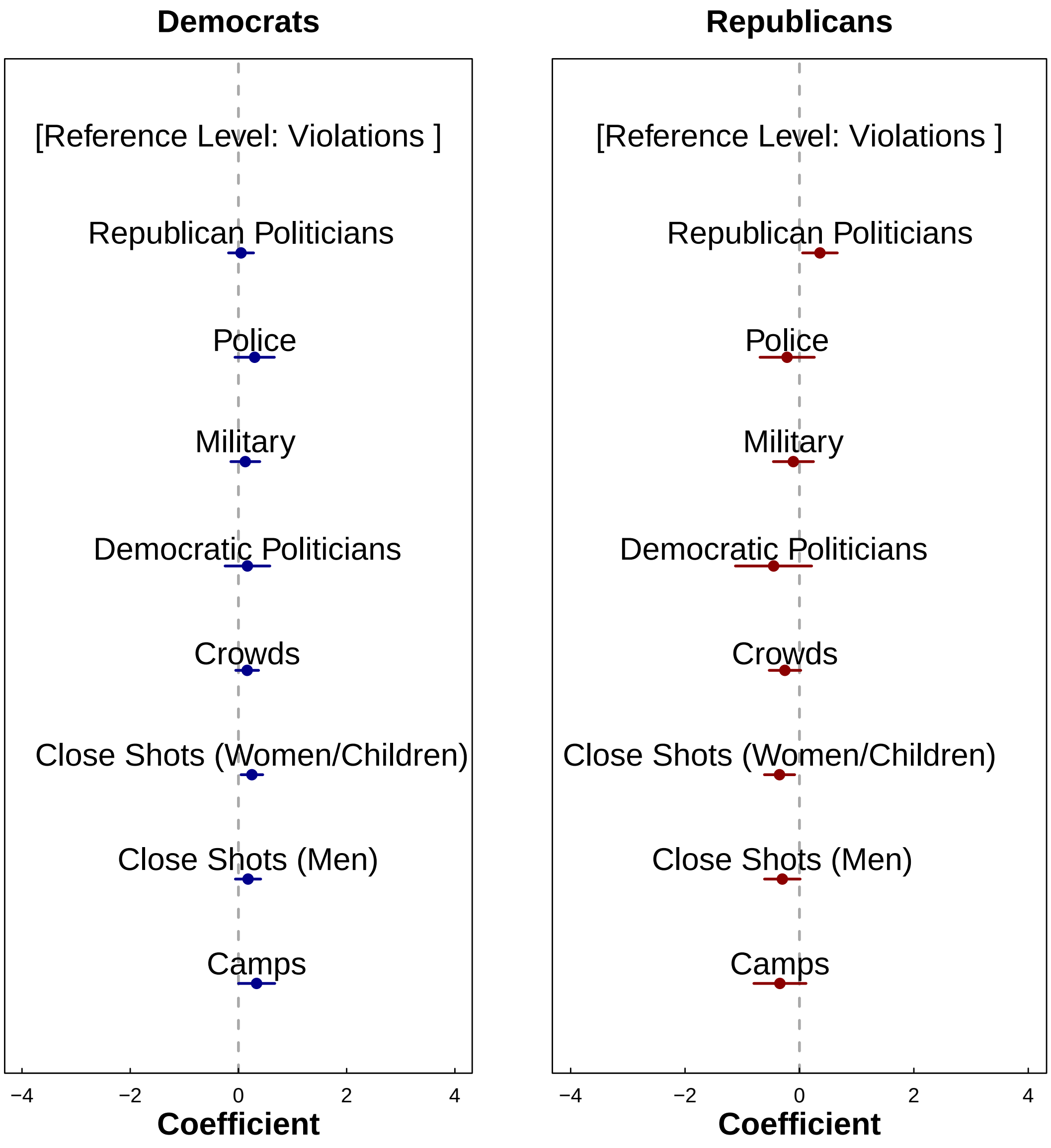


*Note:* The plot presents results from two linear models with random effects (random intercepts for images and respondents): one for Democratic respondents and one for Republican respondents. Models include only respondents who reported high confidence in their answers (ratings more than 4 on the 7-point scale). Each line shows a regression coefficient (with 95% CI) for visual-frame predictors, using the "Violations" cluster as the reference. Accuracy was measured on a 7-point scale ranging from 1 (image gives a faulty representation) to 7 (image gives an accurate representation). Both models control for gender, age, ethnicity, income, education, and interest in politics.

**Fig. S.13: Accuracy results for low-confidence respondents.**

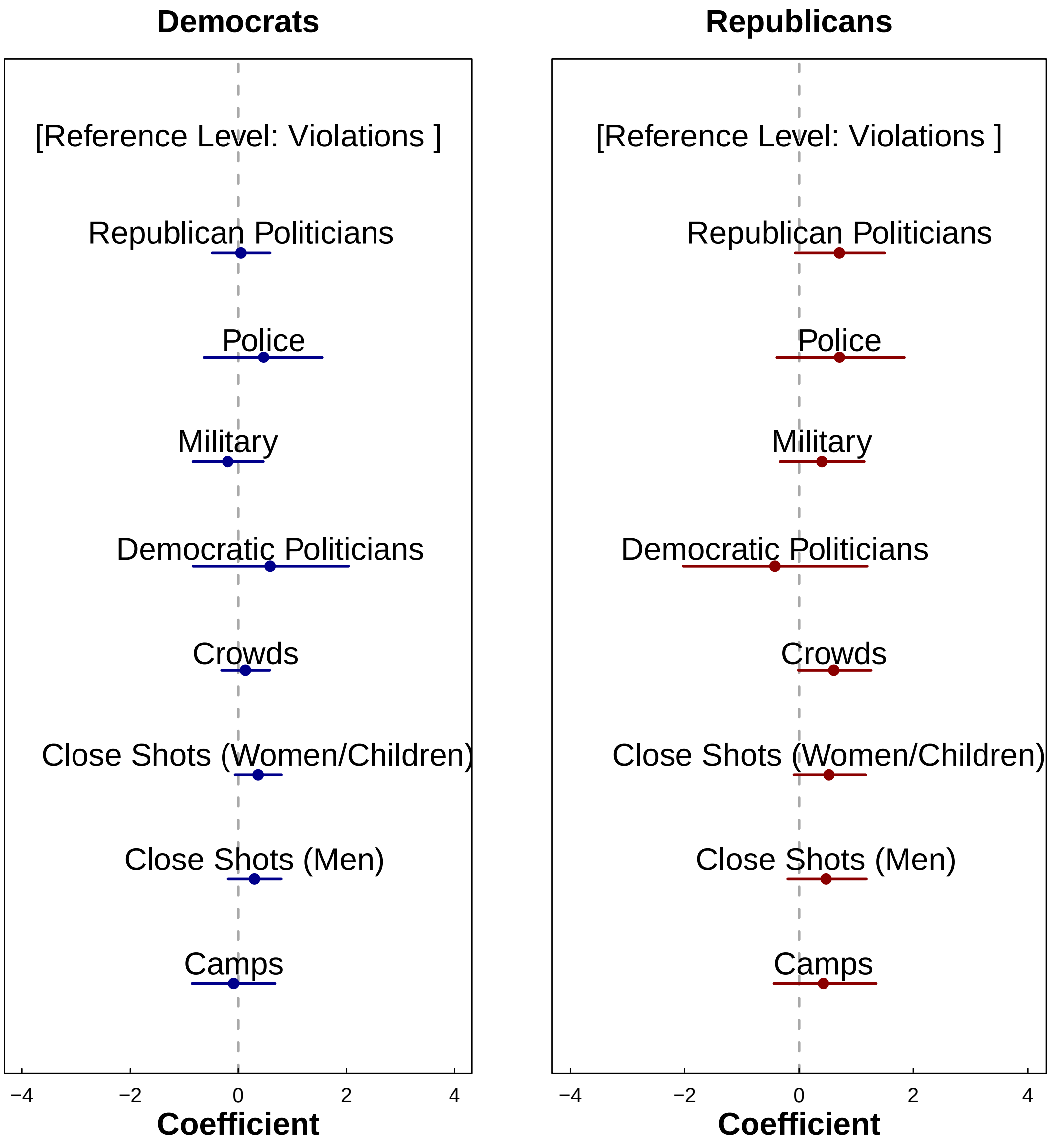


*Note:* The plot presents results from two linear models with random effects (random intercepts for images and respondents): one for Democratic respondents and one for Republican respondents. Each model includes only respondents reporting low confidence (ratings less than 4 on the 7-point scale). Each line shows a regression coefficient (with 95% CI) for visual-frame predictors, using the "Violations" cluster as the reference. Accuracy was measured on a 7-point scale ranging from 1 (image gives a faulty representation) to 7 (image gives an accurate representation). Both models control for gender, age, ethnicity, income, education, and interest in politics.

**Fig. S.14: Media outlet ideology guesses for high-confidence respondents.**

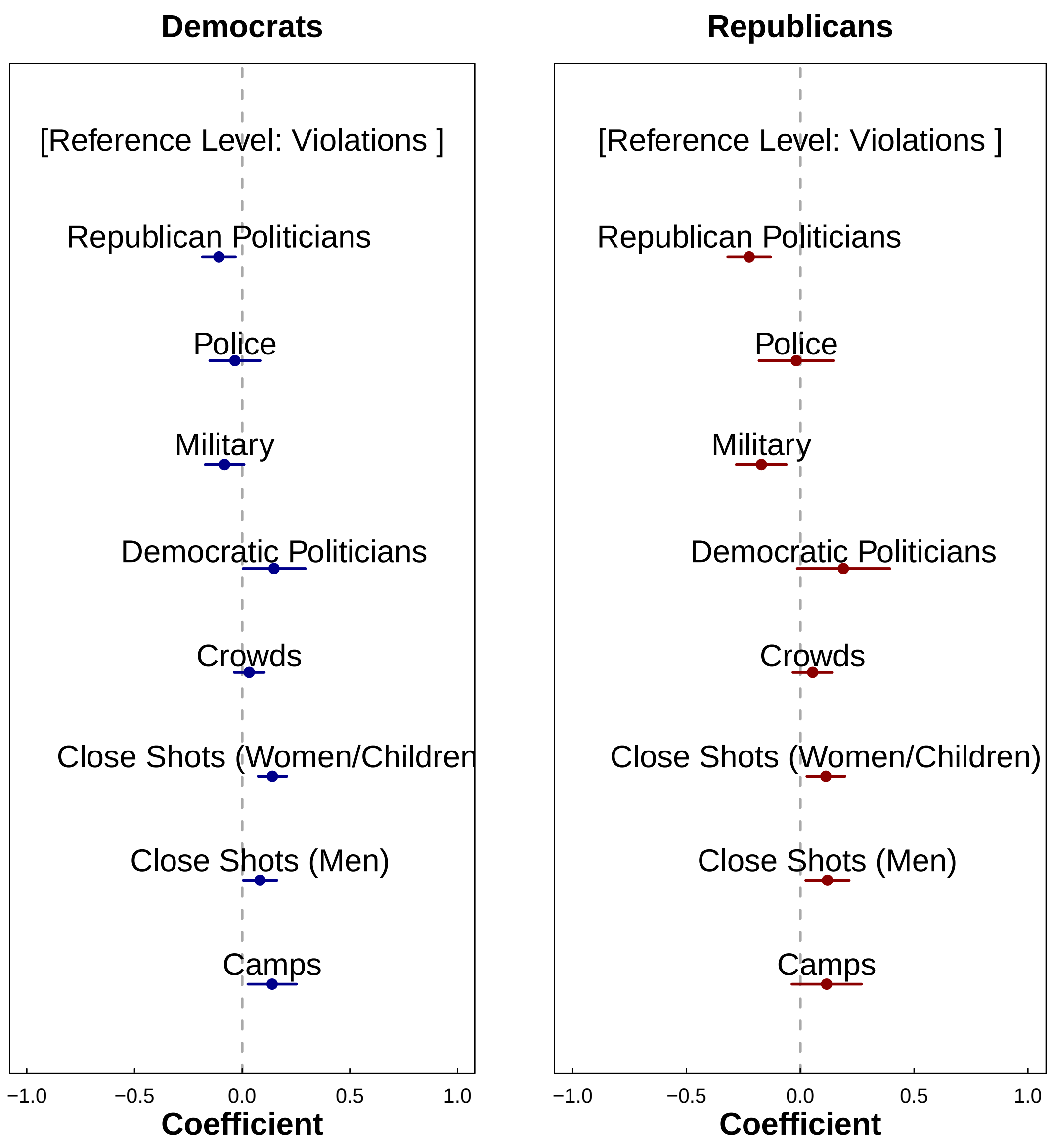


*Note:* The plot presents results from two linear models with random effects (random intercepts for images and respondents): one for Democratic respondents and one for Republican respondents. Each model includes only respondents reporting high confidence (ratings more than 4 on the 7-point scale). Each line shows an estimated regression coefficient (with 95% CI) for visual-frame predictors, with the "Violations" cluster as the reference. The outcome is a binary indicator (1 = liberal outlet guess; 0 = conservative outlet guess). Both models control for gender, age, ethnicity, income, education, and interest in politics.

**Fig. S.15: Media outlet ideology guesses for low-confidence respondents.**

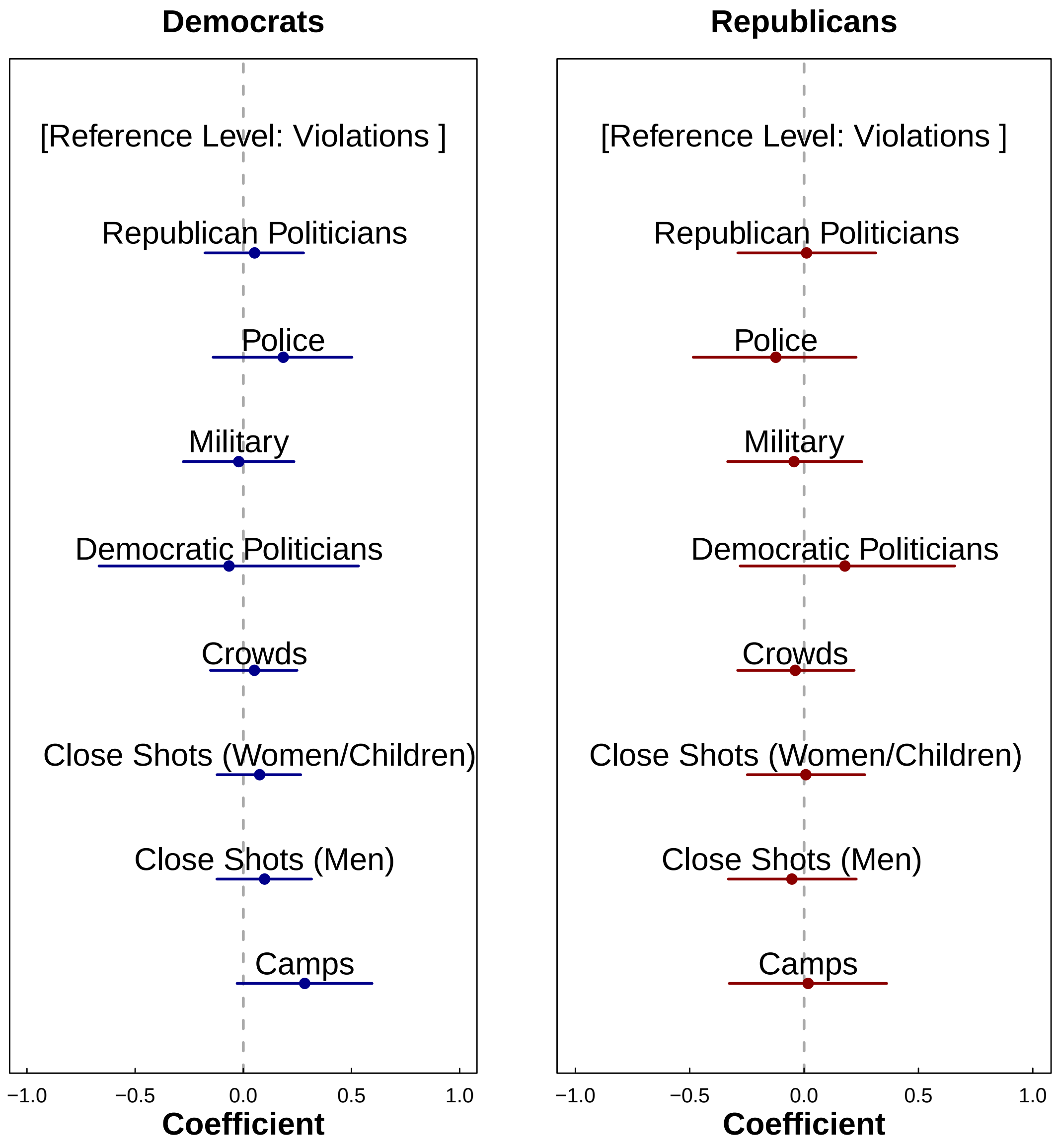


*Note:* The plot presents results from two linear models with random effects (random intercepts for images and respondents): one for Democratic respondents and one for Republican respondents. Each model includes only respondents reporting low confidence (ratings less than 4 on the 7-point scale). Each line shows an estimated regression coefficient (with 95% CI) for visual-frame predictors, using the "Violations" cluster as the reference. The outcome is a binary indicator (1 = liberal outlet guess; 0 = conservative outlet guess). Both models control for gender, age, ethnicity, income, education, and interest in politics.
