# Supplementary material for: Media choice and audience perceptions: Evidence from visual framing of immigration in news stories
Source: PLoS One. 2025 Sep 15;20(9):e0331219. doi: 10.1371/journal.pone.0331219 (PMC12435698; doi:10.1371/journal.pone.0331219)
Supplement: S1 Appendix — (ZIP) [file pone.0331219.s001.zip › si_files/S5_Table.pdf]

S5.2 Alternative Search of Images

We also need to check whether our results are robust to other images on a similar topic. Our initial set of images have been shaped by querying tweets mentioning "migrant caravan" connotation. We now narrow the search timeline by looking at 3 months periods around first (February 15, 2018 - May 15, 2018) and second caravans (September 15, 2018 - December 15, 2018) - the most prominent caravans. Additionally, instead of using "migrant caravan" connotation, we queried tweets and corresponding images based on the appearance of any of the following words in tweets: "migrant," "caravan," "migrants," "caravans". Such a query results in 14594 tweets (excluding duplicates of the tweets for each of the keyword search) and 5149 images that accompany these tweets. Table S.5 summarizes the returned results for the collected tweets:

Table S.5: Returned tweets for each of the key words searches.

Term	Count
migrant	10,163
migrants	5,194
caravan	7,086
caravans	447
migrant caravan	3,060

We ask trained coders to label these images using our curated codebook and assign one of 10 labels to all the images. We use the majority rule intercoders agreement to assign final labels to each of the images. If there is a lack of agreement between the coders, we leave images as unlabelled and exclude them from the final analysis.

We estimate a association between media outlets ideology and image labels for all images with meaningful labels (excluding the "Other" category and images with no agreement on labels; total of 2447 images). The results of this association are shown in Figure S.9. Not only do they support the original findings, but they also amplify them. Here we observe that left-leaning outlets tend to use frames of 'women and children' significantly more often than 'men,' and 'violations,' which they tend to ignore. In contrast,
