# Supplementary material for: Media choice and audience perceptions: Evidence from visual framing of immigration in news stories
Source: PLoS One. 2025 Sep 15;20(9):e0331219. doi: 10.1371/journal.pone.0331219 (PMC12435698; doi:10.1371/journal.pone.0331219)

The Globe and Mail	0	6	0	0	0	6
The Guardian	0	1	0	0	0	1
The Hill	0	19	1	0	1	21
The Hollywood Reporter	0	1	0	0	0	1
The Japan Times	0	1	0	0	0	1
The New Republic	0	1	0	0	0	1
The Oregonian	0	3	0	0	0	3
The San Diego Union-Tribune	0	5	3	0	0	8
The Voice of America	0	4	0	0	0	4
The Washington Times	0	14	2	0	0	16
ThinkProgress	0	1	0	0	0	1
Truthout	0	2	0	0	0	2
U.S. News & World Report	0	3	0	0	0	3
USA TODAY	0	1	0	0	0	1
Washington Examiner	0	2	0	0	0	2
WGN TV News	0	1	0	0	0	1
Yahoo News	0	9	1	0	0	10
YES! Magazine	0	1	0	0	0	1

S2 “Migrant Caravans” is Equally Present in Liberal and Conservative Media Outlets

It is possible that Republicans use the expression ”migrant caravan” more frequently than Democrats, so the word choice we have made might have influenced the structure of the image sample we use. By searching social media content with only one textual connotation, some information may not be retrieved. We show in Figure S.1 that this intuition is not supported. The term ”migrant caravan” is equally frequently used by left- and right-leaning media outlets, so our main predictor has no effect on it. In Figure S.2 it is shown that left-leaning and right-leaning media outlets are equally likely to attach images to tweets, indicating that there is no systematic bias in the frequency with which left- and right-leaning media use the term ”migrant caravans” to discuss immigration, and these media are equally likely to attach images to these stories.

Fig. S.1: Distribution of tweets mentioning "migrant caravans" across media outlets with different ideological standpoints.

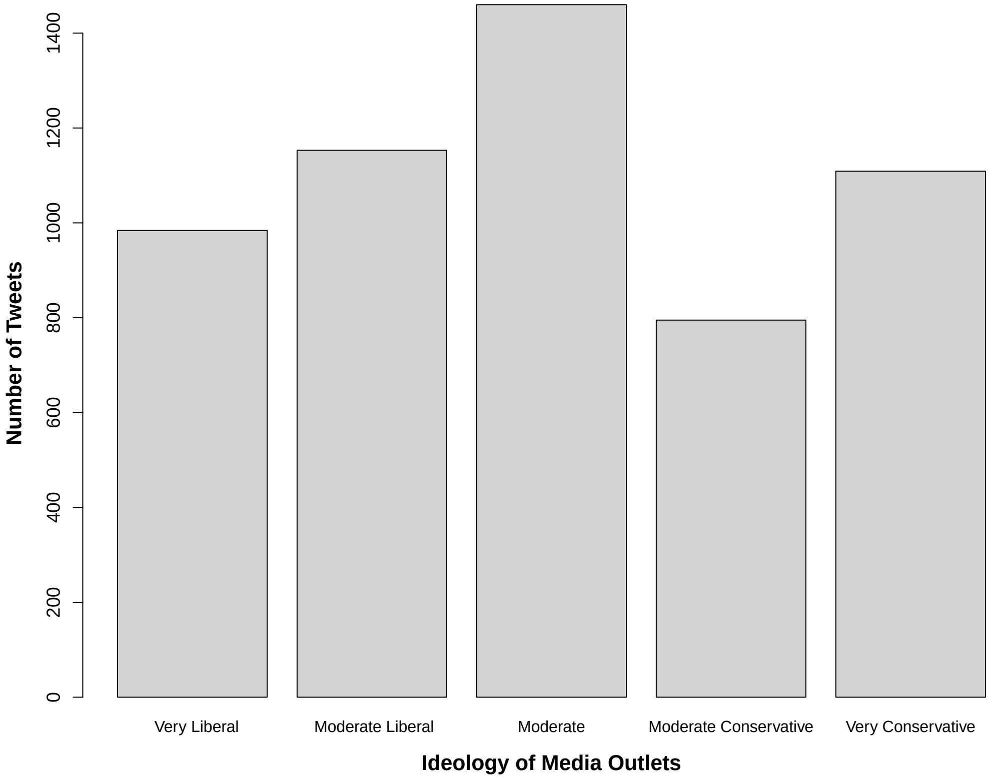

Fig. S.2: Number of images used in tweets across media outlets with different ideological standpoints.

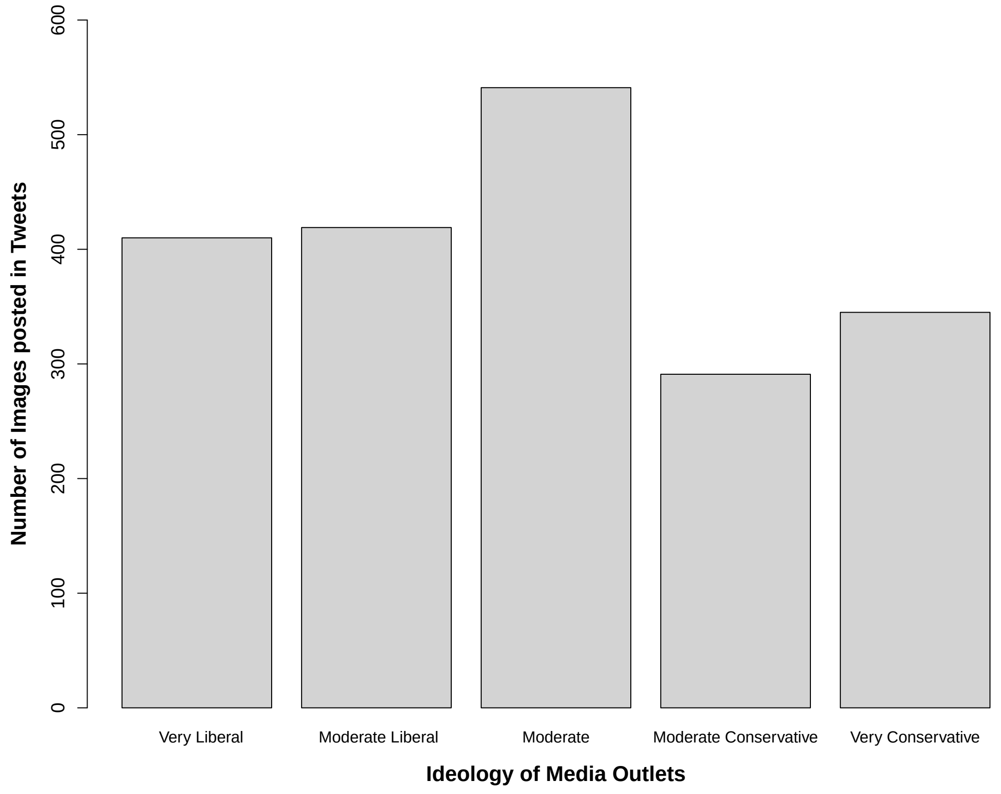

Supplement: S1 Appendix — (ZIP) [file pone.0331219.s001.zip › si_files/S2_Appendix.pdf]
