# Supplementary material for: Media choice and audience perceptions: Evidence from visual framing of immigration in news stories
Source: PLoS One. 2025 Sep 15;20(9):e0331219. doi: 10.1371/journal.pone.0331219 (PMC12435698; doi:10.1371/journal.pone.0331219)
Supplement: S1 Appendix — (ZIP) [file pone.0331219.s001.zip › si_files/S5_Appendix.pdf]

S5 Robustness Checks

S5.1 Alternative Measure of Outlet Ideology

To provide evidence that our results are robust to a different measure of outlets' ideology, we use another source of data for media outlets' slant, which is Media Bias Fact Check.[1] We use these new bias labels which we organize into three groups: ”Left”, ”Least Biased” (meaning the most moderate), and ”Right”. We then check the associations between visual frames derived from 2006 images (1303 images after excluding the “Other” category) and the three outlet's ideology levels. The results are presented in Figure S.8.

Here the media outlets' ideology is measured on a three-point scale: left-leaning, least biased (similar to moderate), and right-leaning outlets. As we can see on the mosaic plot, the results are consistent with the main findings (in the main text) and indicate that left-leaning outlets are more likely to use visual frames of 'women and children' when talking about migrant caravans, whereas right-leaning outlets use this visual frame much less. Instead, they are more likely to portray stories about migrant caravans through images of 'crowds' and 'violations'.

[1]The Source is at https://mediabiasfactcheck.com

Fig. S.8: Visual frames and ideology of media outlets: Alternative ideology measure.

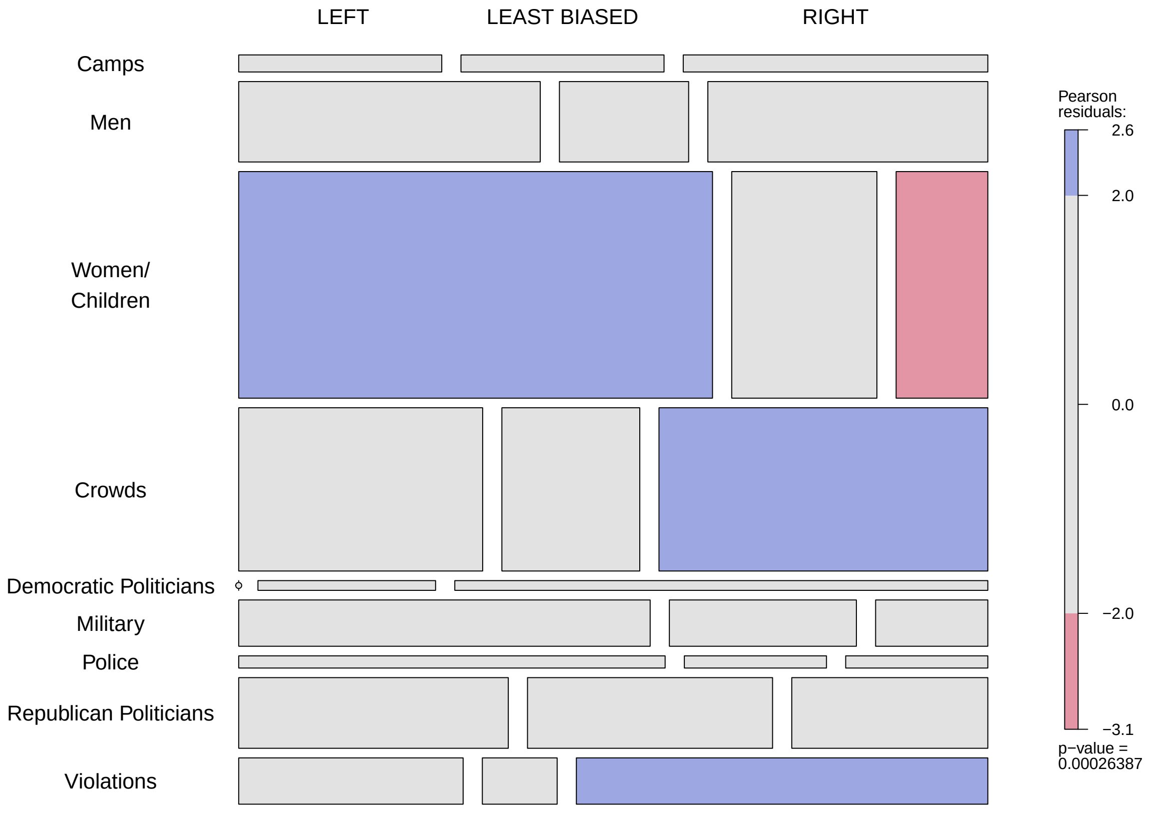

Note: This plot shows the relationship between two nominal variables of interest: (1) media outlet ideology (ranging from very left-leaning to very right-leaning) and (2) image cluster. Colors indicate both the direction and strength of associations between categories, as measured by Pearson standardized residuals—the deviation of observed counts from those expected under independence. Blue shading denotes positive associations (more cases than expected), red shading denotes negative associations (fewer cases than expected), and gray indicates no meaningful association. The p-value displayed corresponds to a Chi-square test of independence and rejects the null hypothesis of no association between the two variables.

S5.2 Alternative Search of Images

We also need to check whether our results are robust to other images on a similar topic. Our initial set of images have been shaped by querying tweets mentioning "migrant caravan" connotation. We now narrow the search timeline by looking at 3 months periods around first (February 15, 2018 - May 15, 2018) and second caravans (September 15, 2018 - December 15, 2018) - the most prominent caravans. Additionally, instead of using "migrant caravan" connotation, we queried tweets and corresponding images based on the appearance of any of the following words in tweets: ″migrant,″ ″caravan,″ ″migrants,″ ″caravans″. Such a query results in 14594 tweets (excluding duplicates of the tweets for each of the keyword search) and 5149 images that accompany these tweets. Table S.5 summarizes the returned results for the collected tweets:

Table S.5: Returned tweets for each of the key words searches.

Term	Count
migrant	10,163
migrants	5,194
caravan	7,086
caravans	447
migrant caravan	3,060

We ask trained coders to label these images using our curated codebook and assign one of 10 labels to all the images. We use the majority rule intercoders agreement to assign final labels to each of the images. If there is a lack of agreement between the coders, we leave images as unlabelled and exclude them from the final analysis.

We estimate a association between media outlets ideology and image labels for all images with meaningful labels (excluding the "Other" category and images with no agreement on labels; total of 2447 images). The results of this association are shown in Figure S.9. Not only do they support the original findings, but they also amplify them. Here we observe that left-leaning outlets tend to use frames of ′women and children′ significantly more often than ′men,′ and ′violations,′ which they tend to ignore. In contrast,

right-leaning media portray immigrants as 'crowds,' 'police,' and 'violations' rather than 'women and children'. They also use more images of politicians, both Republican and Democratic.

Fig. S.9: Visual frames and ideology of media outlets: Alternative image search.

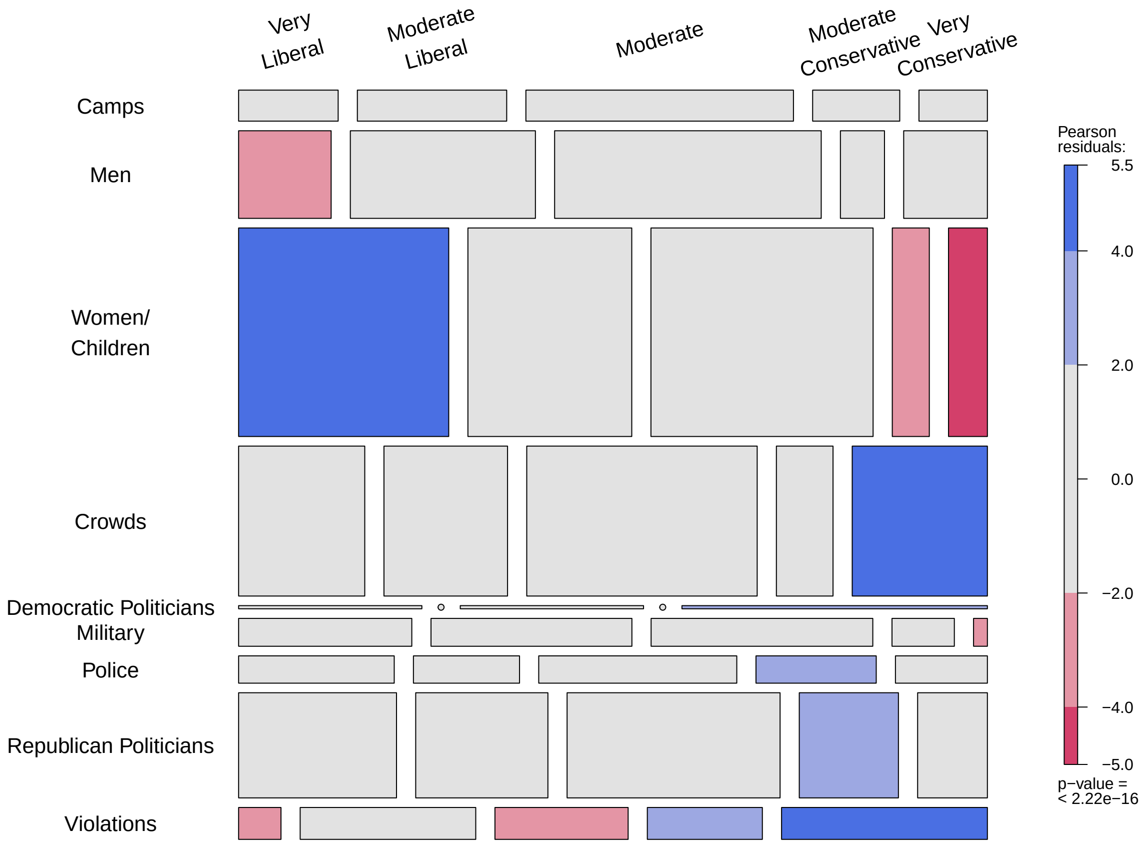

Note: This plot shows the relationship between two nominal variables of interest: (1) media outlet ideology (from very left-leaning/very liberal to very right-leaning/very conservative) and (2) image cluster. Colors indicate both the direction and strength of associations between categories. The strength of each association is measured by Pearson standardized residuals, which quantify deviations of observed counts from those expected under independence. Blue shading denotes positive associations (more cases than expected), red shading denotes negative associations (fewer cases than expected), and gray signifies no meaningful association. The p-value displayed corresponds to a Chi-square test of independence, rejecting the null hypothesis of no association between the two variables.

S5.3 Only 2018

Since the overall data that we analyze covers 2017-2021, some variation of visual representations chosen by media outlets potentially can be explained by variation in U.S. administration at the time. To validate that our results are not affected by that, we run a cross-tabulation analysis only for the year 2018. This is the time period when the largest caravans were happening anyways, and when we have a consistency of presidential administration. With that we also eliminate potential indirect effects of other important events happening post-2018 (such as COVID-19 and presidential elections of 2020). The results for 2018 time period are presented in Table S.10 and are consistent with the baseline results in the main text.

Fig. S.10: Visual frames and ideology of media outlets (only in 2018).

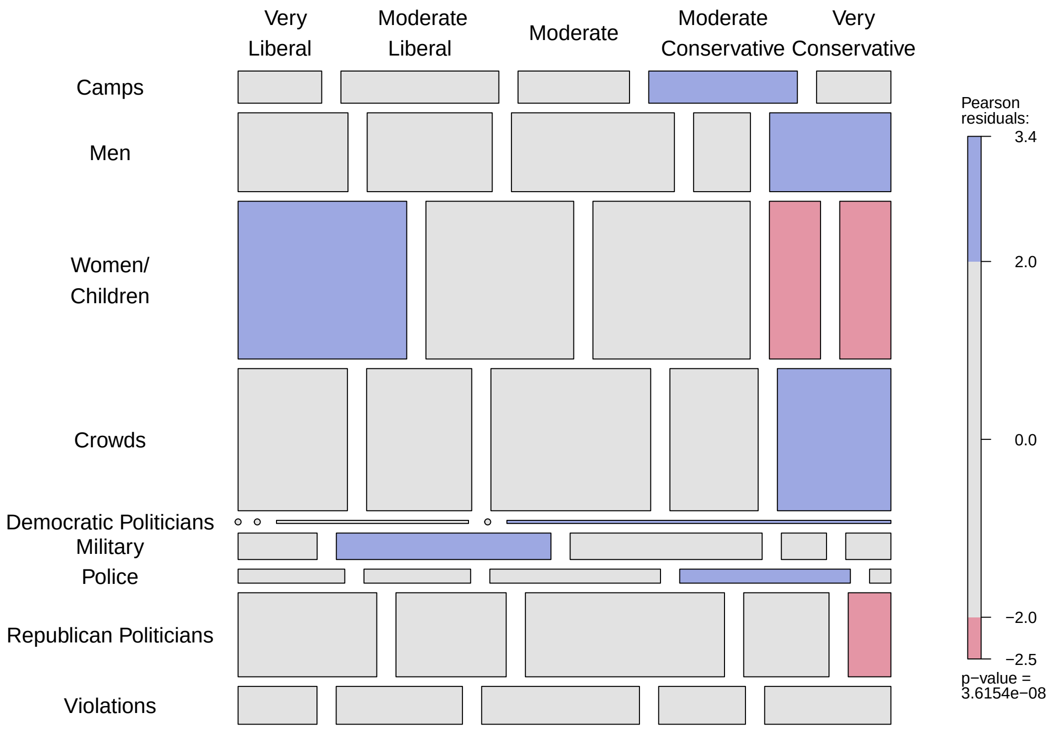

Note: This plot shows the relationship between two nominal variables of interest: (1) media outlet ideology (from very left-leaning to very right-leaning) and (2) image cluster. Colors indicate both the direction and strength of associations between categories. The strength of each association is measured by Pearson standardized residuals, which quantify how much observed counts deviate from those expected under independence. Blue shading denotes positive associations (more cases than expected), red shading denotes negative associations (fewer cases than expected), and gray indicates no meaningful association. The displayed p-value corresponds to a Chi-square test of independence and rejects the null hypothesis of no association between the two variables.
