# Supplementary material for: Media choice and audience perceptions: Evidence from visual framing of immigration in news stories
Source: PLoS One. 2025 Sep 15;20(9):e0331219. doi: 10.1371/journal.pone.0331219 (PMC12435698; doi:10.1371/journal.pone.0331219)
Supplement: S1 Appendix — (ZIP) [file pone.0331219.s001.zip › si_files/S3_Appendix.pdf]

S3 Unsupervised Clustering

For the unsupervised classification task, we work with all the images that have been directly uploaded to Twitter posts; and we exclude the preview images since they generally contain more noise.

To perform an unsupervised image clustering, we utilize the weights of ResNet-50 neural network architecture trained on the ImageNet data for image features extraction and apply an unsupervised K-means algorithm for image clustering. K-means clustering requires us to choose the number of clusters as an *ex-ante* parameter. We used elbow and silhouette methods applied to the set of extracted features to identify the optimal number of clusters. Figure S.3 indicates that the optimal number of topics/clusters equals 7. Therefore, we perform a K-means clustering with K=7 clusters. Table S.4 shows distribution of images across clusters, with the total number of 2009 images. Figures S.4 provide examples of randomly selected images from several returned clusters. Unsupervised clustering does not rely on image labels; instead, it groups images that appear most similar based on various characteristics such as colors, shapes, shades, and angles. The task of assigning substantive topics is left to researchers. Due to this uncurated approach, some clusters may contain illogical image combinations, as illustrated in Figure S.5 — cluster 1 example includes border images but also included images of tweets; cluster 3 of images with groups of migrants erroneously included an image of a politician, cluster 6 with politicians and news figures also includes an image of an immigrant woman with a child.

Table S.4: Distribution of images across clusters.

	Number of Images	Proposed Cluster Topic
Cluster 1	208	Border/Night Views
Cluster 2	188	News Anchors/Public Speakers
Cluster 3	110	Crowds/Camps
Cluster 4	499	Groups of People
Cluster 5	107	Public Speakers
Cluster 6	590	Politicians/News Anchors
Cluster 7	307	Enforcement/Crowds

Fig. S.3: Optimal number of K clusters testing.

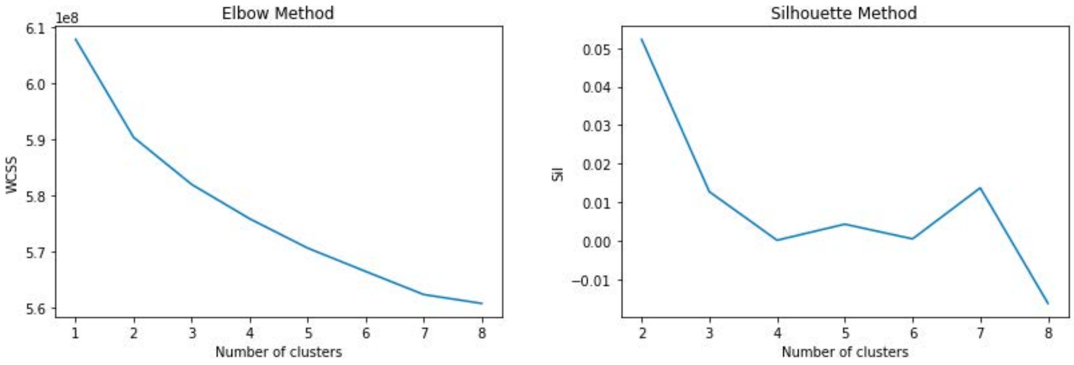

Fig. S.4: Examples of images from K-means clusters.

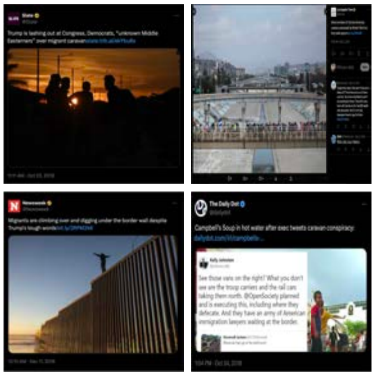

(a) Cluster 1

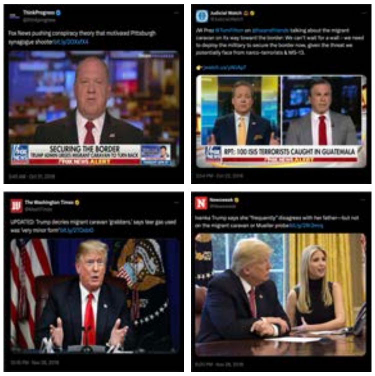

(b) Cluster 2

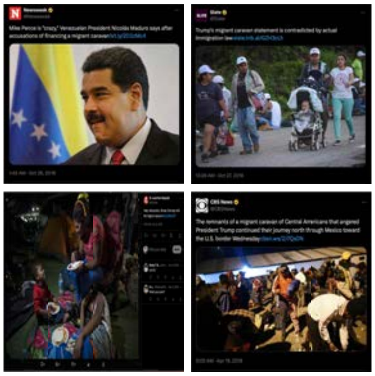

(c) Cluster 3

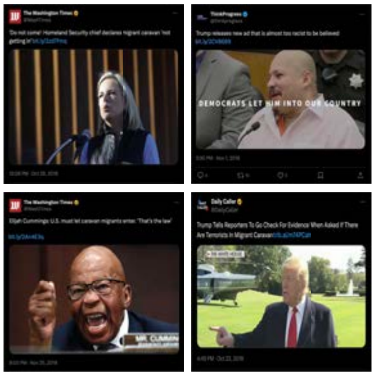

(d) Cluster 5

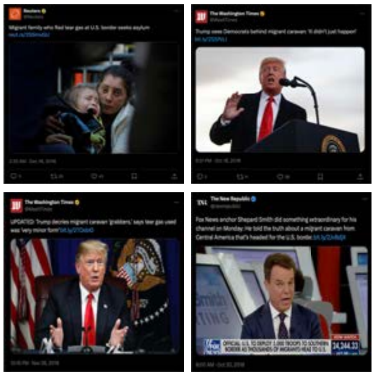

(e) Cluster 6

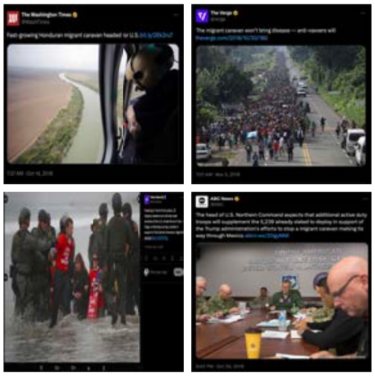

(f) Cluster 7

Fig. S.5: Examples of unsupervised clustering errors

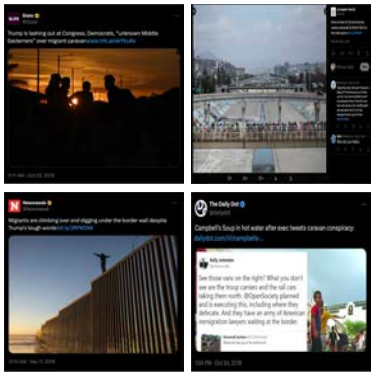

(a) Cluster 1

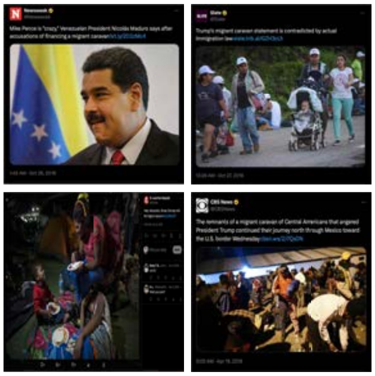

(b) Cluster 3

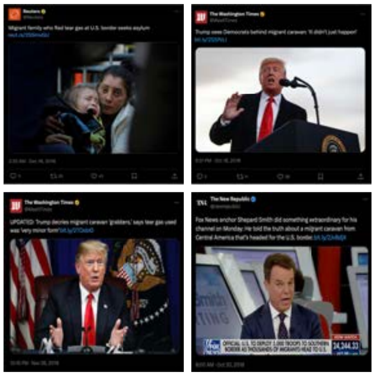

(c) Cluster 6
