# Supplementary material for: Media choice and audience perceptions: Evidence from visual framing of immigration in news stories
Source: PLoS One. 2025 Sep 15;20(9):e0331219. doi: 10.1371/journal.pone.0331219 (PMC12435698; doi:10.1371/journal.pone.0331219)
Supplement: S1 Appendix — (ZIP) [file pone.0331219.s001.zip › si_files/S15_Appendix.pdf]

S15 Correlations between Outlet Ideology and Outlet Ideology Guesses

In this section, we report correlation matrices showing how Democrats' and Republicans' ideological guesses about media outlets relate to the actual likelihood of an image being from a left- or right-leaning source. To compute these correlations, we first construct scoring variables based on respondents' ideological guesses for each image. Each respondent provides a binary evaluation: 0 indicates a guess that the image is from a conservative outlet, and 1 indicates a guess that it is from a liberal outlet. For each image, we average these binary values across all respondents who evaluated it, yielding a probability score representing the likelihood that the image is guessed to be from a liberal outlet. We subtract this value from 1 to obtain the complementary probability that the image is guessed to be from a conservative outlet. These two scores are naturally complementary and sum to one.

To estimate the actual probability of an image originating from a liberal or conservative outlet, we adopt a frame-based approach. Specifically, we use known probabilities of each of the nine visual frames being associated with left- or right-leaning media. Since each image is labeled with a specific visual frame, we apply these frame-level probabilities to assign an estimated likelihood that the image was published by a liberal or conservative outlet.

We then compute correlations among the four resulting variables: (1) the estimated probability that an image is from a liberal outlet, (2) the estimated probability that it is from a conservative outlet, (3) the average guessed probability that it is from a liberal outlet, and (4) the average guessed probability that it is from a conservative outlet. These correlations are reported separately for Democrats and Republicans in Tables S.24 and S.25. The results show that correlations between respondents' guesses and the actual outlet tendencies are generally weak (not exceeding $\rho = 0.3$).

Table S.24: Scoring correlation table: Democrats.

	Outlet: Prop Liberal	Outlet: Prop Conservative
Respondent Guess: Prop Liberal	0.302	-0.001
Respondent Guess: Prop Conservative	-0.302	0.001

Table S.25: Scoring correlation table: Republicans.

	Outlet: Prop Liberal	Outlet: Prop Conservative
Respondent Guess: Prop Liberal	0.261	0.164
Respondent Guess: Prop Conservative	-0.261	-0.164

S16 Curated Labeling

Table S.26 presents the distribution of curated labels across all 2,006 harvested images and Table S.27 shows distribution of curated labels for images used in the survey wave. The ”Other” category is a residual group that includes images containing text, multiple aligned images of political figures, and images with logos (e.g., news channel logos). The ”Undefined” category includes images for which coders did not reach agreement.

Table S.26: Curated labels cluster sizes.

Curated Label	Number of Images
Camps	68
Close Shots (Men)	186
Close Shots (Women/Children)	349
Crowds	338
Democratic Politicians	7
Military	59
Police	33
Republican Politicians	170
Violations	93
Other	602
Undefined	101

In Figure S.16 we demonstrate examples of curated labels, and the way how such a curated labeling potentially can improve classification task.
